# Supplementary material for: Photochemical synthesis of natural lipids in artificial and living cells
Source: Nat Commun. 2025 May 31;16:5068. doi: 10.1038/s41467-025-60358-4 (PMC12126527; doi:10.1038/s41467-025-60358-4)
Supplement: Supplementary file 1 — Supplementary Information [file 41467_2025_60358_MOESM1_ESM.pdf]

Supplementary information

Photochemical synthesis of natural lipids in artificial and living cells

Peng Ji, Alexander Harjung, Caroline Knittel, Alessandro Fracassi, Jiyue Chen, Roberto J. Brea, Neal K. Devaraj*

Correspondence to: ndevaraj@ucsd.edu.

This PDF file includes:

Materials and Methods
Supplementary Text
Supplementary Figs. 1 to 71
Supplementary Tables 1 to 5
Copies of NMR spectra
Supplementary References

Other Supplementary Materials for this manuscript include the following:

Supplementary Movies 1 to 6

Table of Contents

1. General materials and instrumentation ………………………………………………S3
2. Synthesis of starting materials ……..………………………..…………………...………S4
3. Optimization of photoredox lipid ligation (PLL)....……………………………..….…..……S15
4. Synthesis of different lipids by PLL …….……………………………………………..S17
5. NHPI **2a** stability …...…………………………………………..………………...…S25
6. Reaction of non-amphiphilic acrylate with NHPI ester **2a** ………………………...……...S26
7. Reaction kinetics of POPC synthesis under green light irradiation ...………………..S26
8. In situ POPC (**3b**) vesicle growth under green light ….………………………….…….S29
9. Encapsulation of fluorescent dyes or proteins during de novo vesicle formation …...S30
10. Cryogenic electron microscopy (cryoEM) images of vesicles formed by PLL …….S32
11. De novo formed OPPC vesicles that have spontaneously entrapped eosin Y can catalyze new phospholipid synthesis ……………………………………..……………..…S33
12. De novo formed OPPC vesicles that have spontaneously entrapped eosin Y can catalyze new non-canonical phospholipid synthesis ………………………………………S35
13. De novo formation of POPC:DPPC mixed vesicles under green light ……………...S37
14. In situ POPC vesicle formation using a fluorogenic nucleic acid binding dye …...…S38
15. Nucleic acid design and sequences ……………………………..…………..…S41
16. HeLa cell membrane labeling by PLL ..………………………..…………………S42
17. HRMS quantification of ceramide synthesis in live cells ...…………………..….…S44
18. Cell proliferation assay …………………………………………………...……S46
19. Western blot assay ……………………………………………...…………..…S48
20. NMR spectra ……………………………………………………………………..S50
21. Supplementary References ………………...……………………………………..S73

1. General materials and instrumentation

All reagents and solvents were purchased from Sigma-Aldrich and Fisher Scientific. All C18:1-lyso-PC, C16:0-lyso-PC, C18:0-lyso-PC, D-erythro-sphingosine, Lyso SM (d18:1), sphingosine (d18:1), and C16 ceramide were purchased from Avanti® Polar lipids (Croda International Plc., Alabaster, Alabama, USA). BuNAH was purchased from AstaTech, Inc. Deuterated chloroform ($CDCl_3$), methanol (CD_3OD), and dimethyl sulfoxide (DMSO-d_6) were obtained from Cambridge Isotope Laboratories. Water was purified by a Millipore purification system (Merck KGaA). Thin-layer chromatography (TLC) was performed using silica gel pre-coated plastic sheets (Polygram SIL G/UV254, 0.2 mm, with fluorescent indicator; Macherey-Nagel), and were visualized with a UV lamp (254 nm), or potassium permanganate stain (1.5 g of $KMnO_4$, 10 g K_2CO_3, and 1.25 mL 10% NaOH in 200 mL H_2O). Column chromatography was carried out using Merck silica gel (60 Å, 230−400 mesh, particle size 0.040−0.063 mm) using technical grade solvents. Elution was accelerated using compressed air. NMR spectra were measured on a Jeol ECA-400. The solvent employed and respective measuring frequency are indicated for each experiment. Chemical shifts are reported with tetramethylsilane (TMS) serving as a universal reference for all nuclides. The resonance multiplicity is described as s (singlet), d (doublet), t (triplet), q (quadruplet), m (multiplet), and br (broad). All spectra were recorded at 298 K unless otherwise noted, processed with MestReNova 14.2.3 suite, and coupling constants are reported as observed. The residual deuterated solvent signal relative to tetramethylsilane is used as the internal reference in ^{1}H NMR spectra (e.g. $CDCl_3$ = 7.26 ppm) and is reported as follows: chemical shift in ppm (multiplicity, coupling constant J in Hz, number of protons). Solvent mixtures for chromatography are reported as volume/volume (v/v) ratios.

Green LED lights were purchased from HepatoChem (EvoluChem 525 PF, Cat. No. HCK1012-01-004; UltraFire Green Hunting Flashlight, 650 Lumens, Single Mode, 520-535 nm Wavelength). Blue LEDs were purchased from Amazon (Kessil A160WE Tuna Blue LED Light) or were part of the Wisconsin Photoreactor Platform[1] equipped with blue LEDs, (Cree, Inc. XTEARY-00-0000-000000N09, CREEXTE-ROY-3), maximum emission wavelength (448 nm).

High performance liquid chromatography (HPLC) analysis was carried out using an Agilent 1260 Infinity Series HPLC (Agilent Technologies, Waldbronn, Germany) with an Agilent Zorbax Eclipse Plus C8 analytical column with Phase A/Phase B gradients [Phase A: H_2O with 0.1% formic acid; Phase B: MeOH with 0.1% formic acid], at a flow rate of 1 mL/min. The HPLC was equipped with a UV-vis detector, a 380 Varian-Agilent evaporative light scattering detector (ELSD), and a 6120 Agilent Quadrupole mass spectrometer (MS).

HPLC-HRMS spectra were recorded on a Q Exactive Orbitrap mass spectrometer (Thermo Fisher Scientific, Waltham, Massachusetts, USA) connected to a Vanquish Flex Analytical UHPLC System (Thermo Fisher Scientific, Waltham, Massachusetts, USA) equipped with a C8 column (100 x 2.1 mm, particle size 1.9 µm). HPLC-HRMS chromatograms were obtained with a solvent gradient of 0.1% formic acid in water (Solvent A) and 0.1% formic acid in methanol (Solvent B).

The solvent gradient was: 0-1 min 30% B, 1-4 min 30%-90% B, 4 - 15 min 99%, 15- 18 min 99% B, 18 - 20 min 30% B.

Microscopy images were acquired using an Olympus BX51 optical microscope equipped with a phase contrast condenser (Ph3) and a 100x, 1.30 NA oil immersion objective. Images were captured with an ORCA-spark Digital CMOS camera (C11440-36U, Hamamatsu) using Olympus cellSens 3.1 imaging software. Spinning-disk confocal microscopy images were acquired on a Yokagawa spinning disk system (Yokagawa, Japan) built around an Axio Observer Z1 motorized inverted microscope (Carl Zeiss Microscopy GmbH, Germany) with a 63x, 1.40 NA oil immersion objective to an ORCAFlash 4.0 V2 Digital CMOS camera (Hamamatsu, Japan), using ZEN Blue imaging software (Carl Zeiss Microscopy GmbH, Germany). The fluorophores were excited with diode lasers (405 nm, 488 nm, 561 nm, and 638 nm). Images were further analyzed with ImageJ software (version 2.1.0). The samples were typically prepared by depositing 2 μL of the desired lipid dispersion on a microscope glass slide (Fisher Scientific, Cat. No: 12550C; 75x50x1.0 mm), and adding a coverslip (VWR micro cover glass, Cat. No. 48366-045, 0.13 to 0.17 mm thickness, 18x18 mm) on top. The images obtained were further analyzed with ImageJ software.

2. Synthesis of starting materials

*Synthesis of 1-decyl-1,4-dihydropyridine-3-carboxamide (decyl-NAH **S4**):*

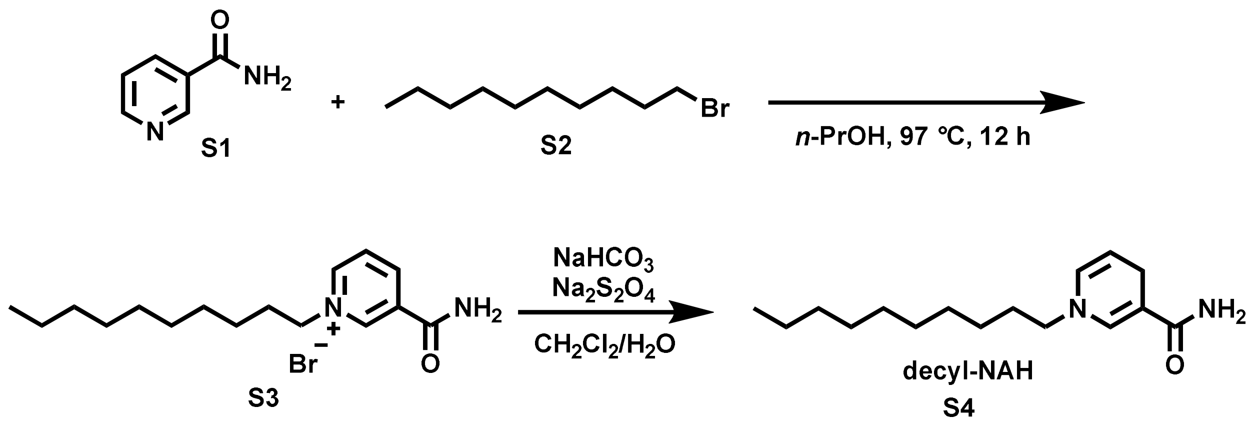

Synthesis of the compound **S3**:

To a dry 100 mL round-bottom flask nicotinamide **S1** (5.0 g, 40.94 mmol, 1.0 equiv) and 10-bromododecane **S2** (9.59 g, 42.99 mmol, 1.05 equiv) were added and dissolved in 50 mL *n*-PrOH. The reaction mixture was stirred at 97 °C overnight. The resulting product was filtered by a Büchner funnel and washed with cold *n*-PrOH, yielding 1-decyl-3-carbamoylpyridinium bromide **S3** as a white solid (12 g, 85%).

^{1}H NMR (400 MHz, DMSO-d_6) δ 9.54 (d, J = 1.6 Hz, 1H), 9.25 (dt, J = 6.1, 1.3 Hz, 1H), 8.95 (dt, J = 8.1, 1.4 Hz, 1H), 8.60 (s, 1H), 8.27 (dd, J = 8.1, 6.1 Hz, 1H), 8.17 (s, 1H), 4.66 (t, J = 7.5 Hz, 2H), 1.94 (t, J = 7.3 Hz, 2H), 1.25 (d, J = 19.8 Hz, 14H), 0.84 (t, 3H).

^{13}**C NMR** (100 MHz, DMSO-d_6) δ 162.86, 146.40, 144.78, 143.38, 133.82, 127.88, 61.12, 31.29, 30.66, 28.89, 28.79, 28.67, 28.41, 25.41, 22.11, 13.98.

HRMS (ESI) m/z calcd. for $C_{16}H_{27}N_2O$ $[M]^+$ 263.2118, found 263.2119.

Synthesis of the compound **S4**:
Under nitrogen atmosphere, 200 mL of distilled water and 100 mL of CH_2Cl_2 were added to a dry 500 mL round-bottom flask and cooled to 0 °C. Subsequently, compound **S3** (3.5 g, 10.19 mmol, 1.0 equiv) and $NaHCO_3$ (5.14 g, 61.17 mmol, 6.0 equiv) were added, followed by the gradual addition of sodium dithionite (7.1 g, 40.78 mmol, 4.0 equiv) in small portions over 10 min. The reaction mixture was stirred in the dark for 2 h at 0 °C. Upon completion, the organic phase was separated, washed with cold water, dried over $MgSO_4$, and the solvent removed to give the crude product, which was further purified by crystallization in MeOH/H_2O, yielding a light-yellow solid **S4** (350 mg, 13% yield).

^{1}**H NMR** (400 MHz, CD_3OD) δ 6.99 (d, J = 1.6 Hz, 1H), 5.82 (dd, J = 8.0, 1.7 Hz, 1H), 4.75 (dt, J = 8.0, 3.5 Hz, 1H), 3.13 (t, J = 7.0 Hz, 2H), 3.09 (dd, J = 3.5, 1.7 Hz, 2H), 1.54 (t, J = 6.9 Hz, 2H), 1.31 (dt, J = 11.0, 4.1 Hz, 14H), 0.90 (t, 3H).

^{13}**C NMR** (100 MHz, CD_3OD) δ 173.77, 141.36, 129.99, 104.13, 98.69, 54.82, 33.07, 31.05, 30.67, 30.64, 30.43, 30.40, 27.43, 23.73, 23.35, 14.44.

HRMS (ESI) m/z calcd. for $C_{16}H_{28}N_2O$ $[M+H]^+$ 265.2274, found 265.2277.

General procedure for the synthesis of lysolipid 1a-1c:

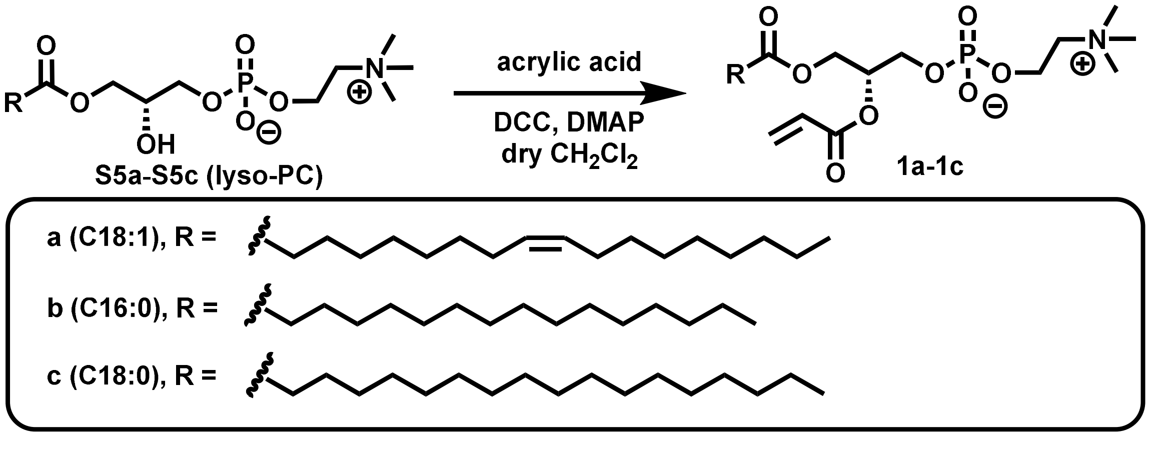

To a 2-dram glass vial, lyso-PC **S5a-S5c** (1.0 equiv), DMAP (1.0 equiv), and acrylic acid (3.0 equiv) were added in 1 mL dry CH_2Cl_2. Subsequently, DCC (3.0 equiv) was added, and the reaction was stirred under N_2 atmosphere for 24-120 h. Upon reaction completion, as confirmed by HPLC-ELSD-MS, the solution was filtered and concentrated by rotary evaporation. The resulting crude product was purified by silica gel column chromatography using $CHCl_3$/MeOH/H_2O (50/50/2–20/80/2) as eluents.

*Synthesis of (R)-2-(acryloyloxy)-3-(oleoyloxy)propyl (2-(trimethylammonio)ethyl) phosphate (**1a**)*

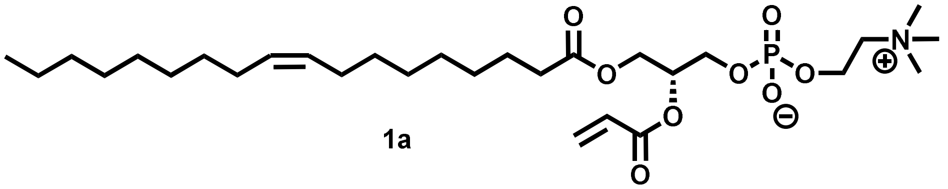

According to the general procedure, compound **1a** (44 mg, 40%) was obtained as a colorless oil using C18:1-lyso-PC **S5a** (100 mg, 0.192 mmol, 1 equiv), acrylic acid (40 µL, 0.575 µL, 3 equiv), DCC (120 mg, 0.575 mmol, 3 equiv), and DMAP (23.4 mg, 0.192 mmol, 1 equiv), stirred for 24 h.

^{1}H NMR (400 MHz, $CDCl_3$) δ 6.41 (d, *J* = 17.3 Hz, 1H), 6.12 (dd, *J* = 17.3, 10.4 Hz, 1H), 5.87 (d, *J* = 10.2 Hz, 1H), 5.57 – 4.96 (m, 3H), 4.59 – 4.28 (m, 3H), 4.21 (dd, *J* = 12.1, 7.0 Hz, 1H), 4.03 (s, 2H), 3.81 (s, 2H), 3.35 (s, 9H), 2.28 (t, *J* = 7.7 Hz, 2H), 2.00 (q, *J* = 6.5 Hz, 4H), 1.57 (d, *J* = 9.7 Hz, 2H), 1.28 (dd, *J* = 8.6, 4.9 Hz, 20H), 0.87 (t, *J* = 6.7 Hz, 3H).

^{13}C NMR (100 MHz, $CDCl_3$) δ 173.72, 165.62, 132.04, 130.16, 129.85, 128.14, 77.36, 70.83, 66.37, 64.14, 62.63, 59.79, 54.52, 34.17, 32.04, 29.90, 29.87, 29.66, 29.46 (2C), 29.35, 29.29, 29.23, 27.36, 27.32, 24.97, 22.82, 14.27.

HRMS (ESI) m/z calcd. for $C_{29}H_{54}NO_8P$ $[M+H]^+$ 576.3660, found 576.3669.

*(R)-2-hydroxy-3-(palmitoyloxy)propyl (2-(trimethylammonio)ethyl) phosphate (**1b**)*

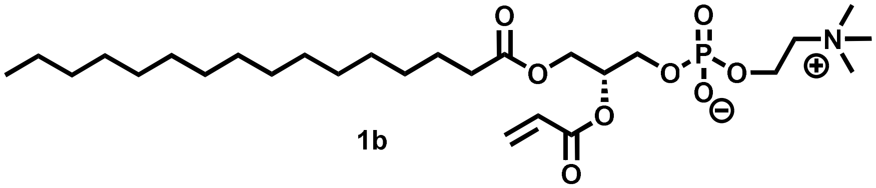

According to the general procedure, compound **1b** was obtained as a colorless oil (52 mg, 38%) using C16:0-lyso-PC **S5b** (125 mg, 0.252 mmol, 1 equiv), acrylic acid (52 µL, 0.756 mmol, 3 equiv), DCC (156 mg, 0.756 mmol, 3 equiv), and DMAP (31 mg, 0.252 mmol, 1 equiv), stirred for 120 h.

^{1}H NMR (400 MHz, CD_3OD) δ 6.42 (dd, *J* = 17.3, 1.5 Hz, 1H), 6.17 (dd, *J* = 17.3, 10.4 Hz, 1H), 5.92 (dd, *J* = 10.4, 1.5 Hz, 1H), 5.52 – 5.11 (m, 1H), 4.42 (dd, *J* = 12.0, 3.5 Hz, 1H), 4.25 (dd, *J* = 12.0, 6.9 Hz, 3H), 4.05 (t, *J* = 5.6 Hz, 2H), 3.80 – 3.52 (m, 2H), 3.22 (s, 9H), 2.31 (t, *J* = 7.4 Hz, 2H), 1.59 (d, *J* = 7.4 Hz, 2H), 1.46 – 1.18 (m, 24H), 0.90 (t, *J* = 6.9 Hz, 3H).

^{13}C NMR (100 MHz, CD_3OD) δ 174.97, 166.83, 132.37, 129.30, 72.20, 67.48, 64.88, 63.55, 60.51, 54.71, 54.67, 54.63, 34.83, 33.09, 30.81, 30.80, 30.78, 30.74, 30.63, 30.61, 30.53, 30.49, 30.43, 30.18, 25.98, 23.75, 14.45.

HRMS (ESI) m/z calcd. for $C_{27}H_{52}NO_8P$ $[M+H]^+$ 550.3503, found 550.3511.

*(R)-1-(((2-(acryloyloxy)-3-(stearoyloxy)propoxy)oxidophosphoryl)oxy)-2-(trimethylammonio)ethan-1-ide (**1c**)*

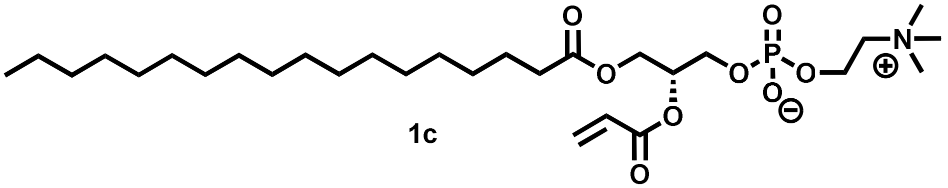

1c

According to the general procedure, compound **1c** (40 mg, 36%) was obtained as a colorless oil (40 mg, 36%) using C18:0-lyso-PC **S5c** (102 mg, 0.195 mmol, 1 equiv), acrylic acid (40 µL, 0.584 mmol, 3 equiv), DCC (121 mg, 0.584 mmol, 3 equiv), and DMAP (24 mg, 0.195 mmol, 1 equiv), stirred for 120 h.

^{1}H NMR (400 MHz, $CDCl_3$) δ 6.41 (d, *J* = 17.3 Hz, 1H), 6.12 (dd, *J* = 17.3, 10.4 Hz, 1H), 5.88 (d, *J* = 11.8 Hz, 1H), 5.29 (s, 1H), 4.72 – 4.29 (m, 2H), 4.29 – 4.13 (m, 2H), 4.03 (s, 2H), 3.84 (s, 2H), 3.36 (s, 9H), 2.27 (t, *J* = 7.6 Hz, 2H), 1.56 (t, *J* = 7.3 Hz, 2H), 1.25 (d, *J* = 2.2 Hz, 28H), 0.87 (t, *J* = 6.8 Hz, 3H).

^{13}C NMR (100 MHz, $CDCl_3$) δ 173.71, 165.61, 132.02, 128.22, 71.02, 66.25, 64.04, 62.75, 59.79, 54.49, 34.22, 32.07, 29.89, 29.88, 29.86 (4C), 29.83, 29.81, 29.67, 29.51, 29.46, 29.30, 25.01, 22.84, 14.27.

HRMS (ESI) m/z calcd. for $C_{29}H_{56}NO_8P$ $[M+H]^+$ 578.3816, found 578.3822.

*Synthesis of (R)-2-(acryloyloxy)-3-(phosphonooxy)propyl palmitate (**1d**)*

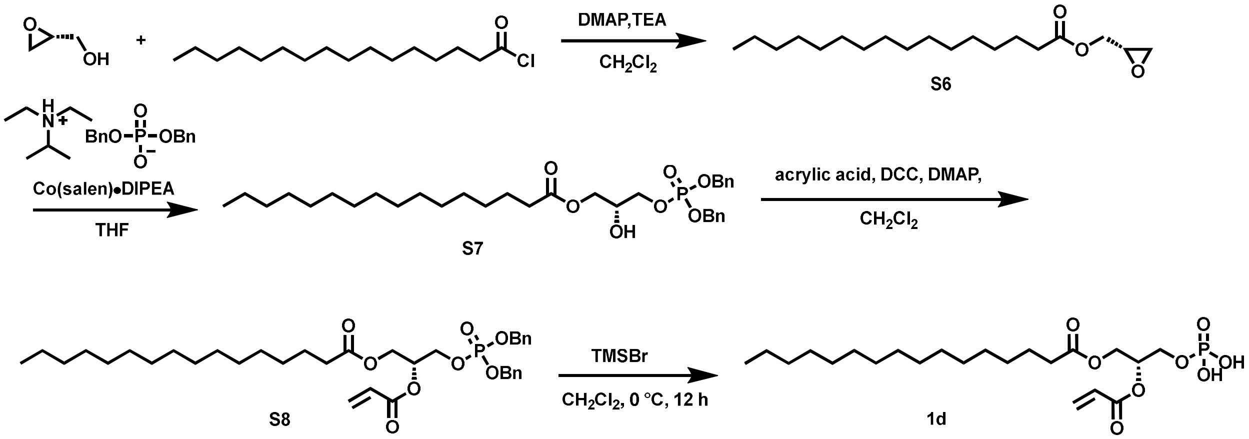

Synthesis of the compound **S6**:

The synthesis was adapted from a previously published procedure[2]. To a solution of (*R*)-oxiran-2-ylmethanol (0.5 g, 6.75 mmol, 1.0 equiv) and triethylamine (1.13 mL, 8.1 mmol, 1.2 equiv) in dry

CH_2Cl_2 (20 mL) cooled at 0 °C, palmitoyl chloride (2.45 mL, 8 mmol, 1.2 equiv) was added dropwise. The mixture was stirred for 16 h, allowing it to warm up to rt. Water (10 mL) was then added, and the mixture was transferred to a separatory funnel. The aqueous phase was extracted with EtOAc (3x), the organic layers were combined and dried over $MgSO_4$, filtered, and concentrated in vacuo. The crude product was further purified by silica flash column chromatography, using hexane/ethyl acetate (3/1) as the eluent, which provided compound **S6** as a white solid (1.86 g, 88%). The NMR data is consistent with the previously reported data[2].

Synthesis of the compound **S7**:
Synthesis was adapted from a previously published procedure[2]. To a vial containing a stir bar, dibenzylphosphate•*i*-Pr_2NEt salt (0.868 g, 1.95 mmol, 1 equiv) and the oxirane **S6** (600 mg, 1.95 mmol, 1 equiv) were added in dry THF (2 mL) under N_2 atmosphere. Co^{III}(salen) catalyst I (0.195 mmol, 10 mol%) was then added, and the resulting green mixture was stirred for 16 h under N_2 atmosphere. The mixture was purified by silica gel column chromatography using hexane/ethyl acetate (1/1) as the eluent, which yielded the compound **S7** as a viscous oil (413 mg, 36%). The NMR data is consistent with the previously reported data[2].

Synthesis of the compound **S8**:
To a 50 mL round-bottom flask, (*R*)-3-((bis(benzyloxy)phosphoryl)oxy)-2-hydroxypropyl palmitate **S7** (328 mg, 0.56 mmol, 1 equiv), acrylic acid (115 µL, 1.67 mmol, 3 equiv), DCC (345 mg, 1.67 mmol, 3 equiv), and DMAP (67 mg, 0.056 mmol, 1 equiv) were added in dry CH_2Cl_2,and the mixture was stirred at rt for 2 days. After the reaction was completed, the crude solution was filtered, purified by silica gel column chromatography using hexane/ethyl acetate (10/1) as the eluent, which yielded compound **S8** as a colorless oil (150 mg, 46%).

^{1}H NMR (400 MHz, $CDCl_3$) δ 7.34 (t, *J* = 2.0 Hz, 10H), 6.41 (dt, *J* = 17.4, 1.1 Hz, 1H), 6.07 (ddd, *J* = 17.3, 10.4, 0.8 Hz, 1H), 5.84 (dt, *J* = 10.4, 1.1 Hz, 1H), 5.23 (t, *J* = 5.0 Hz, 1H), 5.17 – 4.90 (m, 4H), 4.27 (dd, *J* = 12.0, 4.4 Hz, 1H), 4.22 – 4.02 (m, 3H), 2.27 (t, *J* = 7.6 Hz, 2H), 1.59 – 1.40 (m, 2H), 1.25 (d, *J* = 5.2 Hz, 24H), 0.88 (t, *J* = 6.7 Hz, 3H).

^{13}C NMR (100 MHz, $CDCl_3$) δ 173.29, 165.11, 135.70, 135.64, 132.04, 128.74, 128.71, 128.69, 128.08, 128.06, 128.00, 127.98, 127.77, 127.65, 69.79, 69.58 (2C), 65.38, 61.60, 34.06, 32.00, 29.77 (2C), 29.76, 29.74 (2C), 29.69, 29.54, 29.44, 29.33, 29.17, 24.90, 22.77, 14.21.

HRMS (ESI) m/z calcd. for $C_{36}H_{53}O_8P$ $[M+H]^+$ 445.3551, found 645.3545.

Synthesis of the compound **1d**:
To a 10 mL dry round-bottom flask, (*R*)-2-(acryloyloxy)-3-((bis(benzyloxy)phosphoryl)oxy)propyl palmitate **S8** (65 mg, 0.1 mmol, 1 equiv) was added in dry CH_2Cl_2 (2 mL). The solution was cooled down to 0 °C, TMSBr (29 µL, 0.22 mmol, 2.2 equiv) was added dropwise under N_2, and the reaction mixture was stirred for 2 h. Upon completion, distilled water (0.5 mL) was added to quench the excess TMSBr. Subsequently, the solvent mixture was

removed by rotary evaporation, and the crude product purified by silica gel column chromatography using $CHCl_3/MeOH/H_2O$ (80/17/3) as the eluent, providing compound **1d** as a colorless oil (38 mg, 81%).

^{1}H NMR (400 MHz, $CDCl_3$) δ 6.41 (d, *J* = 17.3 Hz, 1H), 6.11 (t, *J* = 13.8 Hz, 1H), 5.82 (d, *J* = 10.5 Hz, 1H), 5.31 (s, 1H), 4.31 (d, *J* = 44.9 Hz, 2H), 3.99 (s, 2H), 2.26 (s, 2H), 1.54 (s, 2H), 1.24 (d, *J* = 4.4 Hz, 24H), 0.87 (t, *J* = 6.5 Hz, 3H).

^{13}C NMR (100 MHz, $CDCl_3$) δ 173.93, 166.05, 131.99, 128.19, 71.23, 63.50, 62.99, 34.18, 32.07, 29.90 (5C), 29.83, 29.78, 29.57, 29.52, 29.39, 24.96, 22.83, 14.25.

HRMS (ESI) m/z calcd. for $C_{22}H_{41}O_8P$ $[M+H]^+$ 465.2612, found 465.2619.

*Synthesis of (2S,3R,E)-2-acrylamido-3-hydroxyoctadec-4-en-1-yl (2-(trimethylammonio)ethyl) phosphate (**1e**)*

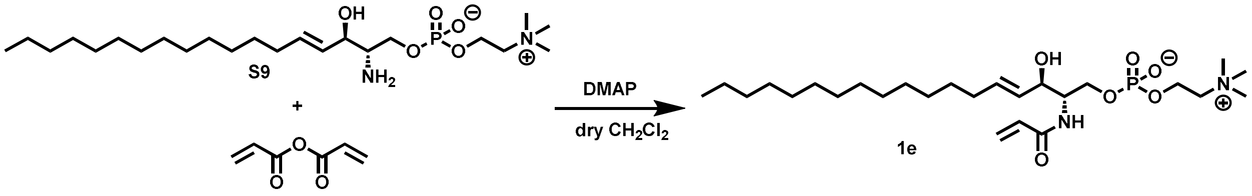

To a 1-dram vial (2*S*,3*R*,4*E*)-2-amino-4-octadecene-3-hydroxy-1-phosphocholine **S9** (23 mg, 0.05 mmol, 1.0 equiv) and DMAP (9.2 mg, 0.05 mmol, 1.0 equiv) were added in 0.5 mL dry CH_2Cl_2. Acetyl anhydride (6 µL, 0.052 mmol, 1.05 equiv) was then added to the mixture, and the reaction was stirred overnight. Subsequently, the mixture was quenched by addition of distilled water (0.5 mL). The solvent was removed under vacuum, and the crude compound purified by silica gel column chromatography using $CHCl_3/MeOH/H_2O$ (50/50/2 – 20/80/2) as the eluent, yielding compound **1e** as a colorless oil (7.5 mg, 43%).

^{1}H NMR (400 MHz, CD_3OD) δ 6.63 – 5.97 (m, 2H), 5.89 – 5.55 (m, 2H), 5.58 – 5.23 (m, 1H), 4.42 – 4.20 (m, 2H), 4.18 – 3.94 (m, 4H), 3.85 – 3.45 (m, 2H), 3.21 (s, 9H), 2.32 – 1.79 (m, 2H), 1.59 – 1.06 (m, 22H), 0.90 (t, 3H).

^{13}C NMR (100 MHz, CD_3OD) δ 167.77, 135.19, 132.29, 131.04, 126.88, 72.73, 65.66, 65.60, 60.46, 55.61, 55.54, 54.70, 54.67, 54.63, 33.39, 33.09, 30.83, 30.81, 30.78, 30.73, 30.68, 30.49, 30.40, 30.26, 23.75, 14.46.

HRMS (ESI) m/z calcd. for $C_{26}H_{51}N_2O_6P$ $[M+H]^+$ 519.3558, found 519.3535.

*Synthesis of N-((2S,3R,E)-1,3-dihydroxyoctadec-4-en-2-yl)acrylamide (**1f**)*

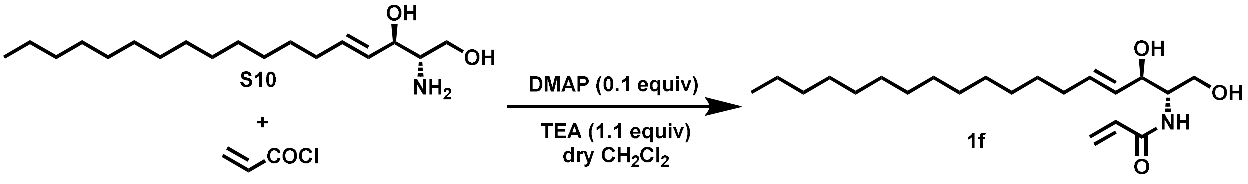

To a dry 10 mL round-bottom flask, sphingosine (d18:1) **S10** (50 mg, 0.167 mmol, 1.0 equiv), triethylamine (26 µL, 0.18 mmol, 1.1 equiv), and DMAP (2.1 mg, 0.0167 mmol, 0.1 equiv) were added in 1 mL dry CH_2Cl_2. Subsequently, acetyl chloride (15 µL, 0.18 mmol, 1.1 equiv) was added, and the mixture was stirred at rt for 2 h. Subsequently, the reaction was quenched by addition of 1 mL of distilled water, the solvent mixture was removed by rotary evaporation, and the crude product purified by silica gel column chromatography using hexane/ethyl acetate (1/3) as the eluent, yielding compound **1f** as a white solid (20 mg, 34%).

^{1}H NMR (400 MHz, $CDCl_3$) δ 6.46 (d, *J* = 7.6 Hz, 1H), 6.32 (dq, *J* = 17.0, 1.4 Hz, 1H), 6.16 (ddd, *J* = 17.0, 10.1, 2.2 Hz, 1H), 5.94 – 5.73 (m, 1H), 5.69 (dq, *J* = 10.2, 1.4 Hz, 1H), 5.55 (ddt, *J* = 15.6, 6.3, 1.5 Hz, 1H), 4.56 – 4.26 (m, 1H), 3.99 (tt, *J* = 6.7, 3.1 Hz, 2H), 3.74 (dt, *J* = 11.3, 3.0 Hz, 1H), 2.06 (q, *J* = 7.2 Hz, 2H), 1.25 (d, *J* = 2.3 Hz, 22H), 0.88 (td, *J* = 6.9, 2.3 Hz, 3H).

^{13}C NMR (100 MHz, $CDCl_3$) δ 166.06, 134.55, 130.70, 128.76, 127.33, 74.84, 62.44, 54.55, 32.42, 32.07, 29.83 (3C), 29.76, 29.63, 29.62, 29.51, 29.34, 29.24, 22.84, 14.27.

HRMS (ESI) m/z calcd. for $C_{21}H_{39}NO_3$ $[M+Na]^+$ 376.2822, found 376.2823.

*Synthesis of (S)-2-(acryloyloxy)-3-hydroxypropyl oleate (**1g**)*

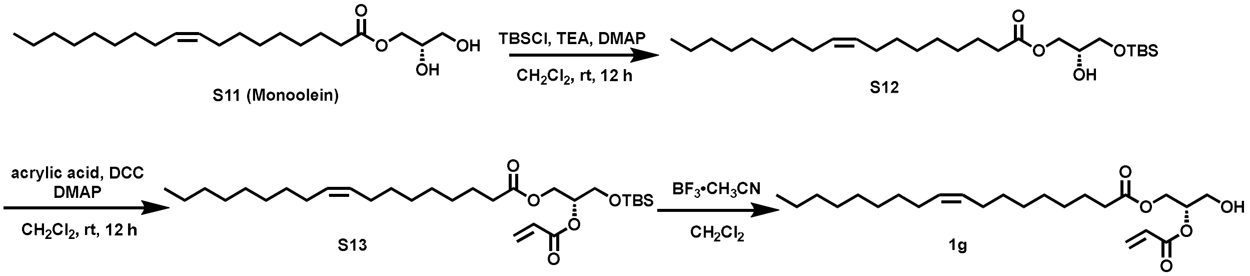

<u>Synthesis of the compound **S12**:</u>
The synthesis was adapted from a previously published procedure[3]. To a 50 mL dry round-bottom flask with a stir bar monoolein **S11** (1.0 g, 2.8 mmol, 1.0 equiv) and DMAP (35 mg, 0.28 mmol, 0.1 equiv) were dissolved in 15 mL of dry CH_2Cl_2. Subsequently, triethyl amine (586 µL, 4.21 mmol, 1.5 equiv) and TBSCl (465 mg, 3.09 mmol, 1.1 equiv) were added to the mixture. After 12 h, distilled water (10 mL) was added, and the aqueous phase was extracted with CH_2Cl_2 (2x). The organic layers were combined, dried over $MgSO_4$, filtered, and concentrated in vacuo. The crude product was further purified by silica flash column chromatography using hexane/ethyl acetate

(20/1) as the eluent. providing **S12** as a colorless oil (1.2 g, 93%). The NMR characterization data of **S12** is consistent with the reported data[3].

^{1}H NMR (400 MHz, $CDCl_3$) δ 5.86 – 5.15 (m, 2H), 4.38 – 3.97 (m, 2H), 3.87 (t, *J* = 5.3 Hz, 1H), 3.79 – 3.47 (m, 2H), 2.52 (s, 1H), 2.33 (t, *J* = 7.6 Hz, 2H), 2.00 (qd, *J* = 6.0, 2.6 Hz, 4H), 1.62 (dd, *J* = 10.7, 4.0 Hz, 2H), 1.46 – 1.15 (m, 20H), 0.88 (d, *J* = 8.9 Hz, 12H), 0.07 (s, 6H).

^{13}C NMR (100 MHz, $CDCl_3$) δ 174.09, 130.14, 129.87, 70.11, 65.12, 63.80, 34.30, 32.04, 29.89, 29.82, 29.65, 29.45, 29.30, 29.24, 29.23, 27.34, 27.29, 25.96, 25.05, 22.82, 18.39, 14.25, -5.33.

<u>Synthesis of the compound **S13**:</u>
To a 50 mL dry round-bottom flask, **S12** (708 mg, 1.5 mmol, 1.0 equiv), acrylic acid (309 μL, 4.5 mmol, 3.0 equiv), DMAP (24 mg, 0.15 mmol, 0.1 equiv), and DCC (930 mg, 4.5 mmol, 3.0 equiv) were added in 10 mL of dry CH_2Cl_2. The mixture was stirred at rt overnight. The resulting mixture was filtered and purified by silica gel column chromatography using hexane/ethyl acetate (20/1) as the eluent, providing **S13** as a colorless oil (498 mg, 63%).

^{1}H NMR (400 MHz, $CDCl_3$) δ 6.42 (dd, *J* = 17.3, 1.4 Hz, 1H), 6.12 (dd, *J* = 17.3, 10.4 Hz, 1H), 5.85 (dd, *J* = 10.4, 1.5 Hz, 1H), 5.50 – 5.24 (m, 2H), 5.14 (dtd, *J* = 6.4, 5.2, 3.7 Hz, 1H), 4.35 (dd, *J* = 11.9, 3.7 Hz, 1H), 4.23 (dd, *J* = 11.9, 6.4 Hz, 1H), 3.76 (dd, *J* = 5.2, 1.3 Hz, 2H), 2.30 (t, *J* = 7.6 Hz, 2H), 2.07 – 1.84 (m, 4H), 1.61 – 1.45 (m, 2H), 1.56 – 1.03 (m, 20H), 0.87 (s, 12H), 0.04 (s, 6H).

^{13}C NMR (100 MHz, $CDCl_3$) δ 173.63, 165.55, 131.44, 130.15, 129.88, 128.37, 72.25, 62.56, 61.56, 35.07, 34.29, 32.05, 29.91, 29.85, 29.66, 29.46, 29.32, 29.25, 29.22, 27.36, 27.31, 25.89, 25.59, 25.04, 24.84, 22.82, 18.34, 14.26, -5.33.

HRMS (ESI) m/z calcd. for $C_{30}H_{56}O_5Si$ $[M+H]^+$ 525.3970, found 525.3979.

<u>Synthesis of the compound **1g**:</u>
To a 25 mL dry round-bottom flask, **S13** (0.3 g, 0.57 mmol, 1.0 equiv) was added in 5 mL dry CH_2Cl_2. The solution was cooled to 0 °C, $BF_3•CH_3CN$ (145 μL, 1.14 mmol, 2.0 equiv) was added dropwise, and the mixture was stirred for 30 min at 0 °C and monitored by TLC. If starting materials were still present, another addition of $BF_3•CH_3CN$ (145 μL) was performed before work-up. Upon completion, PBS buffer (chilled in an ice bath, 5 mL) was added to the mixture, and the aqueous phase was extracted with EtOAc (3x). The organic layers were combined, dried over $MgSO_4$, filtered, and concentrated in vacuo. The crude product was further purified by silica flash column chromatography using hexane/ethyl acetate (5/1) as the eluent, providing **1g** as a colorless oil (190 mg, 81%).

^{1}H NMR (400 MHz, $CDCl_3$) δ 6.44 (dd, *J* = 17.3, 1.4 Hz, 1H), 6.13 (dd, *J* = 17.3, 10.4 Hz, 1H), 5.87 (dd, *J* = 10.4, 1.4 Hz, 1H), 5.45 – 5.21 (m, 2H), 5.15 (dq, *J* = 5.8, 4.9 Hz, 1H), 4.31 (qd, *J* = 12.0, 5.1 Hz, 2H), 3.75 (d, *J* = 5.0 Hz, 2H), 2.71 – 2.37 (m, 1H), 2.31 (t, *J* = 7.5 Hz, 2H), 2.13 – 1.79 (m, 4H), 1.58 (q, *J* = 7.2 Hz, 2H), 1.27 (dd, *J* = 11.8, 8.2 Hz, 20H), 1.01 – 0.53 (m, 3H).

^{13}**C NMR** (100 MHz, $CDCl_3$) δ 173.9, 165.8, 132.0, 130.1, 129.8, 128.0, 72.6, 62.1, 61.4, 34.2, 32.0, 29.9, 29.8, 29.6, 29.4 (2C), 29.2, 29.2, 29.2, 27.3, 27.2, 24.9, 22.8, 14.2.

HRMS (ESI) m/z calcd. for $C_{24}H_{42}O_5$ $[M+H]^+$ 411.3105, found 411.3106.

General procedure for the synthesis of NHPI esters 2a-2e:

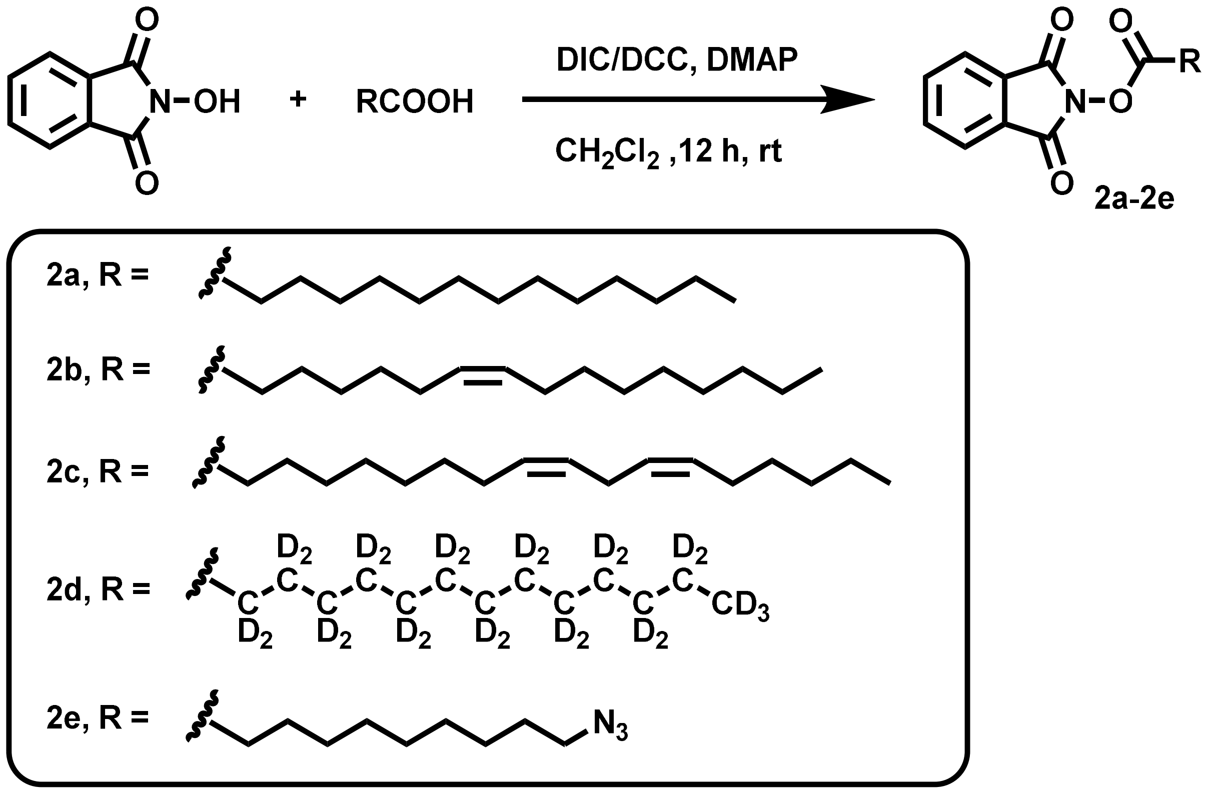

To a 25 mL dry round-bottom flask, *N*-hydroxyphthalimide (5.5 mmol, 1.1 equiv), carboxylic acid (5 mmol, 1.0 equiv), 4-dimethylaminopyridine (DMAP, 0.5 mmol, 0.1 equiv), and N,N'-diisopropylcarbodiimide (DIC) or N,N'-dicyclohexylcarbodiimide (DCC, 5.5 mmol, 1.1 equiv) were added in 15 mL of dry CH_2Cl_2. The reaction mixture was stirred at room temperature overnight. After completion, the mixture was filtered, and the solvent removed under vacuum. The crude compound was then purified by column chromatography using hexane/ethyl acetate (50/1) as the eluent.

*Synthesis of 1,3-dioxoisoindolin-2-yl tetradecanoate (**2a**)*

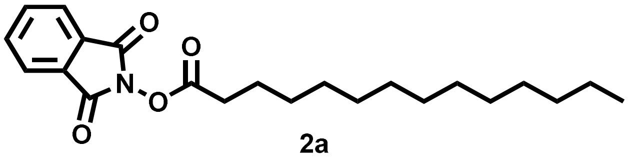

2a

According to the general procedure, compound **2a** (1.35 g, 83%) was obtained as a white solid using myristic acid (1.0 g, 4.38 mmol, 1.0 equiv), *N*-hydroxyphthalimide (0.786 g, 4.82 mmol, 1.1 equiv), DCC (0.994, 4.82 mmol, 1 equiv), and DMAP (0.0534 g, 0.438 mmol, 0.1 equiv).

^{1}H NMR (400 MHz, $CDCl_3$) δ 7.96 – 7.84 (m, 2H), 7.83 – 7.73 (m, 2H), 2.66 (t, *J* = 7.5 Hz, 2H), 1.78 (p, *J* = 7.5 Hz, 2H), 1.27 (d, *J* = 6.5 Hz, 20H), 0.88 (t, *J* = 6.7 Hz, 3H).

^{13}C NMR (100 MHz, $CDCl_3$) δ 169.82, 162.17, 134.87, 129.11, 124.10, 32.07, 31.14, 29.81, 29.79, 29.77, 29.71, 29.52, 29.50, 29.26, 28.98, 24.82, 22.84, 14.27.

HRMS (ESI) m/z calcd. for $C_{22}H_{31}NO_4$ $[M+MeOH+Na]^+$ 428.2407, found 428.2402.

*Synthesis of 1,3-dioxoisoindolin-2-yl (Z)-hexadec-7-enoate (**2b**)*

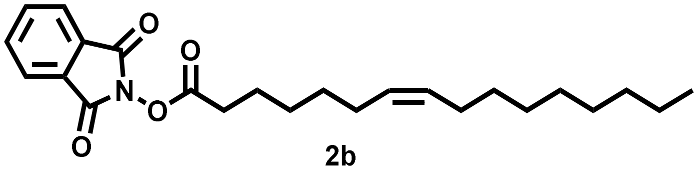

2b

According to the general procedure, compound **2b** (74.4 mg, 95%) was obtained as a colorless oil using *cis*-7-hexadecenoic acid (50 mg, 0.197 mmol, 1.0 equiv), *N*-hydroxyphthalimide (36 mg, 0.216 mmol, 1.1 equiv), DIC (34 μL, 0.216 mmol, 1 equiv), and DMAP (2.4 mg, 0.022 mmol, 0.1 equiv).

^{1}H NMR (400 MHz, $CDCl_3$) δ 8.34 – 7.83 (m, 2H), 7.81 – 7.54 (m, 2H), 5.35 (p, *J* = 5.3 Hz, 2H), 2.66 (t, *J* = 7.5 Hz, 2H), 2.03 (dq, *J* = 16.7, 6.6 Hz, 4H), 1.79 (p, *J* = 7.4 Hz, 2H), 1.58 – 1.07 (m, 16H), 0.87 (t, 3H).

^{13}C NMR (100 MHz, $CDCl_3$) δ 169.71, 162.10, 134.84, 130.49, 129.37, 129.05, 124.05, 32.01, 31.06, 29.86, 29.64, 29.45, 29.43, 29.34, 28.59, 27.36, 27.03, 24.70, 22.79, 14.23.

HRMS (ESI) m/z calcd. for $C_{24}H_{33}NO_4$ $[M+MeOH+Na]^+$ 454.2564, found 454.2562.

*1,3-dioxoisoindolin-2-yl (9Z,12Z)-octadeca-9,12-dienoate (**2c**)*

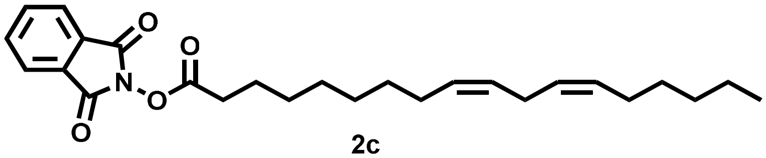

2c

According to the general procedure, compound **2c** (280 mg, 84%) was obtained as a colorless oil using linoleic acid (250 mg, 0.891 mmol, 1 equiv), *N*-hydroxyphthalimide (160 mg, 0.98 mmol, 1.1 equiv), DCC (203 mg, 0.98 mmol, 1.1 equiv), DMAP (11 mg, 0.089 mmol, 0.1 equiv).

^{1}H NMR (400 MHz, $CDCl_3$) δ 7.89 (dd, *J* = 5.5, 3.2 Hz, 2H), 7.79 (dd, *J* = 5.5, 3.1 Hz, 2H), 5.62 – 4.98 (m, 4H), 2.78 (t, *J* = 6.6 Hz, 2H), 2.66 (t, *J* = 7.5 Hz, 2H), 2.26 – 1.94 (m, 4H), 1.78 (p, *J* = 7.5 Hz, 2H), 1.50 – 1.17 (m, 14H), 0.88 (t, 3H).

^{13}C NMR (100 MHz, $CDCl_3$) δ 169.78, 162.16, 134.87, 130.37, 130.17, 129.07, 128.21, 128.05, 124.10, 31.65, 31.11, 29.69, 29.48, 29.16, 28.93, 27.33, 27.31, 25.76, 24.78, 22.72, 14.22.

HRMS (ESI) m/z calcd. for $C_{26}H_{35}NO_4$ $[M+MeOH+H]^+$ 458.2901, found 458.2899.

*Synthesis of 1,3-dioxoisoindolin-2-yl tetradecanoate-d$_{27}$ (**2d**)*

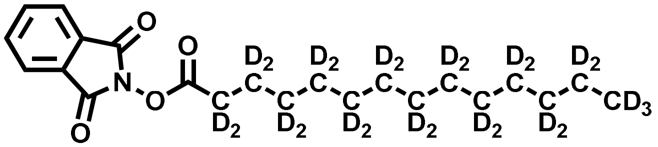

2d

According to the general procedure, compound **2d** (0.21 g, 61%) was obtained as a white solid using using myristic acid-d$_{27}$ (0.2 g, 0.783 mmol, 1.0 equiv), DCC (0.18 g, 0.86 mmol, 1.1 equiv), *N*-hydroxyphthalimide (0.14 mg, 0.86 mmol, 1.1 equiv), and DMAP (0.01 g, 0.0783 mmol, 0.1 equiv).

^{1}H NMR (400 MHz, $CDCl_3$) δ 7.86 (dt, *J* = 6.2, 3.2 Hz, 2H), 7.77 (dt, *J* = 6.0, 3.2 Hz, 2H).

^{13}C NMR (100 MHz, $CDCl_3$) δ 169.77, 162.07, 134.81, 129.03, 124.00.

HRMS (ESI) m/z calcd. for $C_{22}H_4D_{27}NO_4$ $[M+MeOH+Na]^+$ 455.4102, found 455.4102.

*Synthesis of 1,3-dioxoisoindolin-2-yl 10-azidodecanoate (**2e**)*

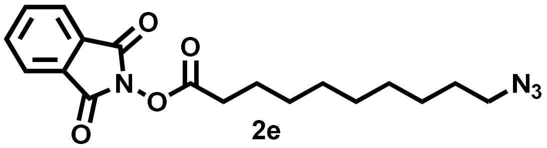

According to the general procedure, compound **2e** (126 mg, 75%) was obtained as a colorless oil using 10-azidodecanoic acid (0.1 g, 0.469 mmol, 1.0 equiv), DCC (0.106 g, 0.516 mmol, 1.1 equiv), *N*-hydroxyphthalimide (0.084 mg, 0.516 mmol, 1.1 equiv), and DMAP (5.73 mg, 0.047 mmol, 0.1 equiv).

^{1}H NMR (400 MHz, $CDCl_3$) δ 7.93 – 7.82 (m, 2H), 7.82 – 7.71 (m, 2H), 3.24 (t, *J* = 7.0 Hz, 2H), 2.65 (t, *J* = 7.4 Hz, 2H), 1.77 (p, *J* = 7.4 Hz, 2H), 1.65 – 1.51 (m, 2H), 1.47 – 1.28 (m, 10H).

^{13}C NMR (100 MHz, $CDCl_3$) δ 169.71, 162.08, 134.82, 128.99, 124.02, 51.53, 31.04, 29.24, 29.07, 29.03, 28.89, 28.78, 26.72, 24.71.

HRMS (ESI) m/z calcd. for $C_{18}H_{22}N_4O_4$ $[M+Na]^+$ 381.1533, found 381.1535.

3. Optimization of photoredox lipid ligation (PLL)

Supplementary Table 1. Synthesis of phospholipid **3a** by oxidative decarboxylation of myristic acid **S14** under blue light.

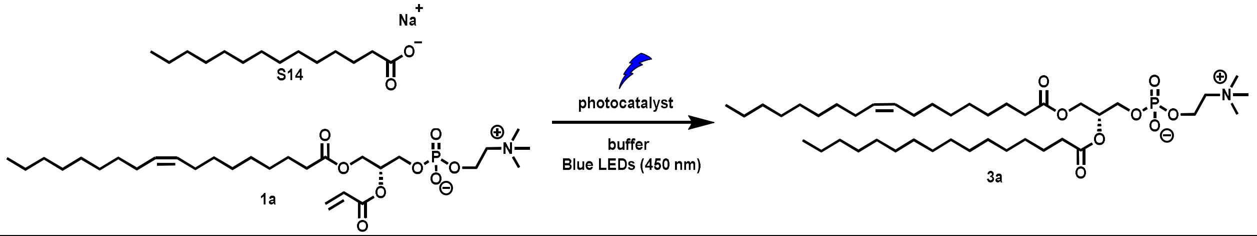

Entry	Conditions	Yield 3a (%)
1	**S14** (2 mM), **1a** (1 mM), Riboflavin (0.1 mM), PBS (pH =7.4), Blue LEDs, 12 h	trace
2	**S14** (2 mM), **1a** (1 mM), Lumiflavin (0.1 mM), PBS (pH=7.4), Blue LEDs, 12 h	trace
3	**S14** (2 mM), **1a** (1 mM), Ir[dF(CF_3)$_2$ppy](dtbpy) (0.1 mM), PBS (pH=7.4), Blue LEDs, 12 h	trace
4	**S14** (2 mM), **1a** (1 mM), Riboflavin (0.1 mM), Na_2CO_3/$NaHCO_3$ (pH=10), Blue LEDs, 12 h	trace

Supplementary Table 2. Yield of OPPC **3a** lipid under green light irradiation using various photocatalysts.

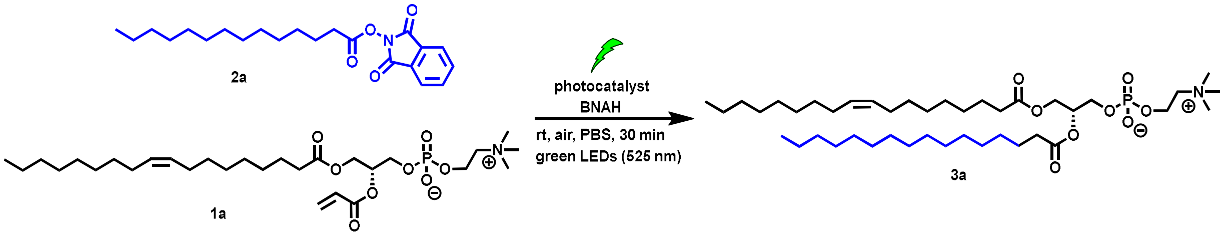

Entry	Conditions	Yield 3a (%)[a]
1	**2a** (1.5 mM), **1a** (1 mM), BNAH (3 mM), eosin Y (0.05 mM)	48
2	**2a** (1 mM), **1a** (2 mM), BNAH (3 mM), Dextran-RhodB (1 mg/mL)	91
3	**2a** (1 mM), **1a** (2 mM), BNAH (3 mM), Dextran-Texas Red (1 mg/mL)	50
4	**2a** (1 mM), **1a** (2 mM), BNAH (3 mM), Nile Red (0.05 mM)	trace
5	**2a** (1 mM), **1a** (2 mM), BNAH (3 mM), Phenol red[b]	trace
6	**2a** (1 mM), **1a** (2 mM), eosin Y (0.05 mM)	trace

Note: [a]HPLC-ELSD yield. [b]DMEM medium with phenol red inside.

Supplementary Table 3. Synthesis of **3a** by reductive decarboxylation of **2a** under blue light.

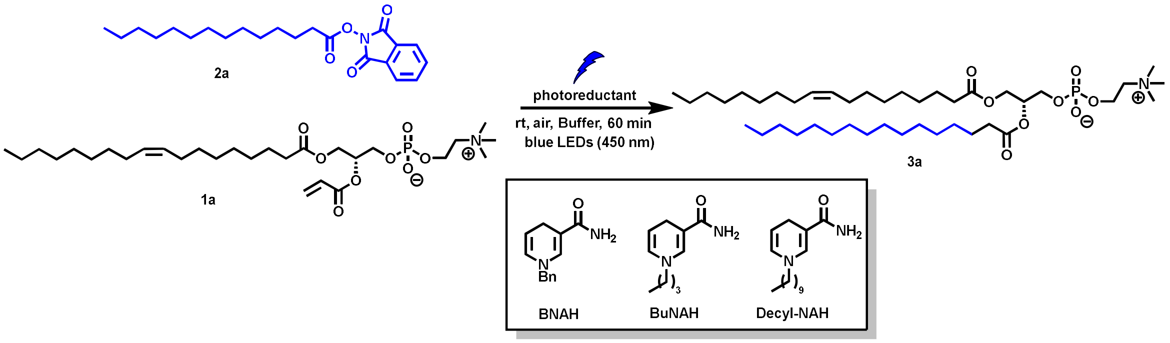

Entry	Conditions	Yield 3a (%)[a]
1	**2a** (1 mM), **1a** (1.5 mM), BuNAH (3 mM), H_2O	28
2	**2a** (1 mM), **1a** (1.5 mM), BNAH (3 mM), H_2O	55
3	**2a** (1 mM), **1a** (1.5 mM), Decyl-NAH (3 mM), H_2O	47
4	**2a** (1 mM), **1a** (2 mM), BNAH (3 mM), PBS	77
5	Same to entry 4 but no blue light	trace
6	Same to entry 4 but no BNAH	trace

Note: [a]HPLC-ELSD yield.

Supplementary Table 4. Synthesis of POPC in the presence of palmitic acid or cholesterol.

Reaction conditions	Standard condition	Standard condition + 1 mM palmitic acid	Standard condition + 1 mM cholesterol
**HPLC Yield of POPC (%)**	89	92	90

4. Synthesis of different lipids by PLL

General procedure for the synthesis of phospholipids 3a-3e, 3g, 3j-3k

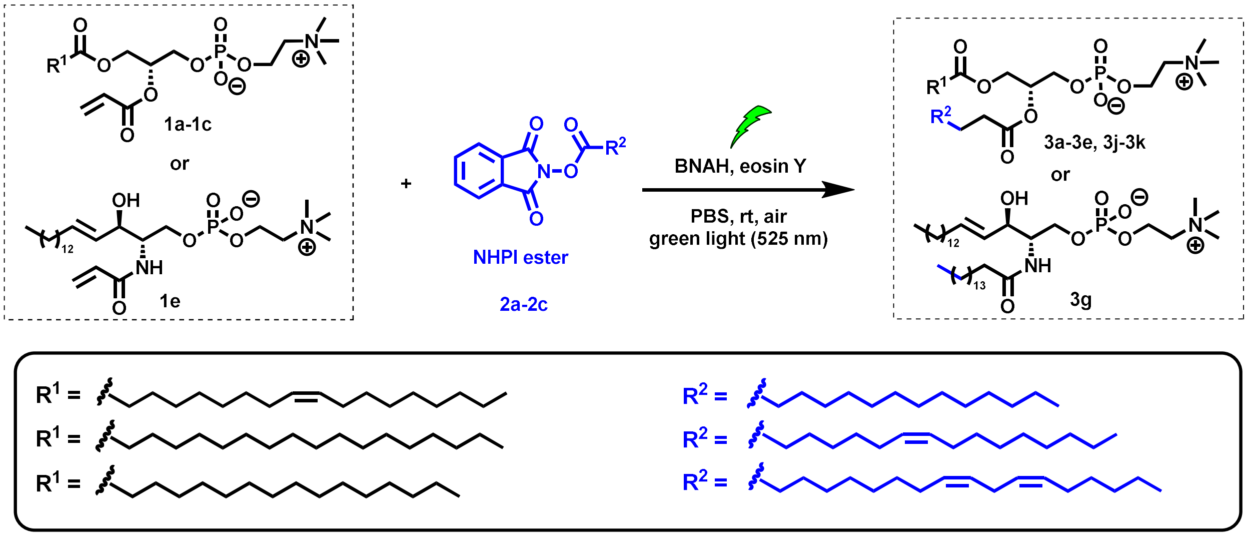

To a 1-dram glass vial, 100 µL of a 10 mM stock solution of compound **1a-1c** or **1e** (2.0 equiv) in $CHCl_3$, 50 µL of a 10 mM solution of compound **2a-2c** (1 equiv) in $CHCl_3$, 150 µL of a 10 mM BNAH (3 .0 equiv) in $CHCl_3$, and 2.5 µL of a 10 mM eosin Y (0.05 equiv) in MeOH were added. The solvent was evaporated under a gentle stream of N_2, and the content was redissolved with 300 µL of CH_2Cl_2. The CH_2Cl_2 was evaporated again, while carefully rotating the vial to obtain a thin lipid film. The film was hydrated with 500 µL of phosphate buffered saline (PBS, 1x, Cytiva, Cat. No. SH30256.01, Lot No. AJ30751067) and the suspension was sonicated for 1-2 min, until a red homogenous lipid dispersion was formed. The sample was irradiated with a 18 W green LEDs

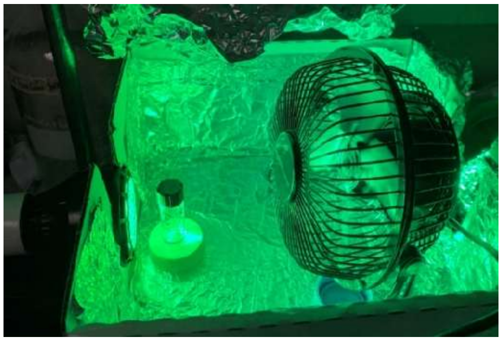

(λ_{max} 525 nm) positioned at approximately 1 cm distance, while being cooled by an electronic fan (reaction setup shown to the left) to a temperature of about 20 °C. After 15-30 min, the reaction was analyzed by HPLC-ELSD-MS, showing the 100% conversion of **2a-2c**. The crude mixture was purified by silica gel column chromatography using $MeOH/CHCl_3/H_2O$ (50/50/2–80/20/2) as eluents, to provide the phospholipid product.

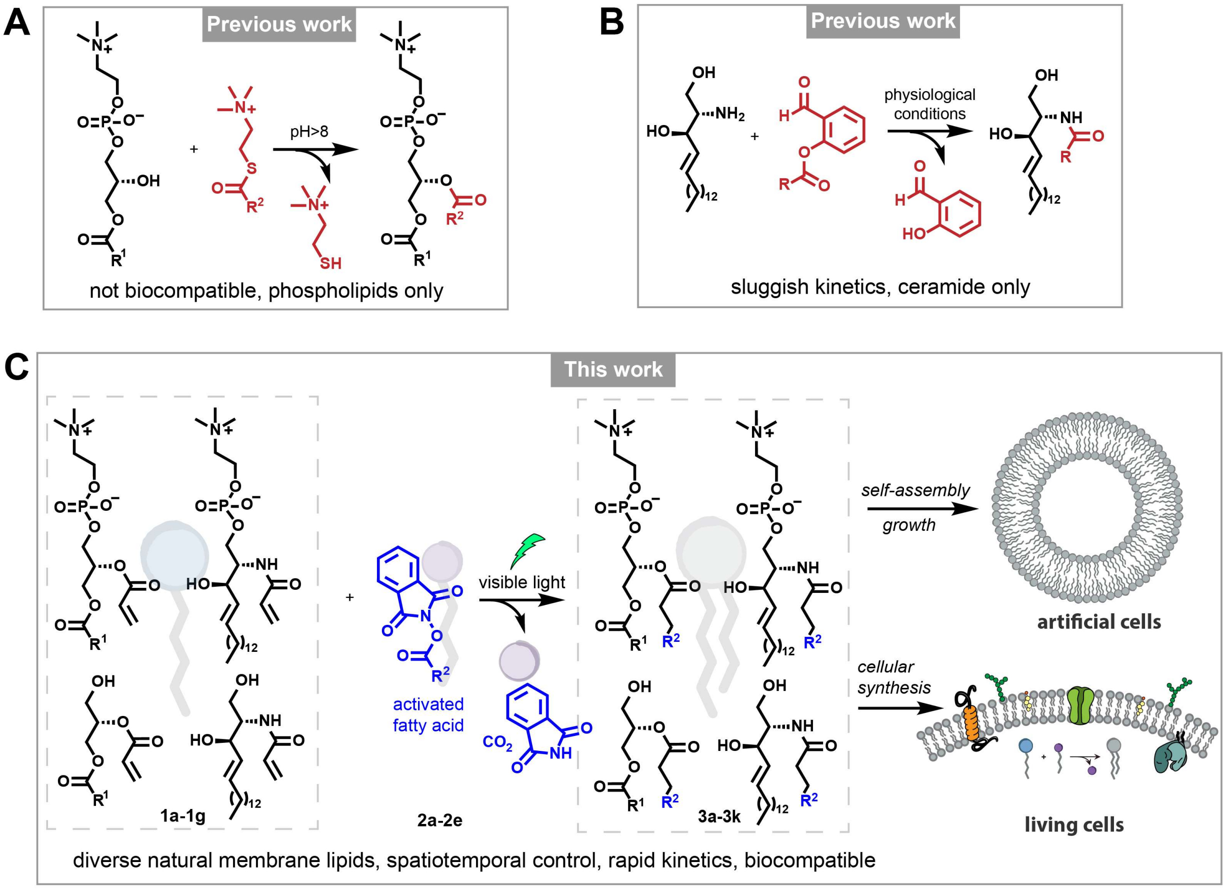

Supplementary Fig. 1. Abiotic synthesis of natural membrane lipids in water via photoredox lipid ligation (PLL). (A) Enzyme-free synthesis of natural phospholipids under prebiotically relevant alkaline conditions. (B) Traceless synthesis of ceramide by acylation of sphingosine in living cells. (C) Unified strategy for the synthesis of a wide range of natural lipids in artificial and living cells through PLL (this work).

General procedure for the synthesis of ceramides 3h and diacylglycerols 3i:

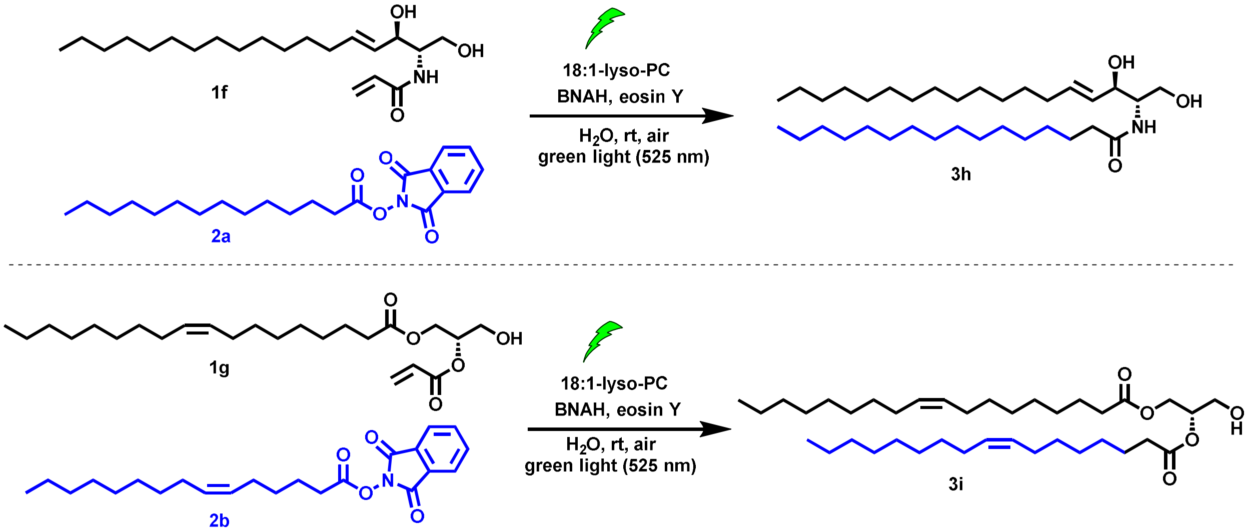

To a 1-dram vial 50 µL of a 10 mM stock solution of compounds **1f** or **1g** (2.0 equiv) in $CHCl_3$, 100 µL of a 10 mM solution of compound **2a** or **2b** (1.0 equiv) in $CHCl_3$, 150 µL of a 10 mM BNAH (3.0 equiv) solution in $CHCl_3$, 150 µL of a 10 mM stock solution of 18:1-lyso-PC (3.0 equiv) in $CHCl_3$, and 2.5 µL of a 10 mM eosin Y (0.05 equiv) in MeOH were added. The solvent was evaporated under a gentle stream of N_2, and the content was redissolved with 300 µL of CH_2Cl_2. The CH_2Cl_2 was evaporated again, while carefully rotating the vial to obtain a thin lipid film. The film was hydrated with 500 µL of distill water and the suspension was sonicated for 1-2 min, until a red homogenous lipid dispersion was formed. The sample was irradiated with an 18 W green LEDs (λ_{max} 525 nm) positioned at approximately 1 cm distance, while being cooled by an electronic fan (reaction setup shown in Figure S1). After 30 min, the reaction was checked by HPLC-ELSD-MS, which indicated consumption of **2a** or **2b**. The crude mixture was purified by silica gel column chromatography using a mixture of hexane/ethyl acetate as eluent, to provide the neutral lipid product.

Procedure for the synthesis of phosphatidic acid 3f:

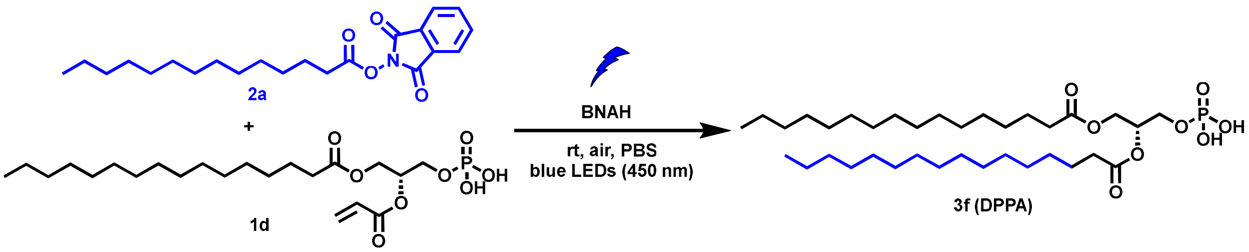

To a 1-dram vial 100 µL of a 10 mM stock solution of **1d** (2.0 equiv) in $CHCl_3$, 50 µL of a 10 mM stock solution of **2a** (1.0 equiv) in $CHCl_3$, and a 150 µL of a 10 mM BNAH (3.0 equiv) solution in $CHCl_3$ were added. The solvent was evaporated under a gentle stream of N_2, and the content

was redissolved with 300 μL of CH_2Cl_2. The CH_2Cl_2 was evaporated again, while carefully rotating the vial to obtain a thin lipid film. The film was hydrated with 500 μL of PBS, and the suspension was sonicated for 1-2 min, until a homogenous lipid dispersion was formed. The sample was irradiated with Blue LEDs (Kessil A160WE Tuna Blue LED Light) positioned at approximately 1 cm distance, while being cooled by an electronic fan. After 30 min, the reaction was checked by HPLC-ELSD-MS, which indicated consumption of **2a**. A calibration curve for DPPA concentration was made by determining the area of the ELSD signal (representative HPLC-ELSD trace shown below) versus DPPA concentration. The HPLC-ELSD yield of this reaction is 38%, which is estimated by comparison with the DPPA standard curve.

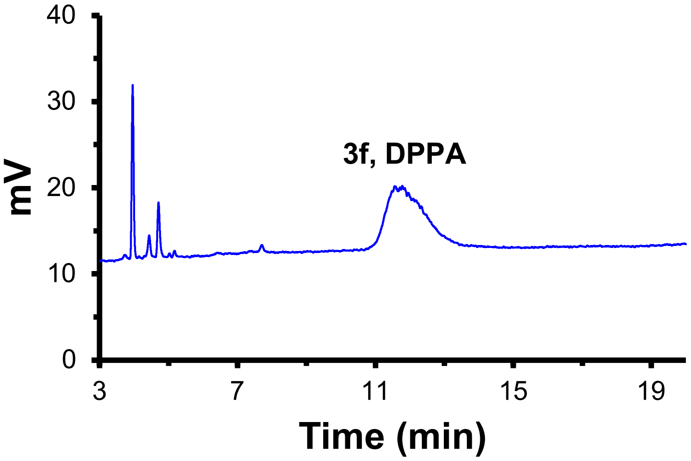

*(R)-3-(oleoyloxy)-2-(palmitoyloxy)propyl (2-(trimethylammonio)ethyl) phosphate (**3a**)*

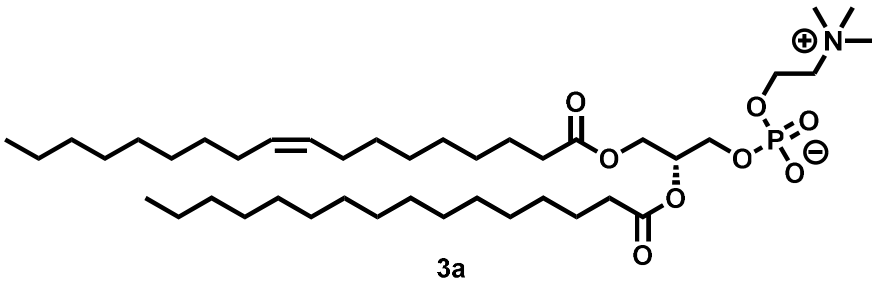

3a

The colorless oil **3a** (OPPC) was synthesized according to the general procedure. An isolated yield of 95% was obtained using $MeOH/CHCl_3/H_2O$ (50/50/2–80/20/2) as the eluents. The NMR data is identical to the reported literature[4].

^{1}H NMR (400 MHz, $CDCl_3$) δ 5.42 – 5.27 (m, 2H), 5.19 (q, J = 2.9 Hz, 1H), 4.37 (dd, J = 12.0, 2.7 Hz, 1H), 4.27 (d, J = 7.6 Hz, 2H), 4.11 (dd, J = 12.1, 7.5 Hz, 1H), 3.92 (q, J = 6.1 Hz, 2H), 3.72 (d, J = 5.2 Hz, 2H), 3.30 (s, 9H), 2.28 (dt, J = 11.1, 7.6 Hz, 4H), 2.00 (q, J = 6.4 Hz, 4H), 1.57 (s, 4H), 1.39 – 1.07 (m, 48H), 0.87 (t, J = 6.6 Hz, 6H).

HRMS m/z (ESI): calcd. for $C_{42}H_{82}NO_8P$ $[M+H]^+$: 760.5851; found: 760.5848.

*(R)-2-(oleoyloxy)-3-(palmitoyloxy)propyl (2-(trimethylammonio)ethyl) phosphate (**3b**)*

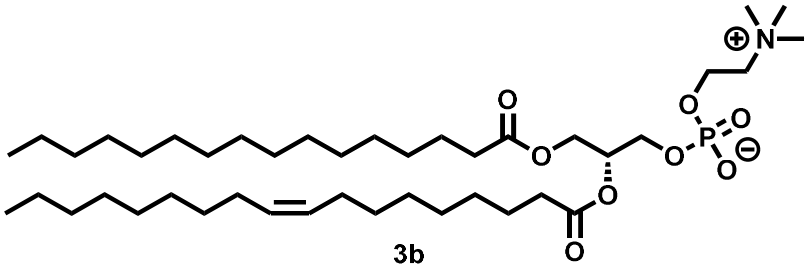

3b

The colorless oil **3b** (POPC) was synthesized according to the general procedure. An isolated yield of 92% was obtained using $MeOH/CHCl_3/H_2O$ (50/50/2–80/20/2) as the eluents. The NMR data is consistent with the reported literature[4].

^{1}H NMR (400 MHz, $CDCl_3$) δ 5.38 – 5.28 (m, 2H), 5.23 – 5.13 (m, 1H), 4.38 (dd, *J* = 12.0, 2.8 Hz, 1H), 4.28 (d, *J* = 7.7 Hz, 2H), 4.09 (td, *J* = 12.4, 6.5 Hz, 1H), 3.91 (tq, *J* = 11.1, 5.3 Hz, 2H), 3.76 (d, *J* = 5.2 Hz, 2H), 3.33 (s, 9H), 2.27 (q, *J* = 7.8 Hz, 4H), 2.00 (d, *J* = 6.2 Hz, 4H), 1.57 (q, *J* = 6.4 Hz, 4H), 1.38 – 1.22 (m, 48H), 0.87 (t, *J* = 6.6 Hz, 6H).

HRMS m/z (ESI): calcd. for $C_{42}H_{82}NO_8P$ $[M+H]^+$: 760.5851; found: 760.5846.

*(R)-2,3-bis(oleoyloxy)propyl (2-(trimethylammonio)ethyl) phosphate (**3c**)*

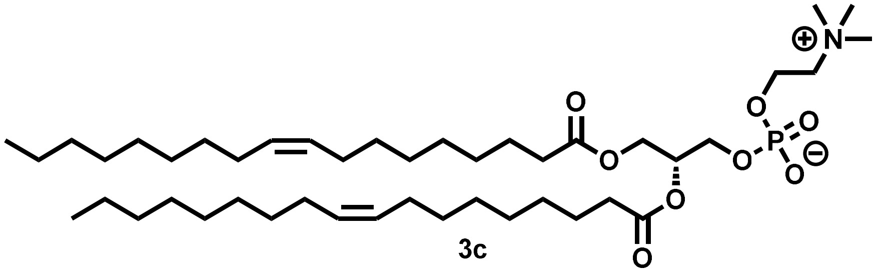

3c

The colorless oil **3c** (DOPC) was synthesized according to the general procedure. An isolated yield of 91% was obtained using $MeOH/CHCl_3/H_2O$ (50/50/2–80/20/2) as the eluents. The NMR data is consistent with the reported literature[4].

^{1}H NMR (400 MHz, $CDCl_3$) δ 5.37 – 5.29 (m, 4H), 5.19 (q, *J* = 6.9 Hz, 1H), 4.51 – 4.24 (m, 3H), 4.10 (dd, *J* = 12.1, 7.5 Hz, 1H), 3.93 (t, *J* = 6.6 Hz, 2H), 3.88 – 3.76 (m, 2H), 3.35 (s, 9H), 2.28 (dt, *J* = 11.4, 7.6 Hz, 4H), 2.00 (q, *J* = 6.3 Hz, 8H), 1.66 – 1.44 (m, 4H), 1.29 (dq, *J* = 12.4, 6.6 Hz, 40H), 0.87 (t, *J* = 6.7 Hz, 6H).

HRMS m/z (ESI): calcd. for $C_{44}H_{84}NO_8P$ $[M+H]^+$: 786.6007; found: 786.6010.

*(R)-2-(((11Z,14Z)-icosa-11,14-dienoyl)oxy)-3-(oleoyloxy)propyl (2-(trimethylammonio)ethyl) phosphate (**3d**)*

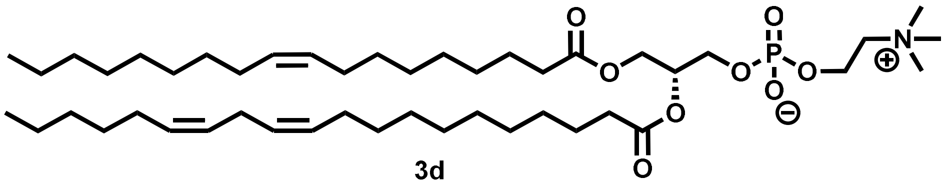

3d

The colorless oil product **3d** was synthesized according to the general procedure. An isolated yield of 84% was obtained using MeOH/$CHCl_3$/H_2O (50/50/2–80/20/2) as the eluents.

^{1}H NMR (400 MHz, $CDCl_3$) δ 5.44 – 5.26 (m, 6H), 5.24 – 5.14 (m, 1H), 4.48 – 4.23 (m, 3H), 4.25 – 4.04 (m, 1H), 3.95 (s, 2H), 3.79 (s, 2H), 3.33 (s, 9H), 2.77 (t, *J* = 6.5 Hz, 2H), 2.28 (q, *J* = 7.9 Hz, 4H), 2.02 (dq, *J* = 13.0, 6.6 Hz, 8H), 1.57 (s, 4H), 1.50 – 1.13 (m, 38H), 1.02 – 0.70 (m, 6H).

^{13}C NMR (100 MHz, $CDCl_3$) δ 173.75, 173.46, 130.33, 130.21, 130.13, 129.80, 128.11, 128.05, 70.50, 66.41, 63.80, 63.03, 59.65, 54.51, 34.43, 34.24, 32.05, 31.66, 29.93, 29.91, 29.89, 29.87, 29.84, 29.80, 29.75, 29.68, 29.66, 29.61, 29.55, 29.51, 29.49, 29.47, 29.39, 29.34, 27.40, 27.36, 27.32, 25.76, 25.12, 25.02, 22.83, 22.72, 14.27, 14.23.

HRMS m/z (ESI): calcd. for $C_{46}H_{86}NO_8P$ $[M+H]^+$: 812.6164; found: 812.6175.

*(R)-2,3-bis(palmitoyloxy)propyl (2-(trimethylammonio)ethyl) phosphate (**3e**)*

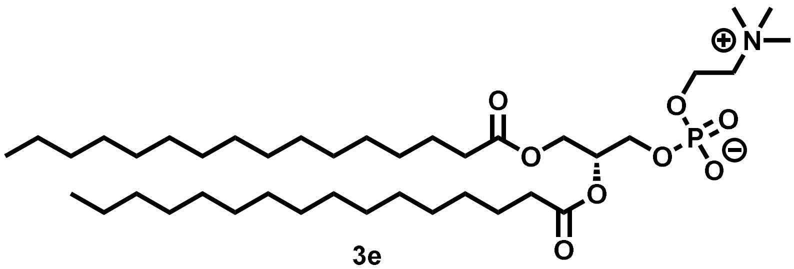

3e

The white solid **3e** (DPPC) was synthesized according to the general procedure. An isolated yield of 82% was obtained using MeOH/$CHCl_3$/H_2O (50/50/2–80/20/2) as the eluents. The NMR data is consistent with the reported literature[4].

^{1}H NMR (400 MHz, $CDCl_3$) δ 5.19 (s, 1H), 4.38 (d, *J* = 11.8 Hz, 1H), 4.30 (s, 2H), 4.11 (dd, *J* = 12.0, 7.4 Hz, 1H), 3.92 (s, 2H), 3.78 (s, 2H), 3.33 (s, 9H), 2.28 (dt, *J* = 10.2, 7.6 Hz, 4H), 1.57 (s, 4H), 1.25 (s, 48H), 0.87 (t, *J* = 6.7 Hz, 6H).

HRMS m/z (ESI): calcd. for $C_{40}H_{80}NO_8P$ $[M+H]^+$: 734.5694; found: 734.5700.

(2S,3R,E)-3-hydroxy-2-palmitamidooctadec-4-en-1-yl (2-(trimethylammonio)ethyl) phosphate ***(3g)***

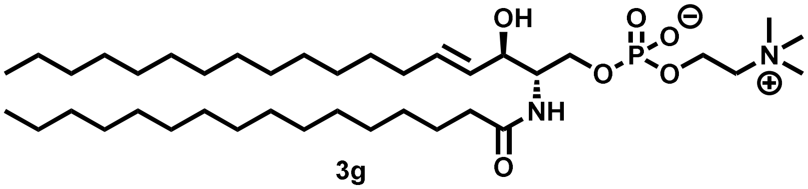

3g

The white solid was synthesized according to the general procedure. An isolated yield of 84% was obtained using MeOH/$CHCl_3$/H_2O (50/50/2–80/20/2) as the eluents. The NMR data is consistent with the reported literature[5].

^{1}H NMR (400 MHz, CD_3OD) δ 5.67 (dt, *J* = 14.2, 6.6 Hz, 1H), 5.41 (dd, *J* = 15.2, 7.6 Hz, 1H), 4.25 (s, 2H), 4.18 – 3.81 (m, 4H), 3.77 – 3.57 (m, 2H), 3.19 (d, *J* = 2.4 Hz, 9H), 2.34 – 2.08 (m, 2H), 1.99 (d, *J* = 7.3 Hz, 2H), 1.54 (s, 2H), 1.26 (d, *J* = 5.1 Hz, 46H), 1.14 – 0.83 (m, 6H).

HRMS m/z (ESI): calcd. for $C_{39}H_{79}N_2O_6P$ $[M+H]^+$: 703.5749; found: 703.5754.

N-((2S,3R,E)-1,3-dihydroxyoctadec-4-en-2-yl)palmitamide ***(3h)***

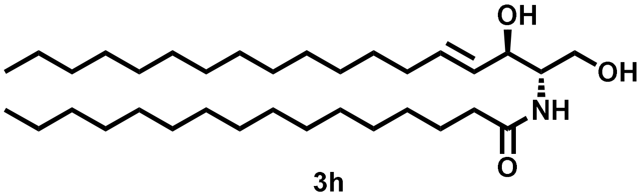

3h

The white solid **3h** was synthesized according to the general procedure. An isolated yield of 70% was obtained using hexane/ethyl acetate (1/1–1/2) as the eluents. The NMR data is consistent with the reported literature[6].

^{1}H NMR (400 MHz, $CDCl_3$) δ 6.33 (d, *J* = 7.5 Hz, 1H), 5.77 (dtd, *J* = 15.0, 6.8, 1.3 Hz, 1H), 5.51 (ddt, *J* = 15.4, 6.4, 1.5 Hz, 1H), 4.46 – 4.22 (m, 1H), 4.11 – 3.79 (m, 2H), 3.68 (dd, *J* = 11.1, 3.3 Hz, 1H), 2.32 – 2.16 (m, 2H), 2.04 (q, *J* = 7.1 Hz, 2H), 1.61 (q, *J* = 7.3 Hz, 2H), 1.45 – 1.13 (m, 46H), 0.87 (t, 6H).

HRMS m/z (ESI): calcd. for $C_{34}H_{67}NO_3$ $[M+H]^+$: 538.5194; found: 538.5192.

*(S)-3-hydroxypropane-1,2-diyl dioleate (**3i**)*

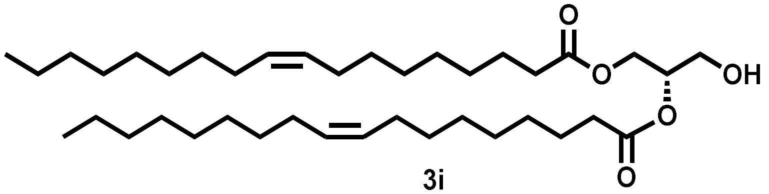

3i

The colorless oil **3i** was synthesized according to the general procedure. An isolated yield of 63% was obtained using hexane/ethyl acetate (20/1) as the eluents. The NMR data is consistent with the reported literature[7].

^{1}H NMR (400 MHz, $CDCl_3$) δ 5.54 – 5.21 (m, 4H), 5.15 – 4.95 (m, 1H), 4.32 (dd, *J* = 12.0, 4.5 Hz, 1H), 4.23 (dd, *J* = 11.9, 5.6 Hz, 1H), 3.88 – 3.28 (m, 2H), 2.33 (dt, *J* = 8.8, 7.5 Hz, 4H), 2.14 – 1.83 (m, 8H), 1.77 – 1.49 (m, 4H), 1.45 – 1.14 (m, 40H), 1.04 – 0.59 (m, 6H).

HRMS m/z (ESI): calcd. for $C_{39}H_{72}N_5$ $[M+Na]^+$: 643.5272; found: 643.5262.

*(R)-2-(oleoyloxy)-3-(stearoyloxy)propyl (2-(trimethylammonio)ethyl) phosphate (**3j**)*

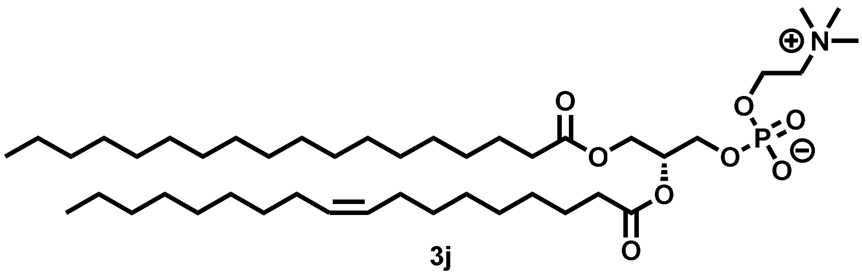

3j

The white solid **3j** (SOPC) was synthesized according to the general procedure. An isolated yield of 64% was obtained using $MeOH/CHCl_3/H_2O$ (50/50/2–80/20/2) as the eluents. The NMR data is consistent with the reported literature[4].

^{1}H NMR (400 MHz, $CDCl_3$) δ 5.50 – 5.26 (m, 2H), 5.19 (d, *J* = 7.1 Hz, 1H), 4.38 (dd, *J* = 12.0, 2.7 Hz, 1H), 4.30 (s, 2H), 4.11 (dd, *J* = 12.0, 7.4 Hz, 1H), 3.92 (q, *J* = 6.3 Hz, 2H), 3.77 (s, 2H), 3.33 (s, 9H), 2.28 (dt, *J* = 10.8, 7.6 Hz, 4H), 2.00 (q, *J* = 6.4 Hz, 4H), 1.71 – 1.47 (m, 4H), 1.34 – 1.16 (m, 48H), 0.87 (t, *J* = 6.7 Hz, 6H).

HRMS m/z (ESI): calcd. for $C_{44}H_{86}NO_8P$ $[M+H]^+$: 788.6164; found: 788.6167.

*(R)-2-(palmitoyloxy)-3-(stearoyloxy)propyl (2-(trimethylammonio)ethyl) phosphate (**3k**)*

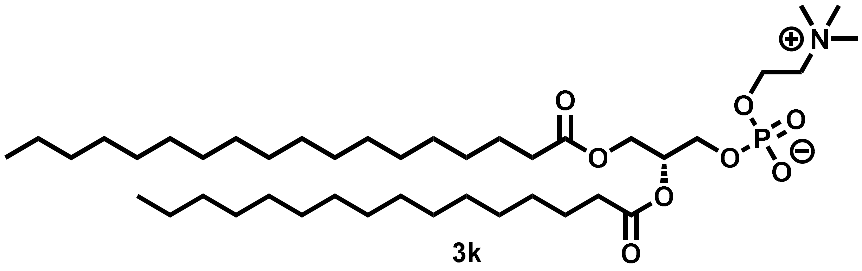

3k

The white solid **3k** (SPPC) was synthesized according to the general procedure. An isolated yield of 60% was obtained using MeOH/$CHCl_3$/H_2O (50/50/2–80/20/2) as the eluents. The NMR data is consistent with the reported literature[4].

^{1}H NMR (400 MHz, $CDCl_3$) δ 5.19 (d, J = 6.5 Hz, 1H), 4.69 – 4.26 (m, 3H), 4.11 (dd, J = 12.0, 7.5 Hz, 1H), 3.95 (d, J = 7.5 Hz, 2H), 3.81 (s, 2H), 3.35 (s, 9H), 2.28 (dt, J = 10.3, 7.5 Hz, 4H), 1.56 (s, 4H), 1.25 (s, 52H), 0.87 (t, J = 6.7 Hz, 6H).

HRMS m/z (ESI): calcd. for $C_{42}H_{84}NO_8P$ $[M+H]^+$: 762.6007; found: 762.6014.

5. NHPI ester 2a stability

A lipid film was prepared as described before (section 4) and hydrated with 100 µL of the indicated buffer to obtain a mixture containing **1a** (2 mM), **2a** (1 mM), and BNAH (3 mM). For HPLC-ELSD-MS analyses, 20 µL of the dispersion were mixed with 80 µL of MeOH and analyzed after 0 min and 10 min incubation. The amount of the NHPI ester **2a** was determined by integration of the HPLC peak observed at 205 nm. The relative amount of **2a** was calculated using the following equation: relative amount (%) $= \frac{A(205)\text{ of }\mathbf{2a}\text{ at 10 min}}{A(205)\text{of }\mathbf{2a}\text{ at 0 min}} \times 100$.

A

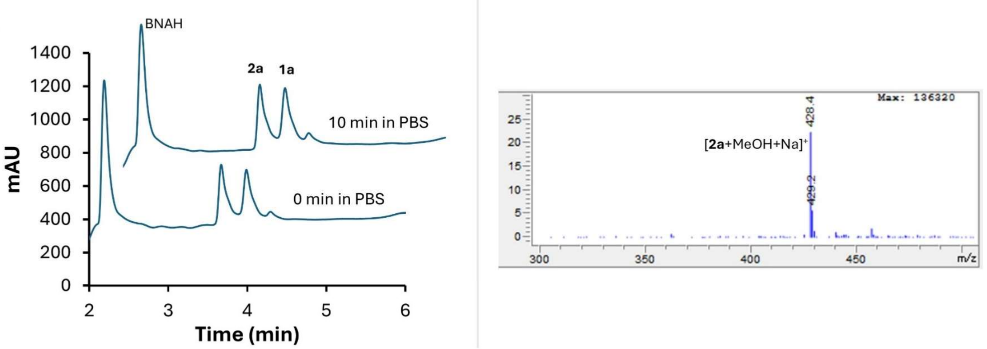

B

Buffer	PBS	PBS + 1 mM lysine	PBS + 0.5 mM sphingosine	DMEM	DMEM + 10% FBS
0 min (relative amount of **2a**)	100	100	100	100	100
10 min (relative amount of **2a**)	96.3	89.7	99.6	90.4	90.6

Supplementary Fig. 2. NHPI ester **2a** stability. (A) Representative HPLC (205 nm) traces of a lipid dispersion containing **1a** (2 mM), **2a** (1 mM), and BNAH (3 mM) at 0 min and 10 min in PBS (*left*), and LRMS corresponding to compound **2a** (*right*). (B) Comparison of the relative amount of NHPI **2a** in different aqueous buffers at 0 min and 10 min.

6. Reaction of a non-amphiphilic acrylate with NHPI ester 2a

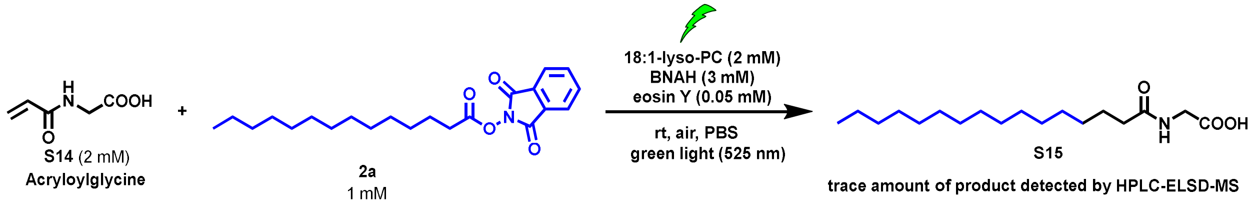

Supplementary Fig. 3. Reaction of *N*-acryloylglycine **S14** with NHPI ester **2a** using 18:1-lyso-PC as detergent. Only trace product was detected, possibly because the acryloylglycine precursor cannot form mixed micelles with the NHPI ester **2a**. The protocol follows the general procedure for the synthesis of phospholipid **3a** except for the addition of 18:1-lyso-PC as a detergent since the starting materials are not soluble in PBS.

7. Reaction kinetics of POPC synthesis under green light irradiation

The in situ synthesis of compound **3b** (POPC) was performed as described above (Note: No stirring was performed or shaking of the solution during the reaction). At different time points (0 s, 5 s, 10 s, 20 s, 30 s, 60 s, 120 s, 300 s), the light was turned off and 20 μL of reaction solution was aliquoted. Each aliquot was combined with 80 μL of MeOH, mixed by vortexing, and analyzed by HPLC-ELSD-MS. The ELSD signals of **1b**, **2b**, and POPC were utilized for monitoring reaction kinetics. The reaction was repeated three times for calculating standard deviations.

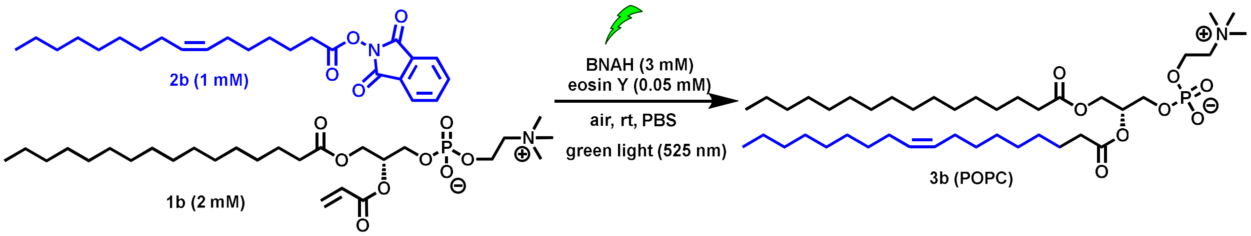

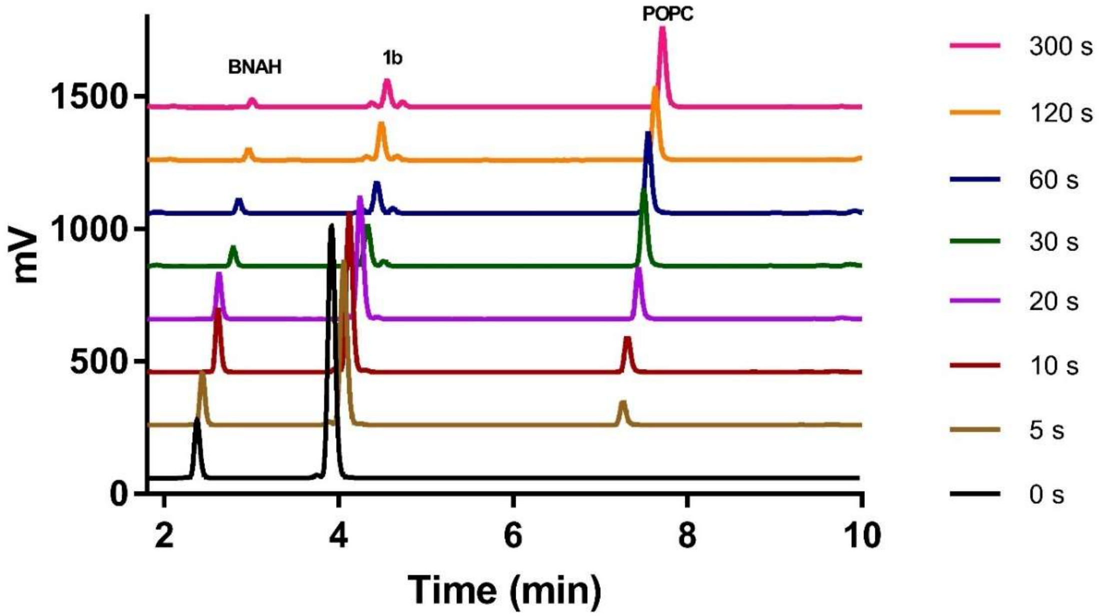

Supplementary Fig. 4. HPLC-ELSD traces of POPC vesicles formation obtained from the reaction between **1b (**2 mM), **2b (**1 mM), BNAH (3 mM) and eosin Y (0.05 mM) in PBS (pH 7.4) buffer under irradiation of green light at different time points.

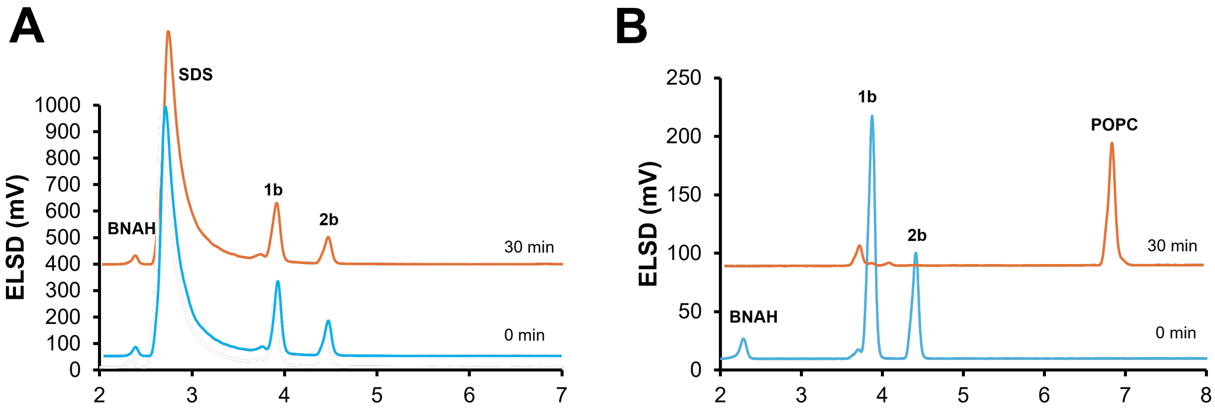

Supplementary Fig. 5. HPLC-ELSD traces of POPC synthesis in the presence (A) and absence (B) of 40 mM SDS. Blue traces correspond to the reaction at 0 min (*bottom*), brown traces represent the reaction after 30 min of green light (525 nm) irradiation (*top*).

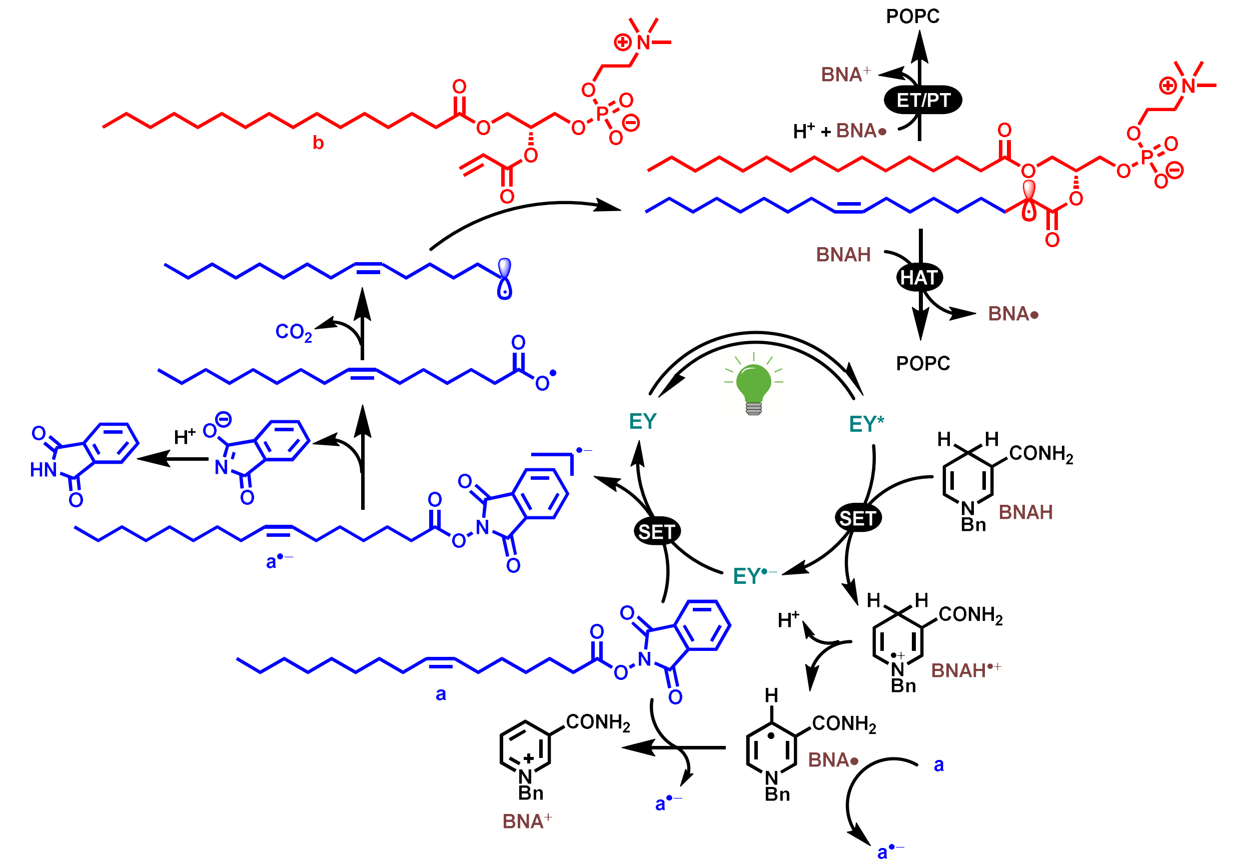

Supplementary Fig. 6. Proposed mechanism for photoredox lipid ligation based on prior literature[8,9].

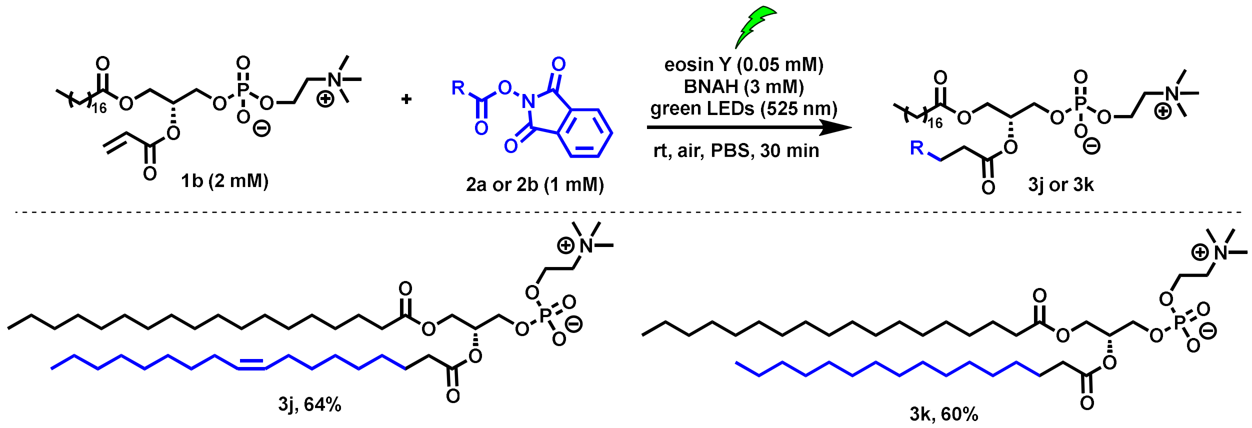

Supplementary Fig 7. Synthesis of phospholipids **3j** and **3k** by PLL. Isolated yields are shown.

8. In situ POPC (3b) vesicle growth under green light

The in situ synthesis of compound **3b** was performed as described above (section 4). The microscopy samples were prepared by depositing 1-2 µL of the reaction mixture on a microscope glass slide, topping with a coverslip (VWR micro cover glass, Cat. No. 48366-045, 0.13 to 0.17 mm thickness, 18x18mm). The reaction was imaged by phase contrast microscopy. The light for PLL was provided by the microscope excitation source passed through a bandpass filter (excitation wavelength 530 nm – 580 nm). Images were collected at 0 min, 15 min, 30 min, and 60 min at the same position on the slide. For video recordings, the images were collected for 30 min at about 5 frames per second. Negative controls were collected by keeping the mixture in darkness for 30 min using aluminum foil.

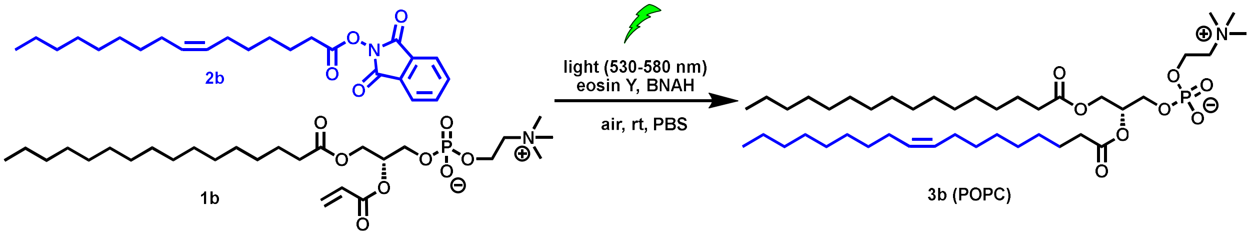

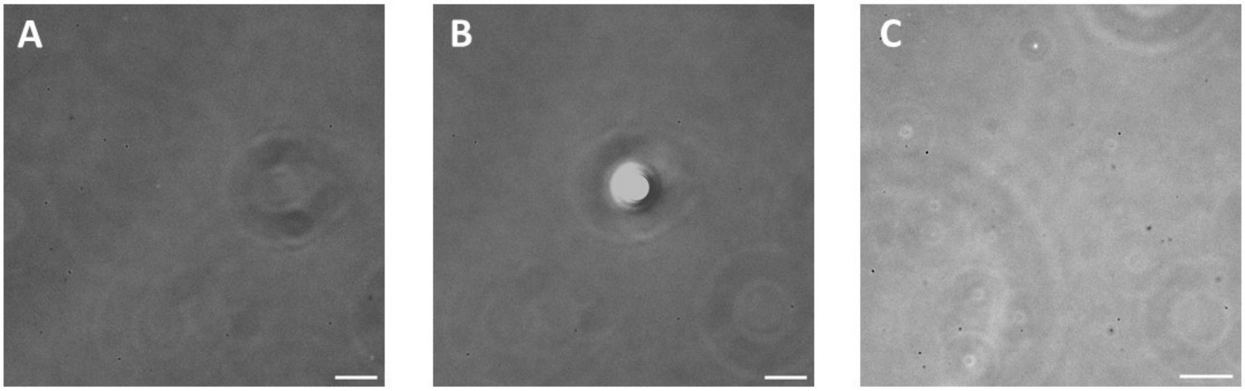

Supplementary Fig. 8. Phase contrast microscopy images of control experiments. (A) Phase contrast microscopy image of lysolipid **1b** (2 mM) in PBS buffer. (B) Phase contrast microscopy image of NHPI ester **2b** (1 mM) in PBS buffer. The bright circle at the center of the image is an oil droplet of **2b**. (C) Phase contrast microscopy image of a mixture of NHPI ester **2b** (1 mM), lysolipid **1b** (2 mM), BNAH (3 mM), and eosin Y (0.05 mM) in PBS buffer before the green light irradiation. Scale bar, 20 µm.

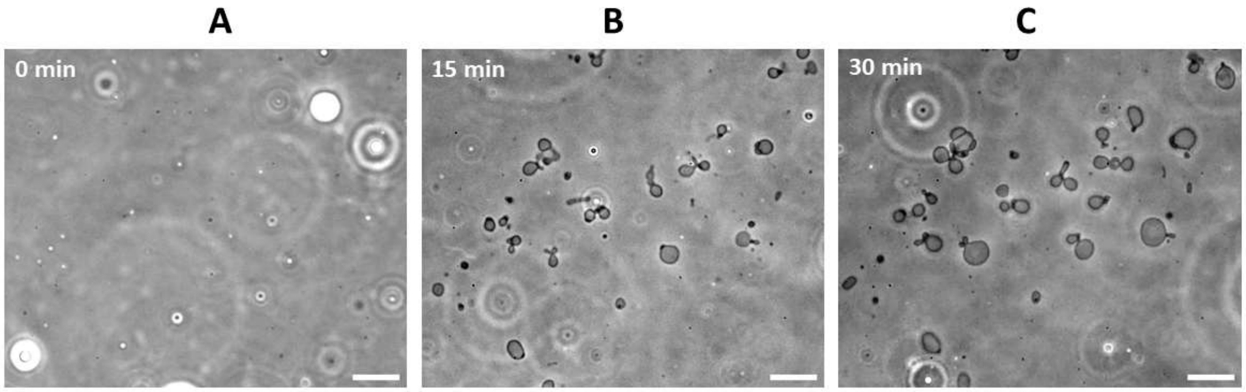

Supplementary Fig. 9. Phase contrast microscopy images of the in situ formation of POPC on a glass slide at (A) the beginning of reaction, (B) after 15 min, and (C) 30 min of reaction under green light irradiation. The images for (B) and (C) were recorded at the same position on the glass slide. Scale bar, 20 μm.

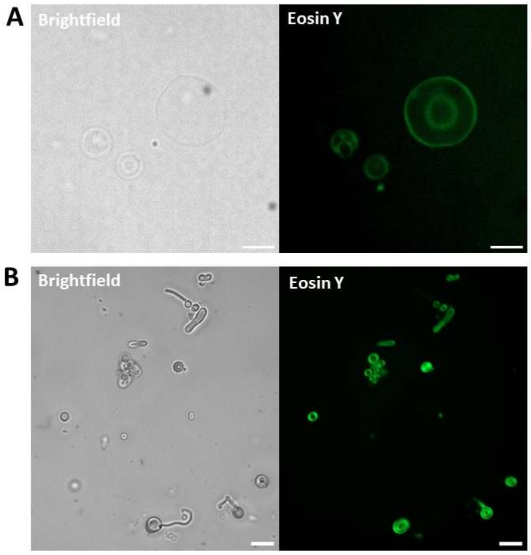

Supplementary Fig. 10. (A) Brightfield (*left*) and fluorescence (*right*) microscopy images of de novo POPC vesicle formation between **1b** (0.83 mM), **2b** (1.25 mM), BNAH (2.5 mM), and eosin Y (0.05 mM) on a glass slide after 30 min green light irradiation. Scale bar, 10 μm. (B) Brightfield (*left*) and fluorescence (*right*) microscopy images of POPC vesicles after hydration of a thin film of purified POPC with 0.05 mM eosin Y PBS buffer. Scale bar, 20 μm.

9. Encapsulation of fluorescent dyes or proteins during de novo vesicle formation

Encapsulation of eosin Y via in situ OPPC/POPC vesicle formation under green light

For the POPC lipid, to a 1-dram vial, 75 μL of **2b** (10 mM freshly prepared stock solution in $CHCl_3$), 50 μL of **1b** (10 mM in $CHCl_3$), 150 μL of BNAH (10 mM in $CHCl_3$), and 2.5 μL of eosin

Y (10 mM in MeOH) were added. For the OPPC lipid, to a 1-dram vial, 50 µL of **1a** (10 mM freshly prepared in $CHCl_3$), 75 µL of **2a** (10 mM in $CHCl_3$), 150 µL of BNAH (10 mM in $CHCl_3$), and 2.5 µL of eosin Y (10 mM in MeOH) were added. The solvent was evaporated under a gentle stream of N_2, and the content was redissolved in 300 µL CH_2Cl_2. The CH_2Cl_2 was evaporated again, while carefully rotating the vial to obtain a thin lipid film. The film was hydrated with 500 µL PBS buffer (pH = 7.4) and the suspension was sonicated for 1-2 min, until a red homogenous lipid dispersion was formed. The sample was irradiated with green LEDs at about 1 cm distance for 30 min, while being cooled by an electronic fan. (Note: no stirring or shaking was performed). After 30 min, 20 µL of the reaction solution was aliquoted and combined with 80 µL of MeOH, mixed by vortexing, and analyzed by HPLC-ELSD-MS. For microscopy analyses, 1-2 µL of the reaction mixture was taken and loaded onto a glass slide, covered with a glass coverslip. The samples were analyzed by phase contrast microscopy and Zeiss confocal microscopy with excitation wavelength 488 nm, emission wavelength 519 nm.

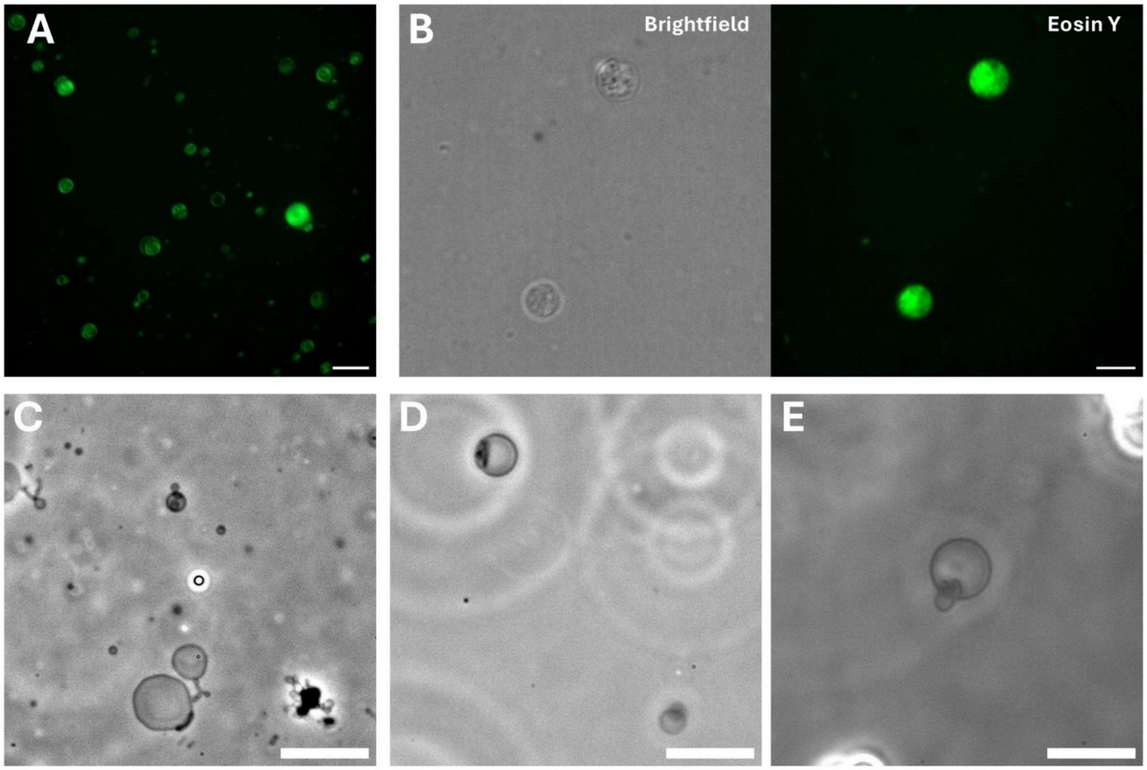

Supplementary Fig. 11. Spontaneous eosin Y encapsulation during the de novo formation of OPPC and POPC vesicles. (A) Fluorescence microscopy image of an in situ formed OPPC vesicle. Scale bar, 20 µm. (B) Bright field (*left*) and fluorescence (*right*) microscopy images of in situ formed POPC vesicles. Scale bar, 10 µm. (C-E) Comparison of POPC vesicle shapes by different formation methods including de novo formation on glass slide (C), de novo formation in glass vial (D), and direct hydration of POPC with PBS buffer (E). Scale bar, 20 µm.

Encapsulation of fluorescent protein mCherry by in situ POPC vesicle formation under green light

A lipid film containing **1b**, **2b**, BNAH, and eosin Y was prepared as described above (section 4), and hydrated with 500 μL mCherry in PBS buffer (pH=7.4). The suspension was sonicated for 10 s until a homogenous lipid dispersion was obtained. The sample was irradiated by green LEDs at about 1 cm distance for 30 min. After 30 min, 100 μL was transferred into a clean 0.5 mL Eppendorf tube, which contained His60 Ni Magnetic Beads (TaKaRa Bio USA, Inc., Cat. No. 635692) (washed by PBS 3 times before use). The resulting mixture was tumbled at room temperature for 30 min to remove the unencapsulated protein. The magnetic beads were removed by using a magnetic separation stand (Promega MagneSphere, Cat. No. Z5343) as per the manufacturer's instructions. Afterwards, 1-2 μL solution was taken and loaded onto a glass slide, which was sealed by a glass coverslip. The samples were then imaged by confocal microscopy with excitation wavelength 561 nm, emission wavelength 610 nm.

10. Cryogenic electron microscopy (cryoEM) images of vesicles formed by PLL

Photogenerated OPPC vesicles in PBS buffer were prepared as previously described. Samples were deposited onto 300 mesh Lacey carbon grids on copper (Electron Microscopy Services). The grids were glow discharged at 20 mA for 45 s in a K100X instrument (Emitech). 4 μL of vesicle sample solution was applied to the grid and vitrified using a Vitrobot Mark IV (ThermoFisher) by plunge-freezing into liquid ethane cooled by liquid nitrogen, using 4 s blot times and a 4 blot-force setting, at 4 ºC and 100% humidity. Samples were stored in liquid nitrogen until use. CryoEM data were collected on a Talos Arctica (FEI) operated at 200 kV, equipped with a K2 Summit direct electron detector (Gatan). Data collection was performed with Leginon[9]. Images were collected with a total dose of 54 e/Å2 at 1.4 Å/pixel and a 3 μm nominal defocus. Micrograph frames were aligned with the Cryosparc v3.3.1 patch alignment tool[10]. Images were processed using ImageJ or Fiji.

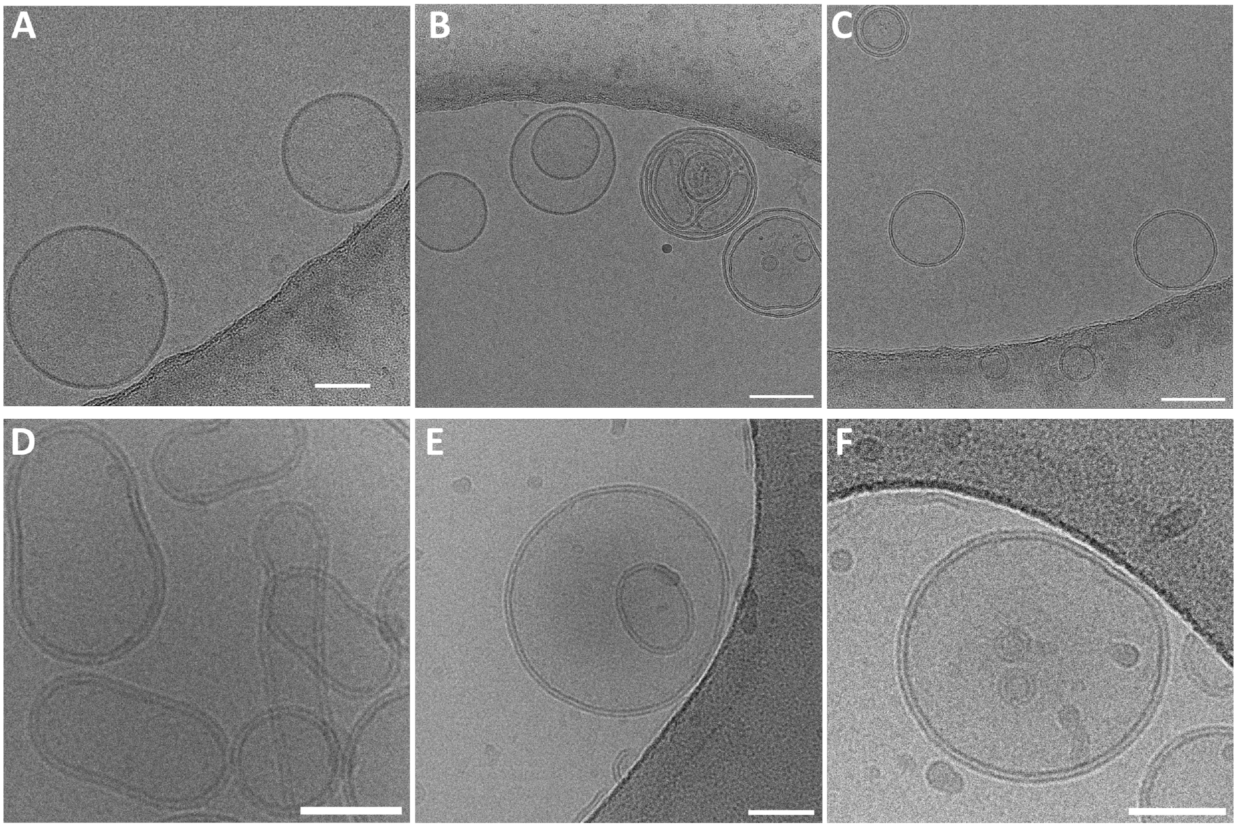

Supplementary Fig. 12. (A-C) Representative cryoEM images of OPPC vesicles formed after reaction between **1a** (1 mM), **2a** (1.5 mM), BNAH (3 mM) in 500 μL PBS under 40 W Kessil A160WE blue LEDs irradiation for 30 min. The results confirm the formation of a bilayer structure. (A) scale bar, 50 nm; (B) and (C) scale bar, 100 nm. (D-F) Representative cryoEM images of POPC vesicles formed after reaction between **1b** (1 mM), **2b** (1.5 mM), BNAH (3 mM), and eosin Y (0.05 mM) in 500 μL PBS (pH 7.4) after 30 min green LEDs irradiation. Scale bar, 25 nm.

11. De novo formed OPPC vesicles that have spontaneously entrapped eosin Y can catalyze new phospholipid synthesis

De novo formed OPPC vesicles containing eosin Y in PBS buffer were prepared as previously described (section 4). 500 μL of the vesicle dispersion were purified by spin filtration (2000 rpm for 2 min) using a PD SpinTrap G-25 (Cytiva, 28918004) to remove the unbound eosin Y.

To a 1-dram vial, 100 μL **1a** (10 mM freshly prepared in $CHCl_3$), 50 μL **2b** (10 mM in $CHCl_3$), 150 μL BNAH (10 mM in $CHCl_3$) were added. The solvent was evaporated under a gentle stream of N_2, and the content was redissolved with 300 μL CH_2Cl_2. The CH_2Cl_2 was evaporated again, while carefully rotating the vial to obtain a thin lipid film. The film was hydrated with 500 μL of purified OPPC vesicles in PBS, and then the suspension was vortexed for 10 s until a homogenous lipid dispersion was obtained. The sample was irradiated with green LEDs at about 1 cm (Note: do not stir or shake during the irradiation), while being cooled by an electronic fan. At various time

points (1 min, 3 min, 5 min, and 10 min), 20 μL of the sample was taken and mixed with 80 μL MeOH and analyzed by HPLC-ELSD-MS using MeOH/H_2O/HCOOH as the eluents. The concentration of DOPC formed was determined based on the area of the ELSD signal by comparison to a standard curve of DOPC. The yield was calculated using the following equation:

$$\text{yield (\%)} = \frac{\text{concentration of DOPC (mM)}}{\text{initial concentration of limiting reagent } \mathbf{2b} \text{ (mM)}} \times 100$$

HPLC analysis was carried out on an Eclipse Plus C8 analytical column with Phase A/Phase B gradients [Phase A: MeOH with 0.1% formic acid, Phase B: H_2O with 0.1% formic acid]. 50%-93% Phase A in Phase B, 1 min, 93%-95% Phase A in Phase B, 3 min, 95%-99% Phase A in Phase B, 8 min, 99%-50% Phase A in Phase B, 0.5 min, then 50% Phase A in Phase B, 0.5 min.

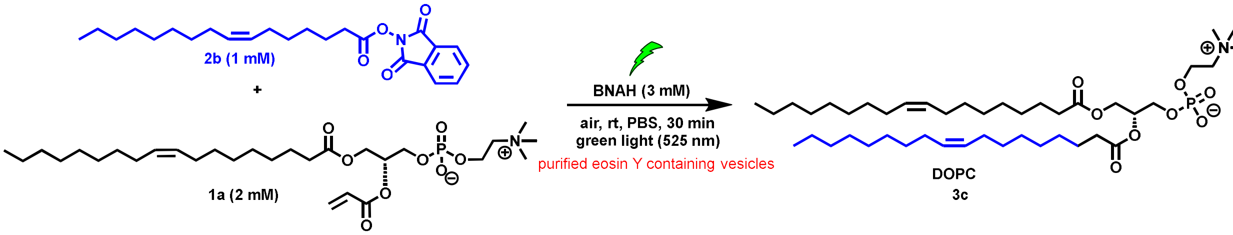

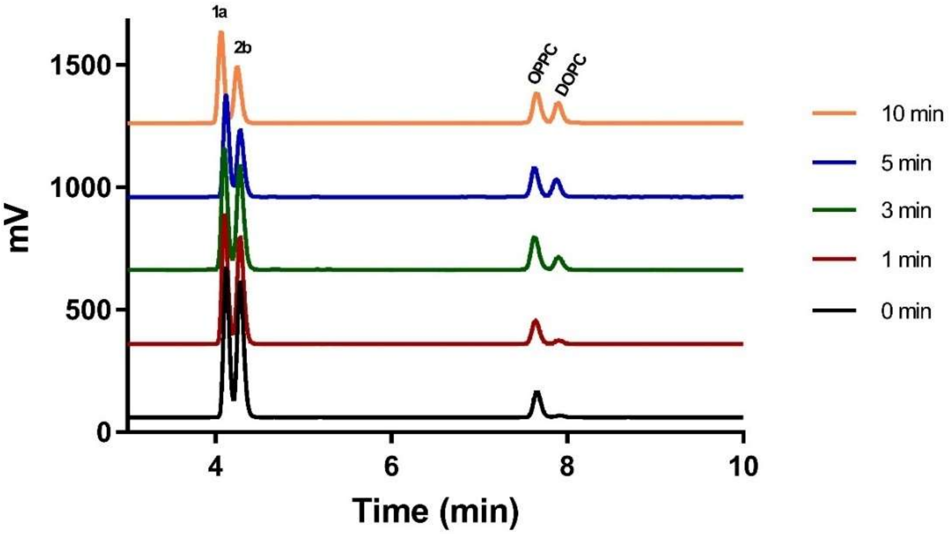

Supplementary Fig. 13. HPLC-ELSD traces of the in situ reaction between **1b** (2 mM), **2b** (1 mM), BNAH (3 mM), and eosin Y-containing OPPC vesicles to generate DOPC in PBS buffer (pH 7.4) under irradiation of green light at different irradiation time points.

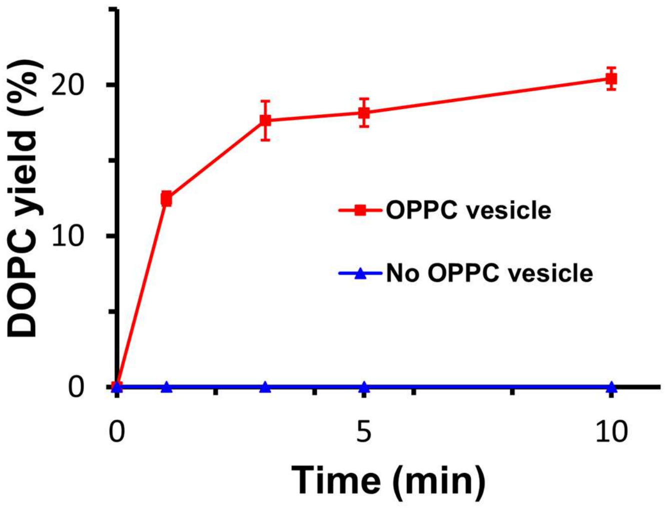

Supplementary Fig. 14. Kinetics of DOPC synthesis via PLL in the absence (*blue*) and presence (*red*) of purified eosin Y-containing OPPC vesicles. The data points are presented as means ± SD (n = 3 biologically independent samples).

12. De novo formed OPPC vesicles that have spontaneously entrapped eosin Y can catalyze new non-canonical phospholipid synthesis

The purified eosin Y-containing OPPC vesicles were prepared as previously described in section 11.

To a 1-dram vial, 100 µL of **1a** (10 mM freshly prepared in $CHCl_3$), 50 µL of **2e** (10 mM in $CHCl_3$), and 150 µL of BNAH (10 mM in $CHCl_3$) were added. The solvent was evaporated under a gentle stream of N_2, and the content was redissolved in 300 µL of CH_2Cl_2. The CH_2Cl_2 was evaporated again, while carefully rotating the vial to obtain a thin lipid film. The film was hydrated with 500 µL of purified eosin Y containing OPPC vesicles, and the suspension was vortexed for 10 s until a homogenous lipid dispersion was obtained. The sample was irradiated under green LEDs at about 1 cm for 30 min (Note: the sample was not stirred or shaken during the irradiation), while being cooled by an electronic fan. After 30 min, 20 µL of the sample was taken and mixed with 80 µL MeOH and analyzed by HPLC-ELSD-MS using MeOH/H_2O/HCOOH as the eluents.

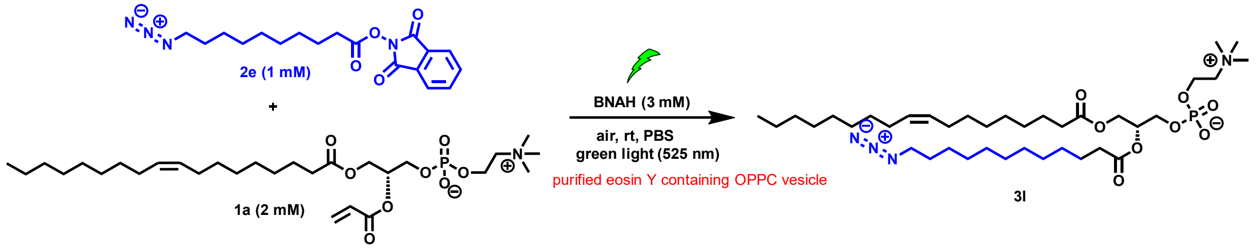

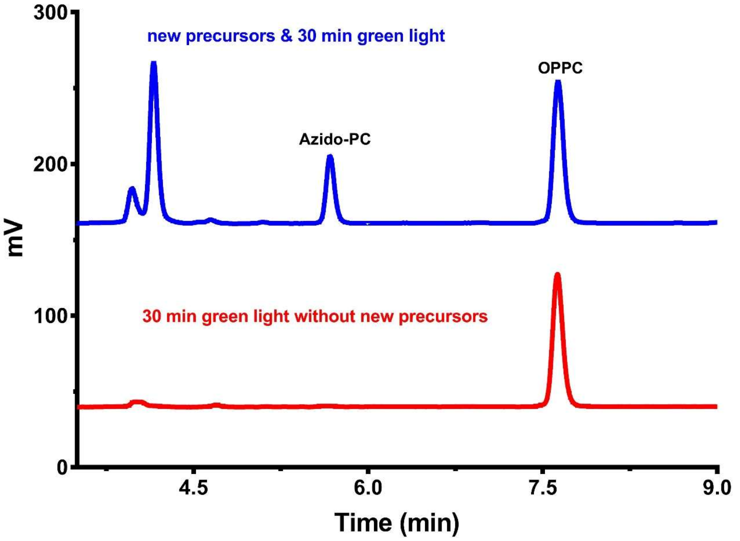

Supplementary Fig. 15. HPLC-ELSD traces of eosin Y containing OPPC vesicles catalyzing the synthesis of non-canonical phospholipid **3l**. Red line (*bottom*): control experiment of purified eosin Y-containing OPPC vesicles after irradiation with green light for 30 min; Blue line (*top*): purified eosin Y containing OPPC vesicles were mixed with **1a** (2 mM), **2e** (1 mM), BNAH (3 mM), followed by green light irradiation for 30 min.

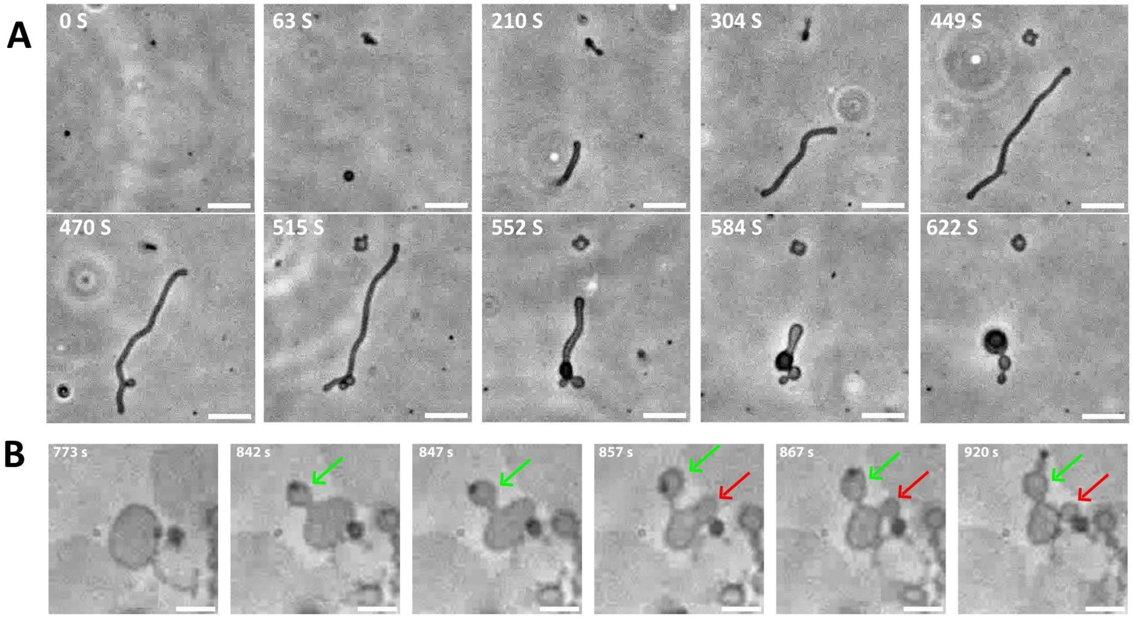

Supplementary Fig. 16. (A) Phase contrast time-lapse microscopy images of de novo POPC vesicle formation and growth on a glass slide under the irradiation of green light. Scale bar, 10 µm. (B) Phase contrast time-lapse microscopy images of POPC vesicle division on a glass slide under the irradiation of green light. The vesicle inside the white dashed circle is monitored over time. Scale bar, 5 µm.

13. De novo formation of POPC:DPPC mixed vesicles under green light

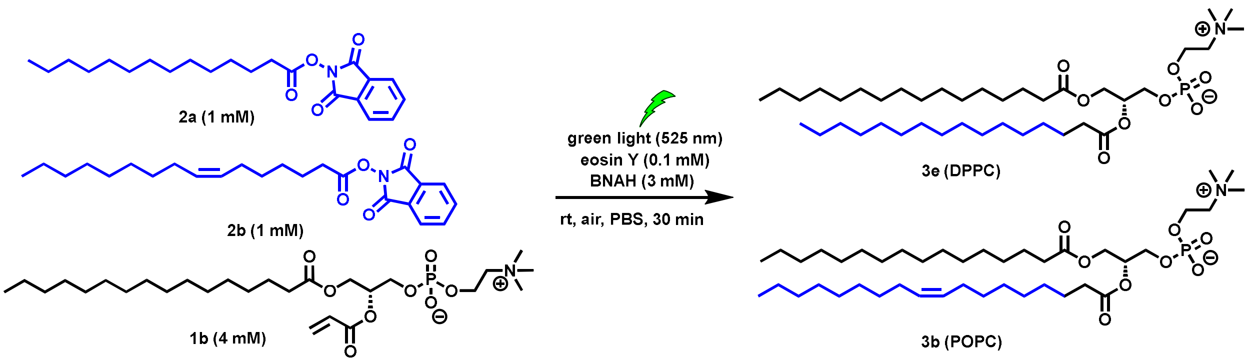

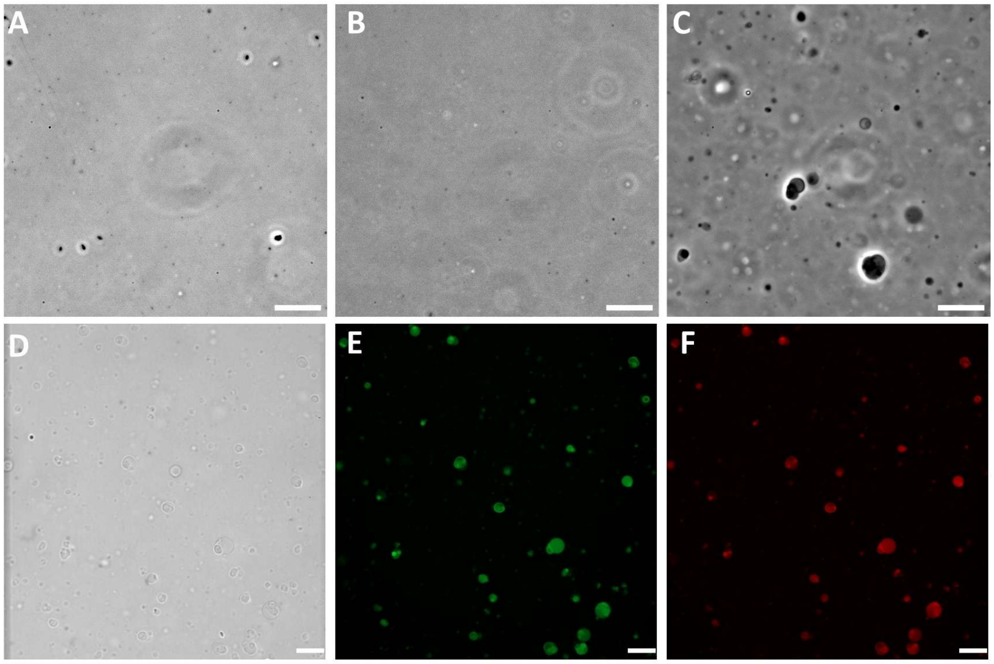

Supplementary Fig. 17. (A-C) Phase contrast microscopy images of de novo DPPC:POPC vesicle formation at (A) the beginning of the reaction, (B) after 30 min in darkness (control experiment), and (C) after 30 min of green light irradiation. Scale bar, 20 μm. (D-F) Brightfield (D) and fluorescence microscopy images (E and F) of de novo DPPC:POPC vesicles formed between compound **2a** (1 mM), compound **2b** (1 mM), compound **1b** (4 mM), and BNAH (3 mM) in the presence of eosin Y (0.1 mM) after green light irradiation for 30 min. Nile red (1 mol%) was added before imaging. (B) eosin Y channel, (C) Nile red channel. Scale bar, 20 μm.

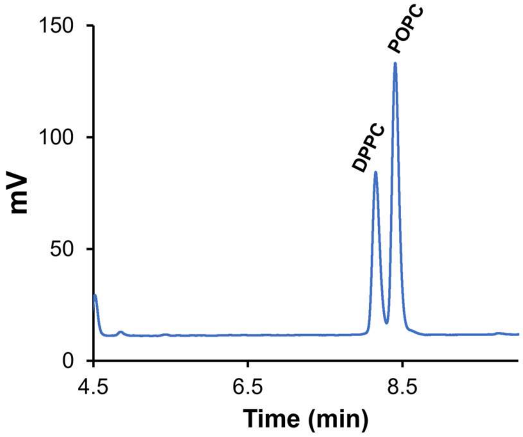

Supplementary Fig. 18. HPLC-ELSD traces of the in situ reaction between compound **2a** (1 mM), compound **2b** (1 mM), compound **1b** (4 mM), and BNAH (3 mM) in the presence of eosin Y (0.1 mM) after green light irradiation for 30 min in PBS buffer.

14. *In situ* POPC vesicle formation using a fluorogenic nucleic acid binding dye

To a 1-dram clear vial, 20 µL of **1b** (10 mM in $CHCl_3$), 10 µL of **2b** (10 mM in $CHCl_3$), and 30 µL of BNAH (10 mM in $CHCl_3$) were added. The solvent was evaporated under a gentle stream of N_2, and the content was redissolved in 100 µL of CH_2Cl_2. The CH_2Cl_2 was evaporated again, while carefully rotating the vial to obtain a thin lipid film. The film was hydrated with a 100 µL PBS buffer (pH = 7.4) containing either 15 µM TOTO-1 (ThermoFisher Scientific, Cat. No. T3600, Lot No. 2615826) and 30 ng/µL DNA or 15 µM TO1-3PEG-Desthiobiotin (TO1-Dtb, Applied Biological Materials Inc. (abm), Richmond, BC, Canada) and 30 ng/µL RNA aptamer. The suspension was sonicated for 1 min, until a homogenous lipid dispersion was formed. The vial was irradiated with 14 W green LEDs (λ_{max} = 525 nm) at approximately 1 cm distance for 30 min, while being cooled by an electronic fan. After 30 min, 20 µL of the solution was mixed with 80 µL MeOH and analyzed by HPLC-ELSD-MS. For the phase contrast microscopy analyses, 1-2 µL of the reaction mixture was loaded onto a glass slide and imaged by phase contrast microscopy. For the time-lapse phase contrast microscopy analyses, the procedure is the same as section 8. Images were collected at 0 min, 10 min, 20 min, and 30 min at the same position on the slide.

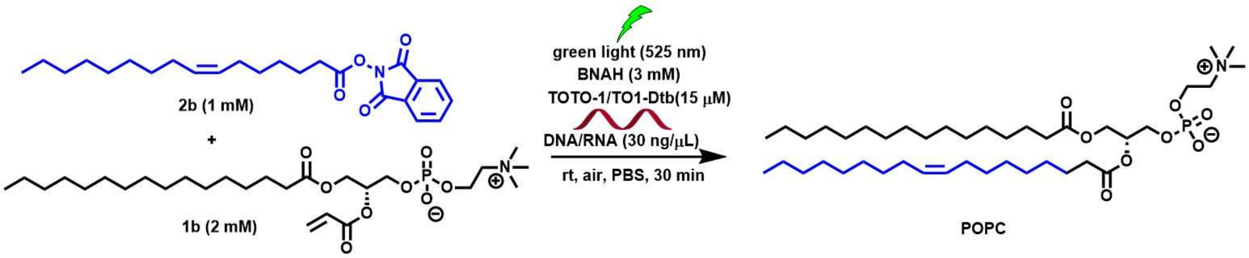

Supplementary Table 5. POPC synthesis in the presence of DNA binding dye.

Entry	Conditions	Yield POPC (%)[a]
1	**2b** (1 mM), **1b** (2 mM), BNAH (3 mM), DNA (30 ng/μL)/TOTO-1 (15 μM)	54 ± 13
2	**2b** (1 mM), **1b** (2 mM), BNAH (3 mM), DNA (30 ng/μL)	0
3	**2b** (1 mM), **1b** (2 mM), BNAH (3 mM), TOTO-1 (15 μM)	0
4	**2b** (1 mM), **1b** (2 mM), BNAH (3 mM), DNA (30 ng/μL)/TOTO-1 (15 μM)	0

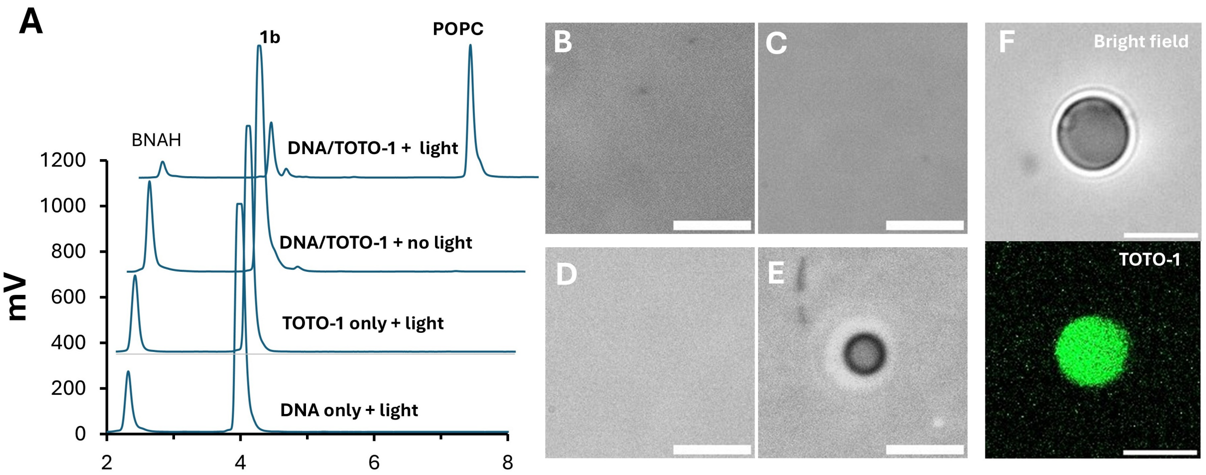

Supplementary Fig. 19. POPC vesicle formation between **2b** (1 mM), **1b** (2 mM), BNAH (3 mM) in the presence of nucleic acid (30 ng/μL) and TOTO-1 (15 μM) in PBS buffer under 30 min green light irradiation. (A) HPLC-ELSD traces of POPC formation in the presence of DNA/TOTO-1 and various controls. (B) Phase contrast image of the control reaction in the presence of DNA (30 ng/μL) and absence of TOTO-1, under 30 min green light. (C) Phase contrast image of the control reaction in the presence of TOTO-1 (15 μM) and absence of DNA, under 30 min green light irradiation. (D) Phase contrast image of the control reaction in the presence of both DNA (30 ng/μL) and TOTO-1 (15 μM) in dark conditions for 30 min. (E) Representative phase contrast image of vesicles observed from the reaction in the presence of DNA (30 ng/μL) and TOTO-1 (15 μM) under 30 min green light irradiation. Scale bar, 10 μm. (F) Representative microscopy images of a vesicle formed during the synthesis of POPC in the presence of 15 μM TOTO-1 and 10 ng/mL DNA. Bright field image (*up*) and fluorescence microcopy image (*down*) show that DNA is encapsulated inside the de novo formed vesicle. Scale bar, 10 μm.

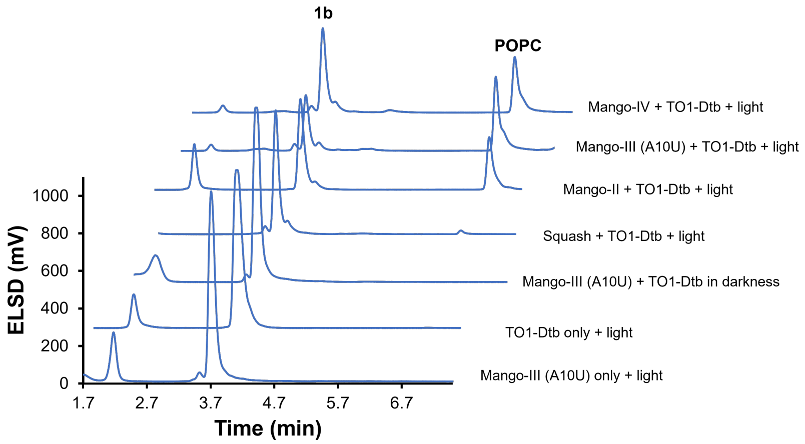

Supplementary Fig. 20. HPLC-ELSD traces of POPC vesicle formation between **2b** (1 mM), **1b** (2 mM), and BNAH (3 mM) in the presence of RNA aptamer (30 ng/µL) and TO1-Dtb (15 µM) in PBS buffer under green light irradiation for 30 min. Various controls are also shown as indicated.

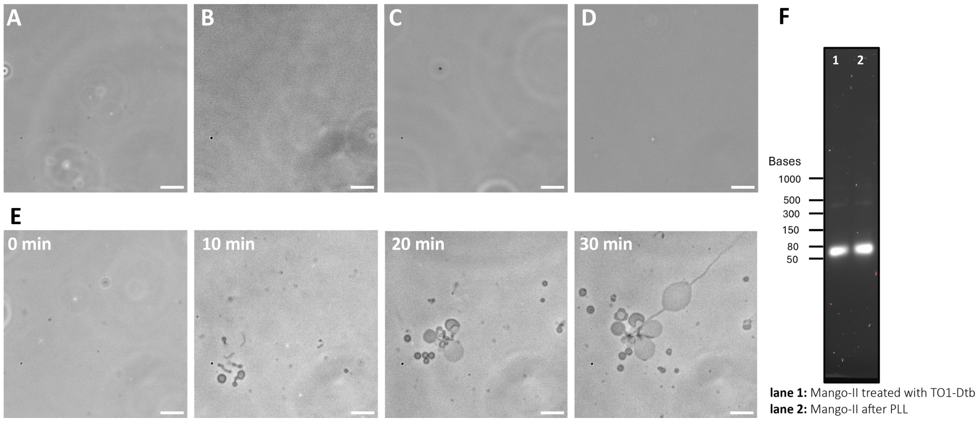

Supplementary Fig. 21. Phase contrast microscopy images of POPC vesicle formation during the reaction between **2b** (1 mM), **1b** (2 mM), and BNAH (3 mM) in the presence of Mango-III (A10U) (30 ng/µL)/TO1-Dtb (15 µM) under green light irradiation for 30 min. (A) Phase contrast images of a control reaction in the absence of both Mango-III (A10U) (30 ng/µL) and TO1-Dtb (15 µM) under green light irradiation for 30 min. (B) Phase contrast images of a control reaction in the presence of TO1-Dtb (15 µM) and Mango-III (A10U) under darkness for 30 min. (C) Phase contrast images of a control reaction in the presence of Mango-III (A10U) (30 ng/µL) only under green light irradiation for 30 min. (D) Phase contrast images of a control reaction in the presence of TO1-Dtb (15 µM) only under green light irradiation for 30 min. (E) Time-lapse phase contrast microscopy images of de novo POPC vesicle formation and growth on a glass slide in the presence of Mango-III (A10U) (30 ng/µL) and TO1-Dtb (15 µM) under green light irradiation for 30 min. Scale bar, 10 µm. (F) Agarose gel of Mango-II before and after PLL. No cleavage products could be detected, supporting Mango-II stability during PLL. After PLL, an amount of the reaction mixture corresponding to 100 ng of aptamer was heat denatured and run on a 2% agarose gel. Fresh aptamer (100 ng) was treated with TO1-3PEG-Desthiobiotin (TO1-Dtb) (15 µM), heat denatured and run as a comparison.

15. Nucleic acid design and sequences

DNA:

The following DNA sequence (1270 BP) was amplified using PCR (Q5® High-Fidelity, NEB) to yield linear DNA.

ttctctatgtgtgagacagggtctcgcctagaggtgatgcggttttggcagtacatcaatgggcgtggatagcggtttgactcacggggatttc
caagtctccaccccattgacgtcaatgggagtttgttttggcaccaaaatcaacgggactttccaaaatgtcgtaacaactccgccccattgac
gcaaatgggcggtaggcgtgtacggtgggaggtctatataagcagagctaaactaccccaagctggcctctgaggccaccatgggccgta
aaggagaagaacttttcactggtgtcgtccctattctggtggaactggatggtgatgtcaacggtcataagttttccgtgcgtggcgagggtga
aggtgacgcaactaatggtaaactgacgctgaagttcatctgtactactggtaaactgccggtaccttggccgactctggtaacgacgctgac
ttatggtgttcagtgctttgctcgttatccggaccatatgaagcagcatgacttcttcaagtccgccatgccggaaggctatgtgcaggaacgc
acgatttcctttaaggatgacggcacgtacaaaacgcgtgcggaagtgaaatttgaaggcgataccctggtaaaccgcattgagctgaaag
gcattgactttaaagaagatggcaatatcctgggccataagctggaatacaattttaacagccacaatgtttacatcaccgccgataaacaaaa
aaatggcattaaagcgaattttaaaattcgccacaacgtggaggatggcagcgtgcagctggctgatcactaccagcaaaacactccaatc
ggtgatggtcctgttctgctgccagacaatcactatctgagcacgcaaagcgttctgtctaaagatccgaacgagaaacgcgatcatatggtt
ctgctggagttcgtaaccgcagcgggcatcacgcatggtatggatgaactgtacaaaagcctcgactgtgccttctagttgccagccatctgt
tgtttgcccctcccccgtgccttccttgaccctggaaggtgccactcccactgtcctttcctaataaaatgaggaaattgcatcgcattgtctga
gtaggtgtcattctattctggggggtggggtggggcaggacagcaagggggaggattgggaagacaatagcaggcatgctggggatgc
ggtgggctctatggcttctgaggcggaaagaaccagctgggcctactagatagcggaccccttacc

Primers:

forward: TTCTCTATGTGTGAGACAGGG

reverse: GGTAAGGGGTCCGCTATCTAG

PCR amplified linear DNA was purified using a PureLink™ PCR Purification Kit (Thermo Fisher Scientific) according to the manufacturer’s instructions.

RNA Aptamers:

RNA aptamers were designed based on the appropriate sequence with an elongated stem loop[12]. The aptamers were prepared by Cisterna Biologics, using T7 transcription from a DNA template. Sequences are given below.

Mango-II:

GCGAGCACGUACGAAGGAGAGGAGAGGAAGAGGAGAGUACGUGCUCG

Mango-III (A10U):

GGCACGUACGAAGGAAGGUUUGGUAUGUGGUAUAUUCGUACGUGCC

Mango-IV:

GGCACGUACCGAGGGAGUGGUGAGGAUGAGGCGAGUACGUGC

Squash:

GGGAAGAUACAAGGUGAGCCCAAUAAUAUGGUUUGGGUUAGGAUAGGAAGUAGA
GCCUUAAACUCUCUAAGCGGUAUCUUCCC

16. HeLa cell membrane labeling by PLL

HeLa cells were purchased from American Type Culture Collections (Manassas, VA). HeLa cells were cultured in DMEM (Gibco^{TM}, no Phenol red) supplemented with 10% FBS and 1% Penicillin-Streptomycin. Cell cultures were maintained at 37 °C and 5% CO_2 in a humidified atmosphere. All cultures were intermittently evaluated for the presence of mycoplasma contamination via a mycoplasma detection kit (MycoStrip, Invivogen).

Stock solutions for cell treatment were prepared as follows. For stock solution A, 60 µL of lyso-lipid **1b** (10 mM in $CHCl_3$), 60 µL of **2e** (10 mM in $CHCl_3$), 120 µL of BNAH (10 mM in $CHCl_3$), and 7.5 µL of eosin Y (10 mM in MeOH) were mixed in a 2-dram amber vial. For stock solution B, 60 µL of **2e** (10 mM in $CHCl_3$), 120 µL of BNAH (10 mM in $CHCl_3$), and 7.5 µL of eosin Y (10 mM in MeOH) were mixed in a 2-dram amber vial. The solvent was evaporated under a gentle stream of N_2, and the content was redissolved in 300 µL CH_2Cl_2. The CH_2Cl_2 was evaporated again, while carefully rotating the vial to obtain a thin lipid film. The film was hydrated with 4 mL phenol red free DMEM, and the suspension was sonicated for 1-2 min until a homogeneous lipid dispersion was formed.

HeLa cells, maintained in DMEM (10% FBS, 1% penicillin/streptomycin), were plated in a 33 mm dish at a density of 50,000 cells/dish. After 24 h of incubation at 37 °C, 5% CO_2, the cell medium was discarded. For the untreated group, HeLa cells were incubated in phenol red free DMEM for 5 min. For the treated group, cells were treated with 1 mL freshly prepared stock solution A, and then irradiated by an UltraFire Green Hunting Flashlight for 5 min. For one control group, cells were incubated with 1 mL freshly prepared stock solution A in a dark incubator for 5 min. For another control group, cells were incubated with 1 mL freshly prepared stock solution B under green light irradiation for 5 min. For the last control group, cells were incubated with 1 mL freshly prepared stock solution A (but in absence of eosin Y) under green light irradiation for 5 min. Subsequently, the medium of the four dishes (experiment and three controls) was discarded, and the cells were washed with phenol red free DMEM three times. The HeLa cells were incubated with 0.5 mL of a 20 µg/mL DBCO-Fluor-594 (BroadPharm, Cat. No. BP-25578) in DMEM for 5 min. Afterwards, the cells were washed with DMEM three times and then imaged by confocal microscopy with excitation wavelength 561 nm, emission wavelength 618 nm.

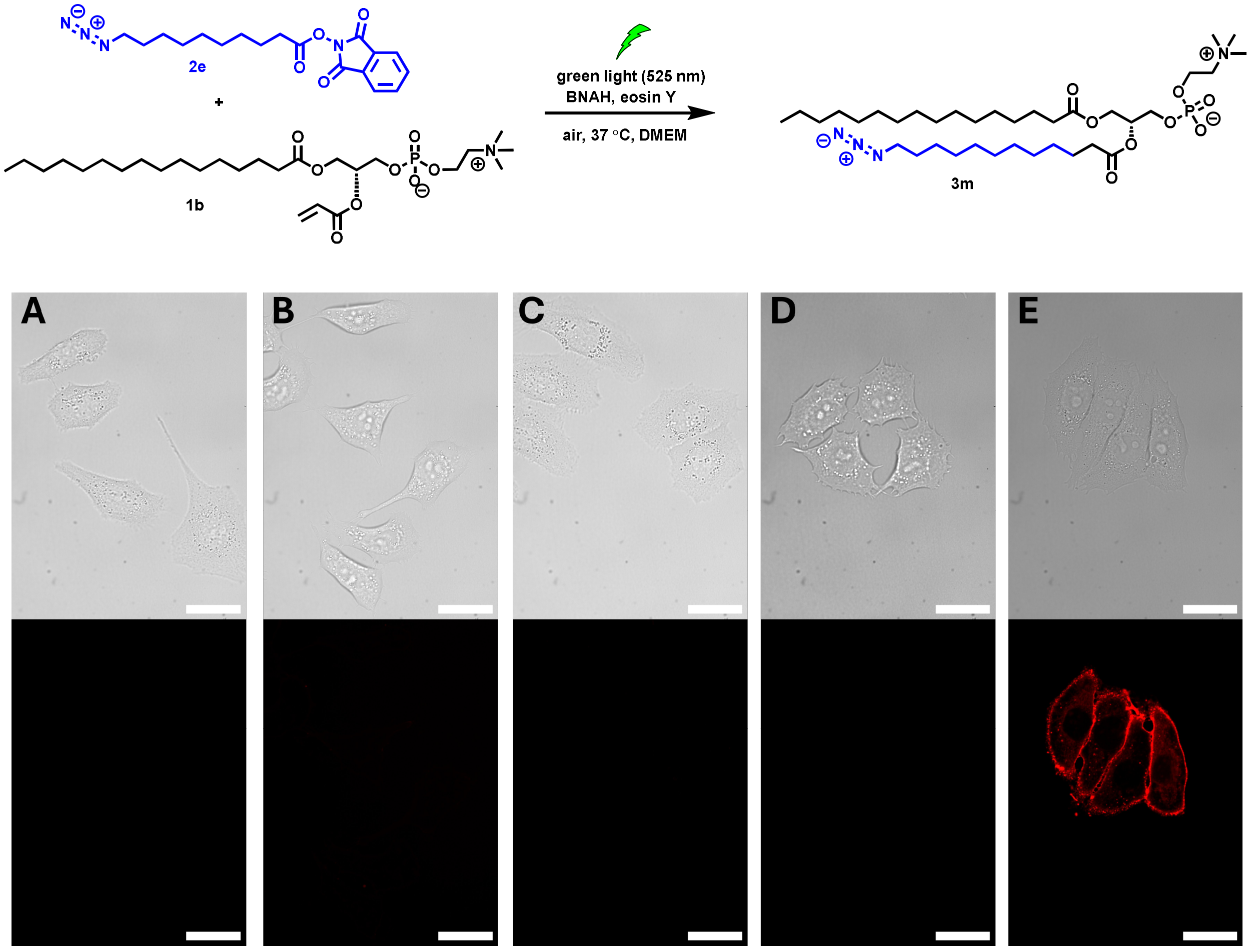

Supplementary Fig. 22. Confocal microscopy images of HeLa cells labeled by PLL. (A) HeLa cells (untreated) were incubated in phenol red free DMEM for 5 min. (B) HeLa cells were treated by stock solution A in the dark and incubated for 5 min. (C) HeLa cells were incubated with stock solution B and irradiated with green light for 5 min. (D) HeLa cells were incubated with a solution identical to solution A but without eosin Y, and irradiated with green light for 5 min. (E) HeLa cells were incubated with stock solution A and irradiated with green light for 5 min. Scale bar 50 μm.

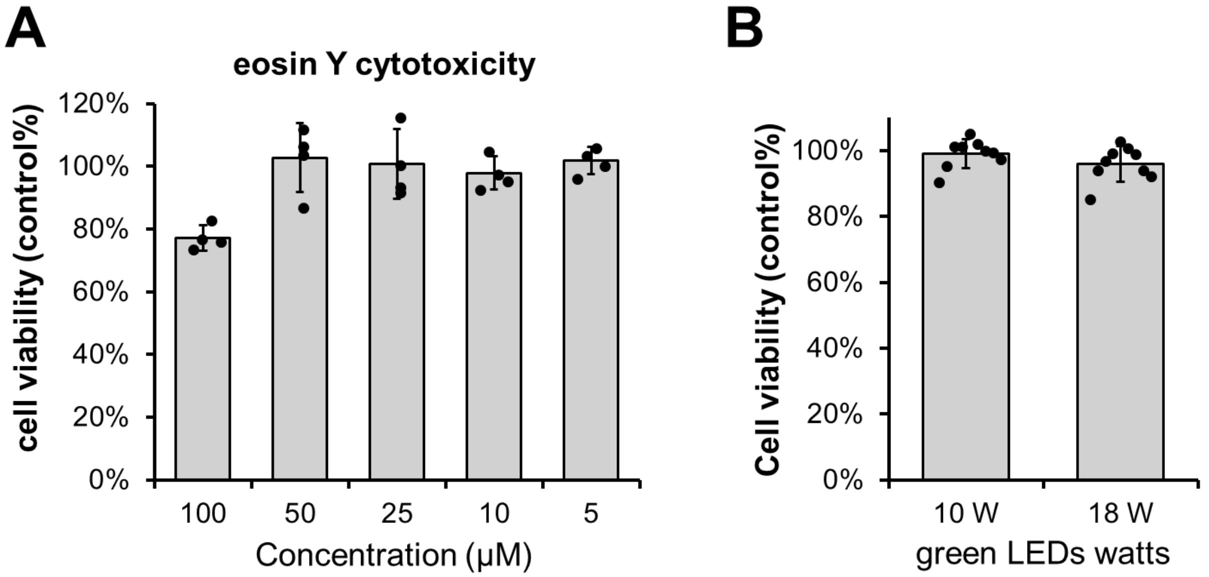

Supplementary Fig. 23. Cell proliferation assay (CCK-8) of HeLa cells upon the treatment of eosin Y with indicated concentration followed by incubation for 60 hours (A) or green LEDs (525 nm) with different intensity for five minutes (B). In the PLL cell-based experiments, the concentration of eosin Y employed was less than 10 μM, and the green LED power used was 10 W. The data points are presented as means ± SD (n = 4 technically independent samples for figure A. n = 9 technically independent samples for figure B).

17. HRMS quantification of ceramide synthesis in live cells

Stock solution preparation: 80 μL of compound **1f** (10 mM in $CHCl_3$), 160 μL of deuterated NHPI ester **2d** (10 mM in $CHCl_3$), 240 μL of BNAH (10 mM in $CHCl_3$), 80 μL of C18:1-lyso-PC (10 mM in $CHCl_3$), and 4 μL of eosin Y (10 mM in MeOH) were mixed in a 2-dram amber vial. The solvent was evaporated by a gentle stream of N_2, and the content was redissolved in 500 μL of CH_2Cl_2. The CH_2Cl_2 was evaporated again, while carefully rotating the vial to obtain a thin lipid film. The film was hydrated with 8 mL phenol red free DMEM, and the suspension was sonicated for 1 min until a homogeneous lipid dispersion was formed. The final concentration of the reagents was 100 μM for compound **1f**, 200 μM for deuterated NHPI ester **2d**, 300 μM for BNAH, and 5 μM for eosin Y. This freshly prepared stock solution was used directly for following cell treatment.

HeLa cells, maintained in DMEM (10 % FBS, 1% penicillin/streptomycin) were plated in triplicate using 6 cm culture dishes and grown to confluency at 37 ºC, 5% CO_2. Once confluent, the medium was discarded and then the appropriate volume of DMEM (10% FBS, 1% penicillin/streptomycin) was added. For the untreated group, HeLa cells were incubated with 3 mL phenol red free DMEM. For the treated group, the HeLa cells were pretreated with 2 mL of eosin Y (20 μM) in phenol red free DMEM for 30 min to penetrate cells. Afterwards, the HeLa cells were treated with 3 mL of

DMEM stock solution and incubated in the dark for 5 min to penetrate cells, followed by irradiation of green LEDs (UltraFire Green Hunting Flashlight, 650 Lumens, Single Mode, 520-535 nm Wavelength) with the dish cover open for 5 min. The cells were then further incubated at 37 ºC, 5% CO_2 in the dark for 20 min. For control group, similarly, the HeLa cells were pretreated by 2 mL of eosin Y (20 μM) in DMEM. Afterwards, the medium was discarded, followed by administration of 3 mL DMEM stock solution with the hood light off (dark conditions). The cells were immediately put in the dark at 37 ºC, 5% CO_2 for 20 min. After incubation, the media was removed, and the cells were washed 2 times using HBSS. Finally, 1 mL HBSS was added, and the cells were detached from their culture dish using a plastic cell scraper. The cells were counted before the lipid extraction (usually 2×10^6 cells/mL). Lipid was extracted from the cell suspension using the Bligh and Dyer method[13]. Briefly, the resuspended cell pellet was transferred to a 10 mL glass vial, 3.75 mL of $CHCl_3$/MeOH (1/2) was added to it, and the sample was vortexed for 10 min. Subsequently, 1.25 mL of $CHCl_3$ was added and the vial was vortexed again for 1 min. Finally, 1.25 mL of distilled water was added to the sample and the vial was vortexed again for 1 min. Afterwards, the mixture was centrifuged at 1000 rpm on a table-top centrifuge for 5 min at room temperature to give a two-phase system. The organic layer of the extraction was collected, and solvent removed by rotary evaporation. The residue was dissolved in 1 mL MeOH/$CHCl_3$ (9:1) and analyzed by HPLC-HRMS in the dark (Q Exactive Orbitrap mass spectrometer (Thermo Fisher Scientific, Waltham, Massachusetts, USA)). Quantification of ceramide-d_{27} (**3n**) was performed in triplicates using the software Tracefinder™ (ThermoScientific) and previously synthesized **3n** as an external standard.

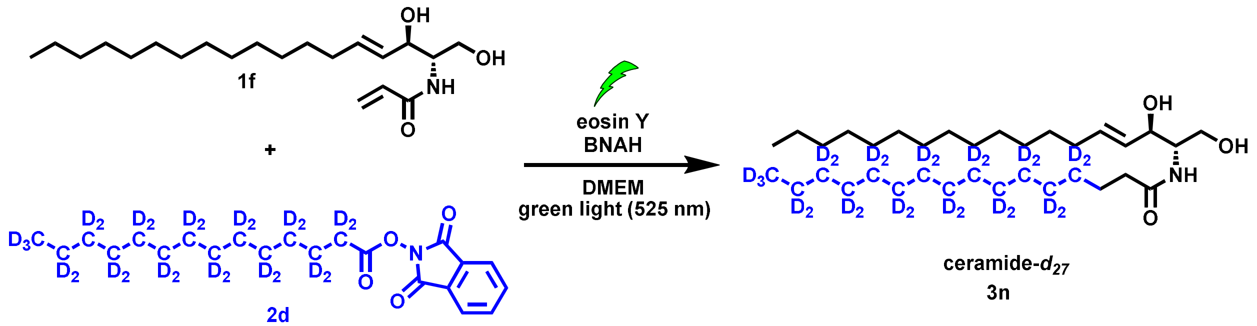

ceramide-d_{27}
3n

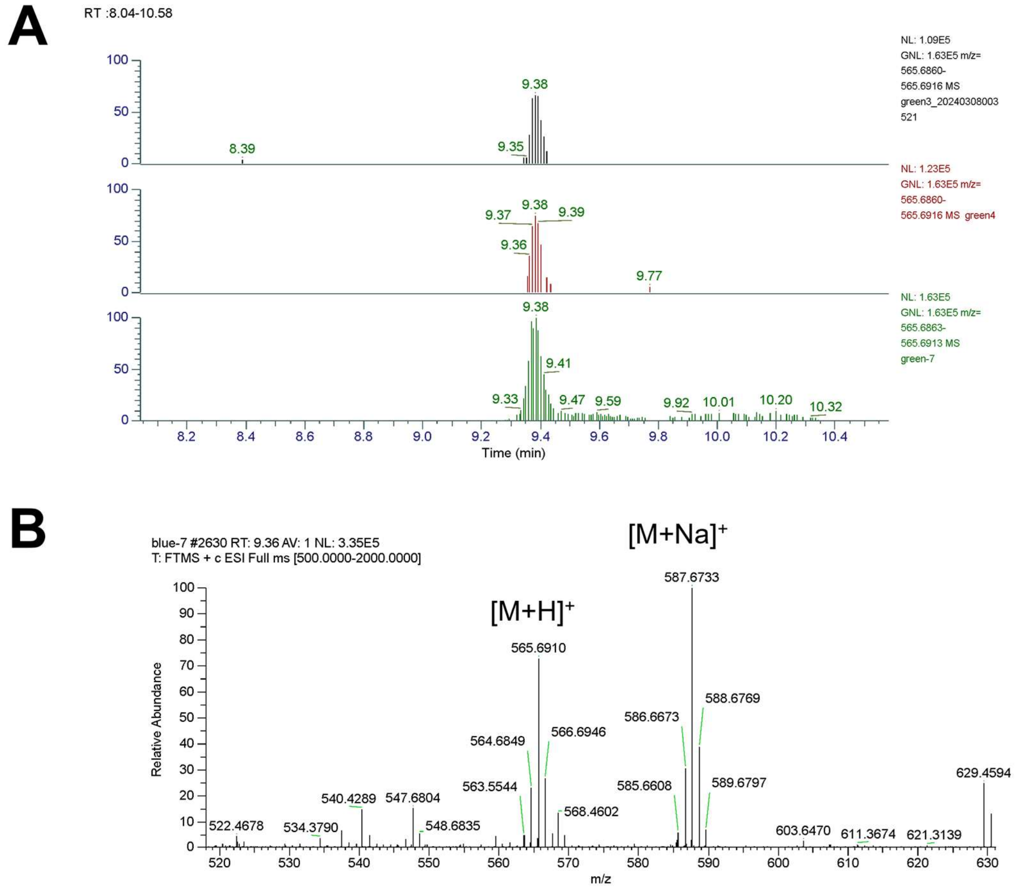

Supplementary Fig. 24. (A) Extracted ion chromatograms of the isotopic ceramide **3n** from three independent cell extraction assays. (B) Representative HRMS spectrum of the isotopic ceramide **3n** from the cell extraction.

18. Cell proliferation assay

Stock solution preparation: For stock solution A, 20 µL of compound **1f** (10 mM in $CHCl_3$), 40 µL of NHPI ester **2a** (10 mM in $CHCl_3$), 60 µL of BNAH (10 mM in $CHCl_3$), 20 µL of C18:1-lyso-PC (10 mM in $CHCl_3$), and 1 µL of eosin Y (10 mM in MeOH) were mixed in a 2-dram amber vial. For stock solution B, 40 µL of NHPI ester **2a** (10 mM in $CHCl_3$), 60 µL of BNAH (10 mM in $CHCl_3$), 20 µL of C18:1-lyso-PC (10 mM in $CHCl_3$), and 1 µL of eosin Y (10 mM in MeOH) were mixed in a 2-dram amber vial. The solvent was evaporated under a gentle stream of N_2, and the content was redissolved in 200 µL of CH_2Cl_2. The CH_2Cl_2 was evaporated again, while carefully rotating the vial to obtain a thin lipid film. The film was hydrated with 4 mL phenol red free DMEM and the suspension was sonicated for 1 min until a homogeneous lipid dispersion was formed. The final concentration was 50 µM for lysolipid **1f**, 100 µM for NHPI ester **2a**, 150 µM for BNAH, 50 µM for C18:1-lyso-PC, and 2.5 µM for eosin Y. For cell treatment the stock

solutions were freshly prepared before each experiment. A 10 mM solution of C16 ceramide (**3h**) in *n*-BuOH was used directly for cell treatment as the positive control.

HeLa cells were plated in a 96-well plate at a density of 10,000 to 20,000 cells/well. After 24 h of incubation at 37 °C, 5% CO_2, the medium was discarded. For the "vehicle" control group, 100 µL phenol red free DMEM was added to the cells. For positive control, 100 µL DMEM containing 50 µM C16 ceramide was added to the cells. For the "treated + light" group, the HeLa cells were treated by 100 µL stock solution A and stood in the dark incubator for 5 min, followed by irradiation by green LEDs (UltraFire Green Hunting Flashlight, 650 Lumens, Single Mode, 520-535 nm Wavelength) with cover open for 5 min. For the "treated + dark" control group, HeLa cells were treated by 100 µL stock solution A with the hood light off and then immediately incubated in the dark to avoid any undesired illumination. For the "treated no ester + light" group, the HeLa cells were treated with 100 µL of stock solution B and then irradiated by green light for 5 min after incubation in the dark for 5 min. For the "light only" control group, the HeLa cells were treated with 100 µL DMEM medium, and irradiated under green LEDs for 5 min. All the six groups were incubated at 37 °C, 5% CO_2 for 24 h after treatment. After 24 h, 10 µL of Cell Counting Kit-8 (CCK-8, APExBIO, Cat. No. K1018) solution was added to each well of the plate. Care was taken not to introduce air bubbles into the wells, since air bubbles can interfere with absorbance measurements. The plate was incubated for 1 h in the cell incubator. The absorbance was measured at 450 nm using a Safire II plate reader (Tecan). The background absorbance of the CCK-8 reagent in media was subtracted and cell viability was reported as a percentage of the viability of the vehicle control. All conditions were tested in triplicate and plotted with error bars representing standard deviation.

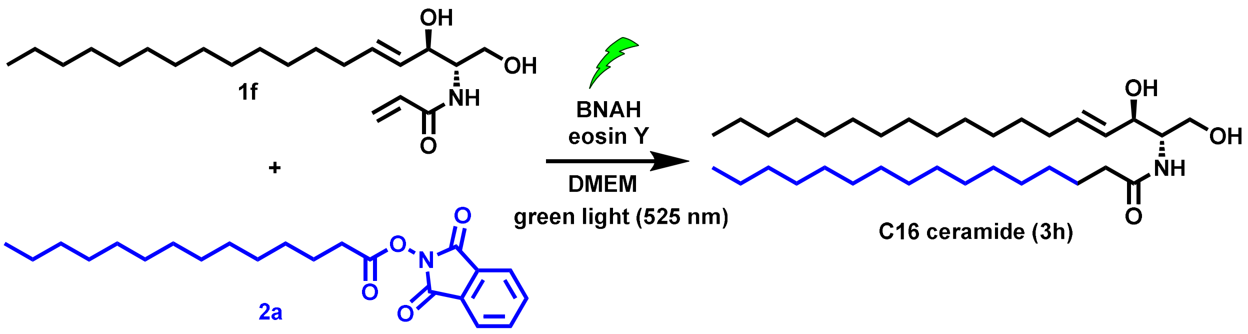

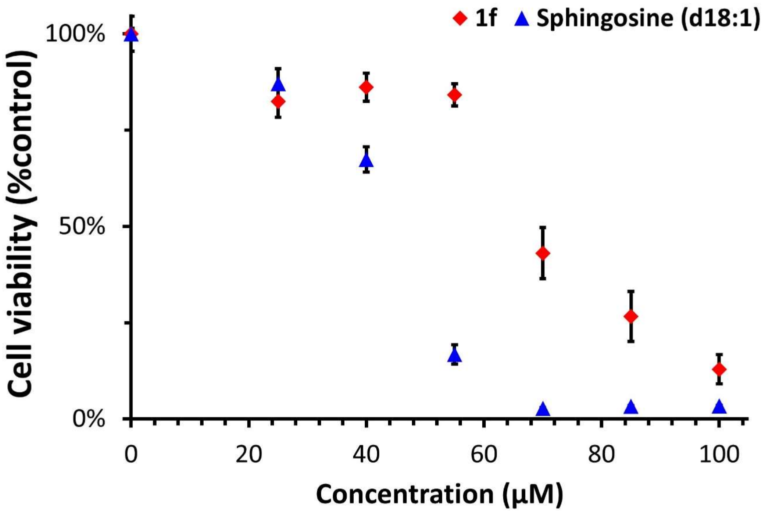

Supplementary Fig. 25. Cytotoxicity of sphingosine (d18:1) and **1f**. The estimated IC_{50} for sphingosine (d18:1) is 45 μM. The estimated IC_{50} for lysolipid **1f** is 67 μM. The data points are presented as means ± SD (n = 3 biologically independent samples).

19. Western blot assay

Stock solution preparation: 20 μL of **1g** (10 mM in $CHCl_3$), 40 μL of **2b** (10 mM in $CHCl_3$), 60 μL of BNAH (10 mM in $CHCl_3$), and 1 μL of eosin Y (10 mM in MeOH) were mixed in a 2-dram amber vial. The solvent was evaporated under a gentle stream of N_2 and the resulting mixture was redissolved in 100 μL of CH_2Cl_2. The CH_2Cl_2 was evaporated again, while carefully rotating the vial to obtain a thin lipid film. The film was hydrated with 8 mL phenol red free DMEM and the suspension was sonicated for 1 min until a homogeneous lipid dispersion with a final concentration of 25 μM for **1g**, 50 μM for **2b**, 75 μM for BNAH, and 1.25 μM for eosin Y. For cell treatment the stock solutions were freshly prepared before each experiment.

HeLa cells, maintained in DMEM (10% FBS, 1% penicillin/streptomycin), were plated in a 33 mm dish at a density of 50,000 cells/dish. After 24 h of incubation at 37 °C, 5% CO_2, the cell medium was discarded, and the cells were treated as follows. For the control group, HeLa cells were incubated in DMEM medium without any additives. For the "treatment + light" group, HeLa cells were treated with 1 mL freshly prepared stock solution (see above), followed by irradiation with green LEDs. The dish cover was removed, and the cells were irradiated for 5 min. For the "treatment + no-light" control group, HeLa cells were also treated with 1 mL of the freshly prepared DMEM treatment stock solution. The cell hood light was kept off and the cells were immediately incubated in the dark to minimize light exposure. The treated cells were kept at 37 °C, 5% CO_2 for 5 min. For the "inhibitor" control group, HeLa cells were pretreated with 1 μM PKC inhibitor (Gö 6983) for 5 min. Afterwards, the cell medium was removed, and the cells were treated with 1 mL of freshly prepared DMEM treatment stock solution, followed by irradiation with green LEDs for 5 min. As a positive control, HeLa cells were incubated with 1 mL PKC agonist (0.2 μM

PDBu) for 10 min. After the indicated treatment times, the cells were washed 2 times with HBSS before cell lysis for the following western blot assay.

Cell lysates were prepared in lysis buffer (50 mM sodium phosphate, 1 mM sodium pyrophosphate, 20 mM sodium fluoride, 2mM EDTA, 1% Triton X-100, pH = 7.4; supplemented with Halt™ protease inhibitor cocktail and Roche PhosSTOP™) directly after cell treatment. Cells were detached with a cell scraper and sonicated with a tip sonicator (Fisherbrand™ Sonic Dismembrator, 2 bursts of 15 s, 50% amplitude). The resulting cell lysates were separated on a Mini-PROTEAN® TGX™ gel (4-20%) and subsequently transferred to a PVDF membrane using the Trans-Blot® Turbo™ system. Membranes were blocked for 1 h at rt (5% BSA in TBS-T) after which they were incubated with primary antibody: Anti-Actin (8H10D10) antibody (1:1000) (#3700, Cell Signaling Technology), Anti-Phospho-(Ser) PKC substrate antibody (1:1000) (#2261, Cell Signaling Technology).

Following incubation overnight at 4 °C, membranes were washed with TBS-T and the corresponding secondary antibody was added for 1 h at rt. After five successive washes with TBS-T, blots were imaged with Thermo Scientific™ SuperSignal™ West Pico PLUS on a ChemiDoc™ XRS+.

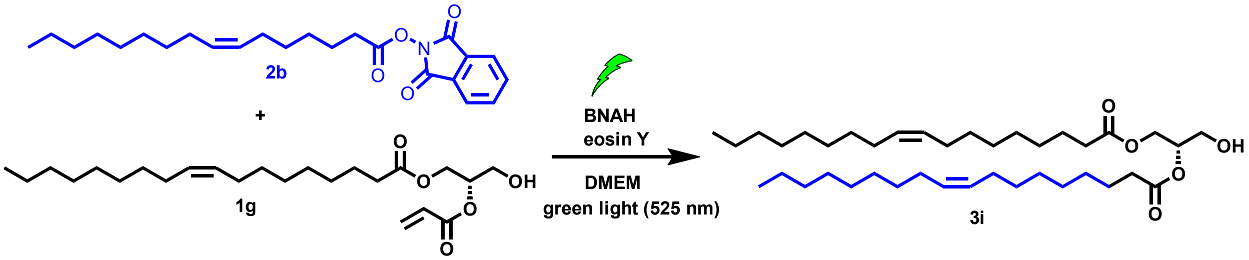

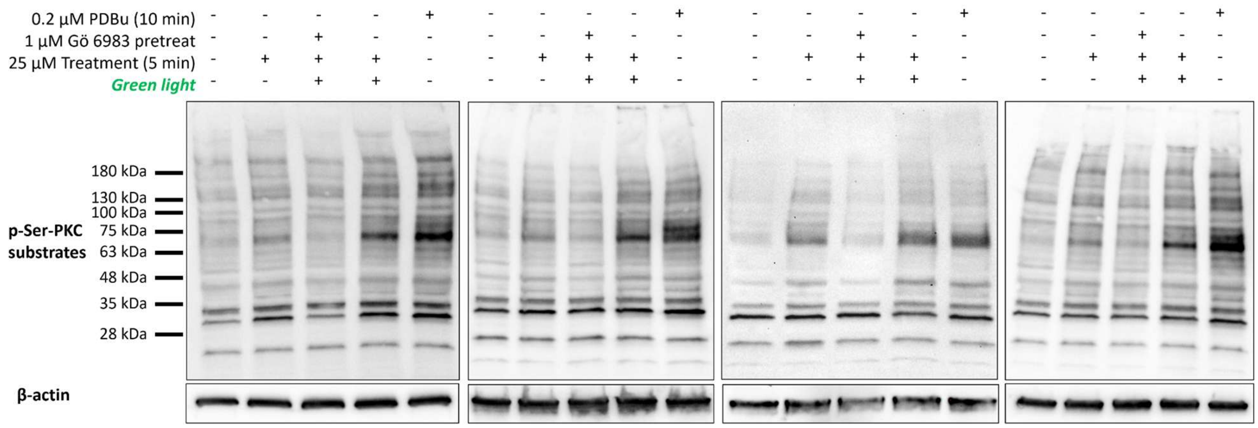

Supplementary Fig. 26. Western blot images of whole-cell lysate from HeLa cells demonstrating that the in situ synthesis of 1-2-dioleoyl-sn-glycerol **3i** induces PKC protein upregulation comparable to that observed when the agonist PDBu was used. Source data are provided as a Source Data file.

20. NMR spectra

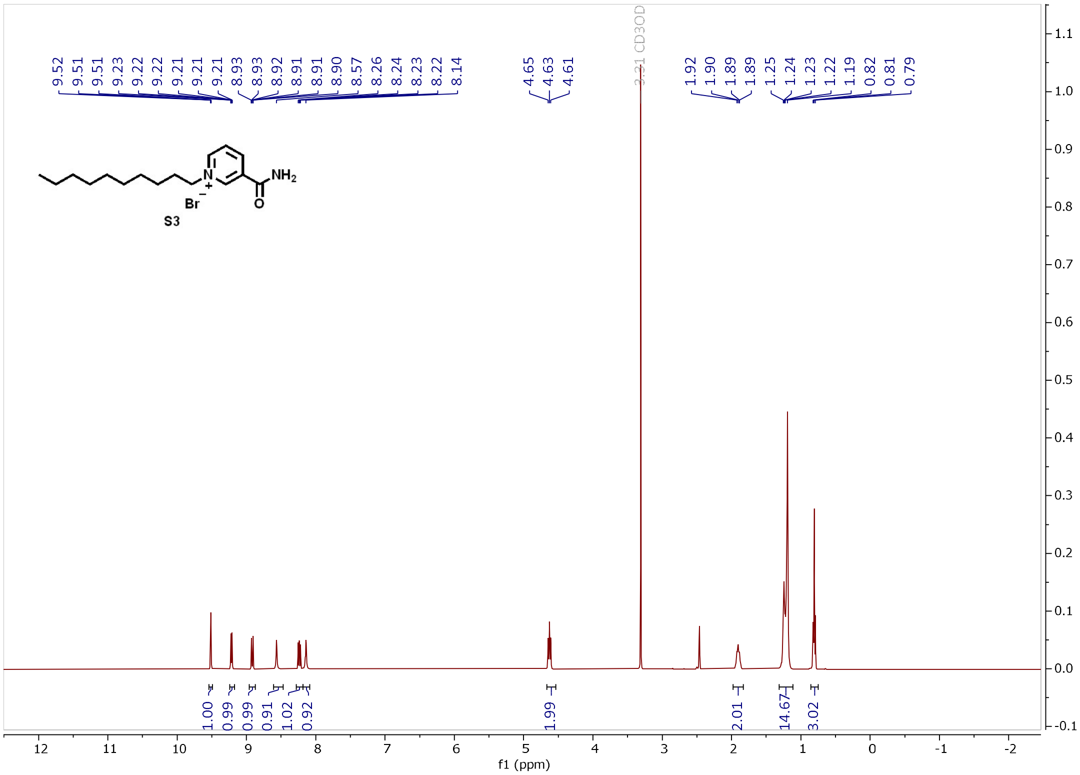

Supplementary Fig. 27. ^{1}H NMR (400 MHz, DMSO-d_6) of compound **S3**.

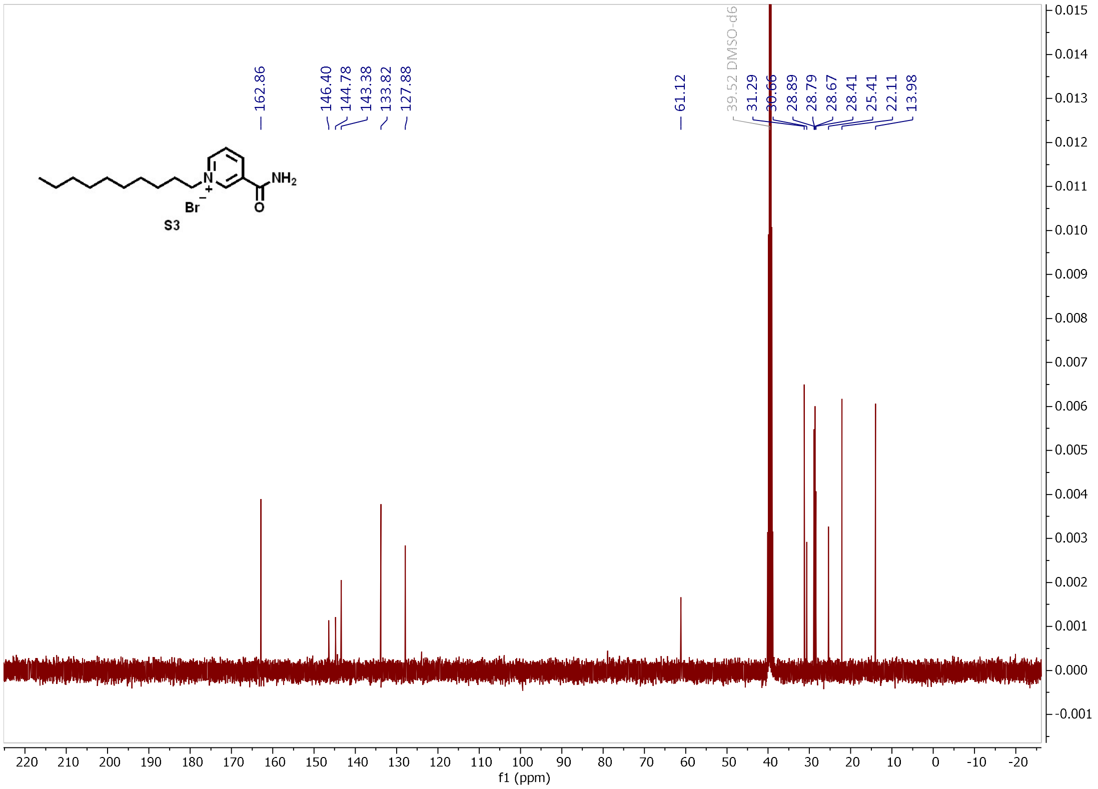

Supplementary Fig. 28. ^{13}C NMR (100 MHz, DMSO-d_6) of compound **S3**.

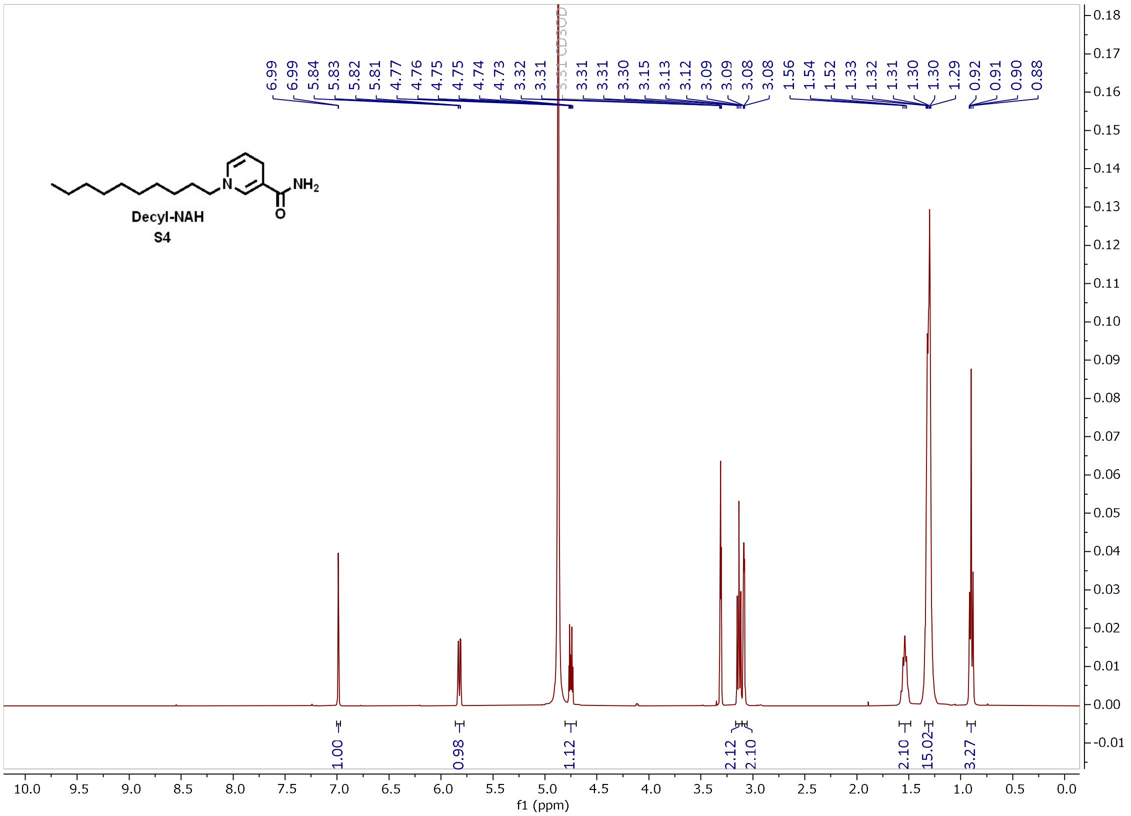

Supplementary Fig. 29. ^{1}H NMR (400 MHz, CD_3OD) of compound **S4**.

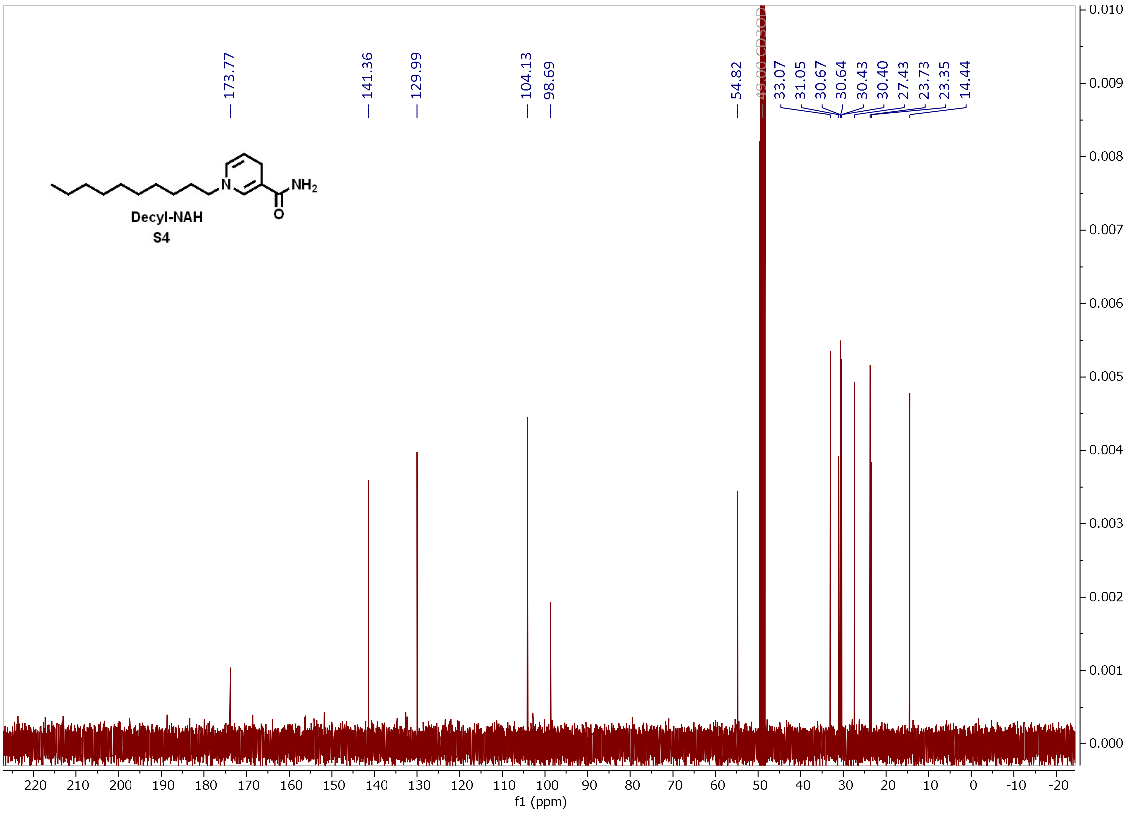

Supplementary Fig. 30. ^{13}C NMR (100 MHz, CD_3OD) of compound **S4**.

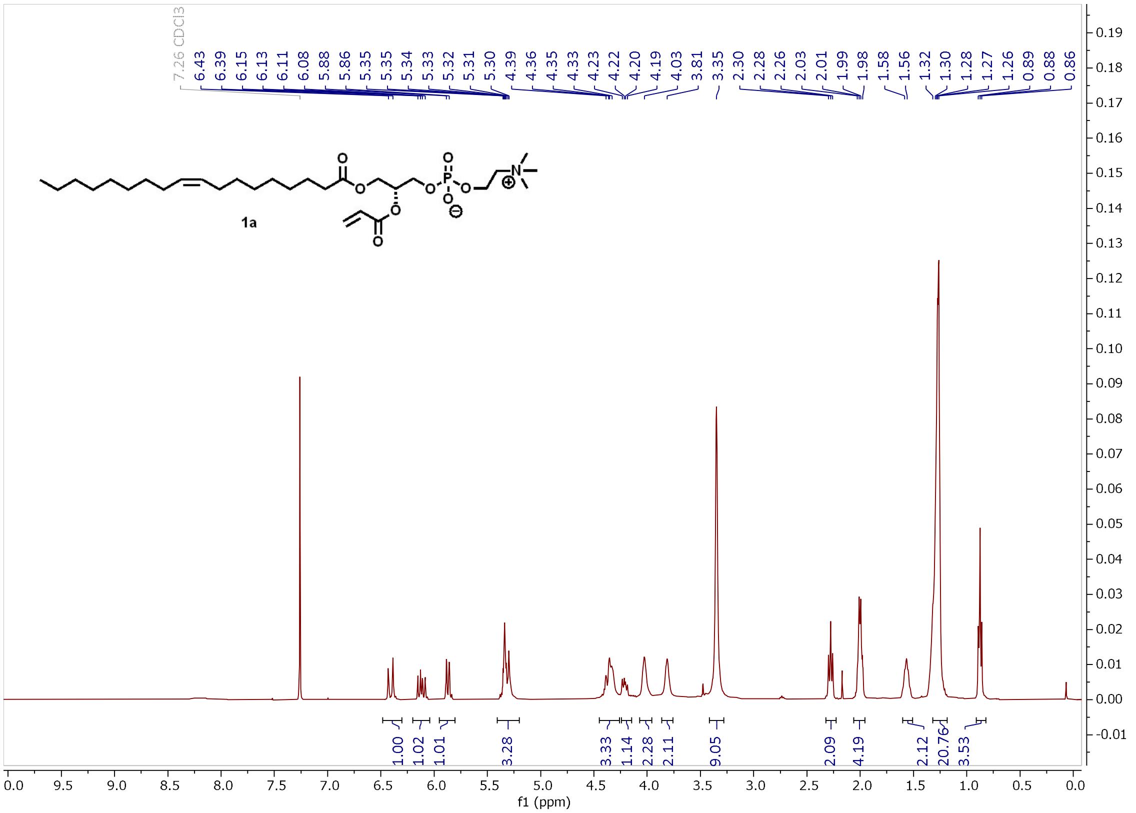

Supplementary Fig. 31. ^{1}H NMR (400 MHz, $CDCl_3$) of compound **1a**.

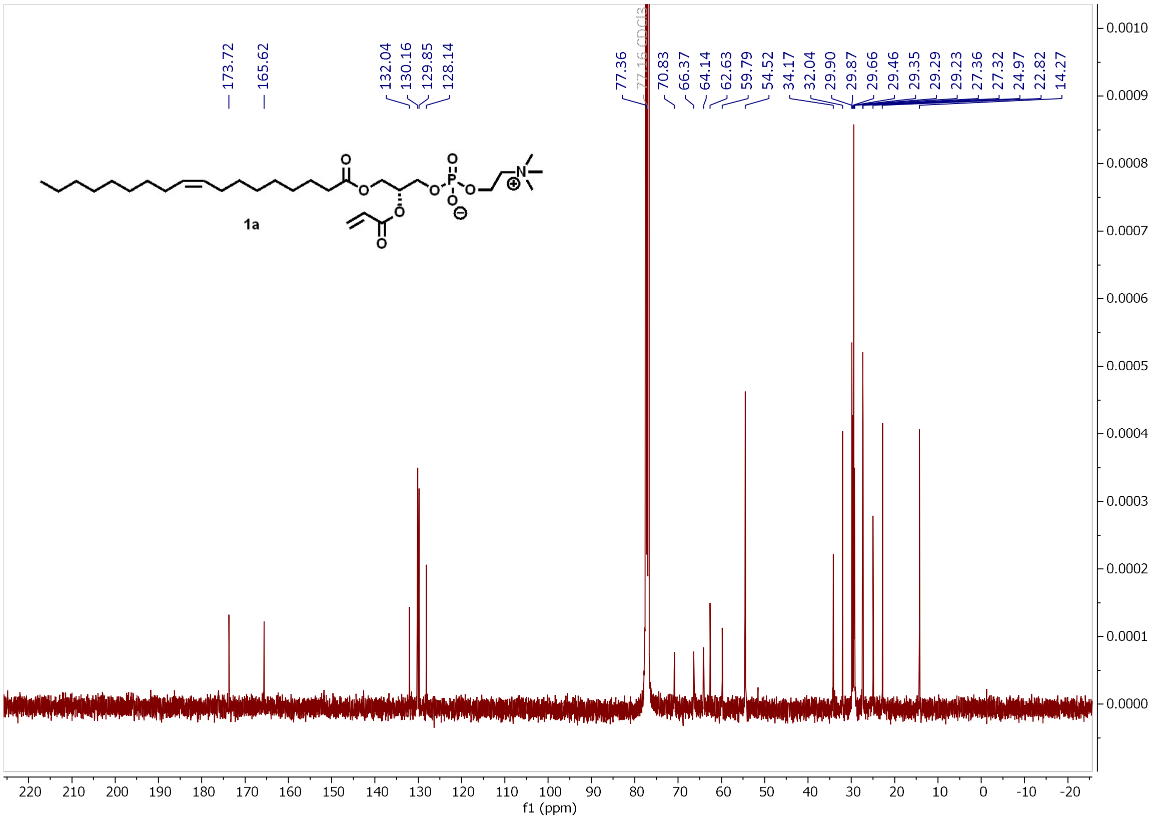

Supplementary Fig. 32. ^{13}C NMR (100 MHz, $CDCl_3$) of compound **1a**.

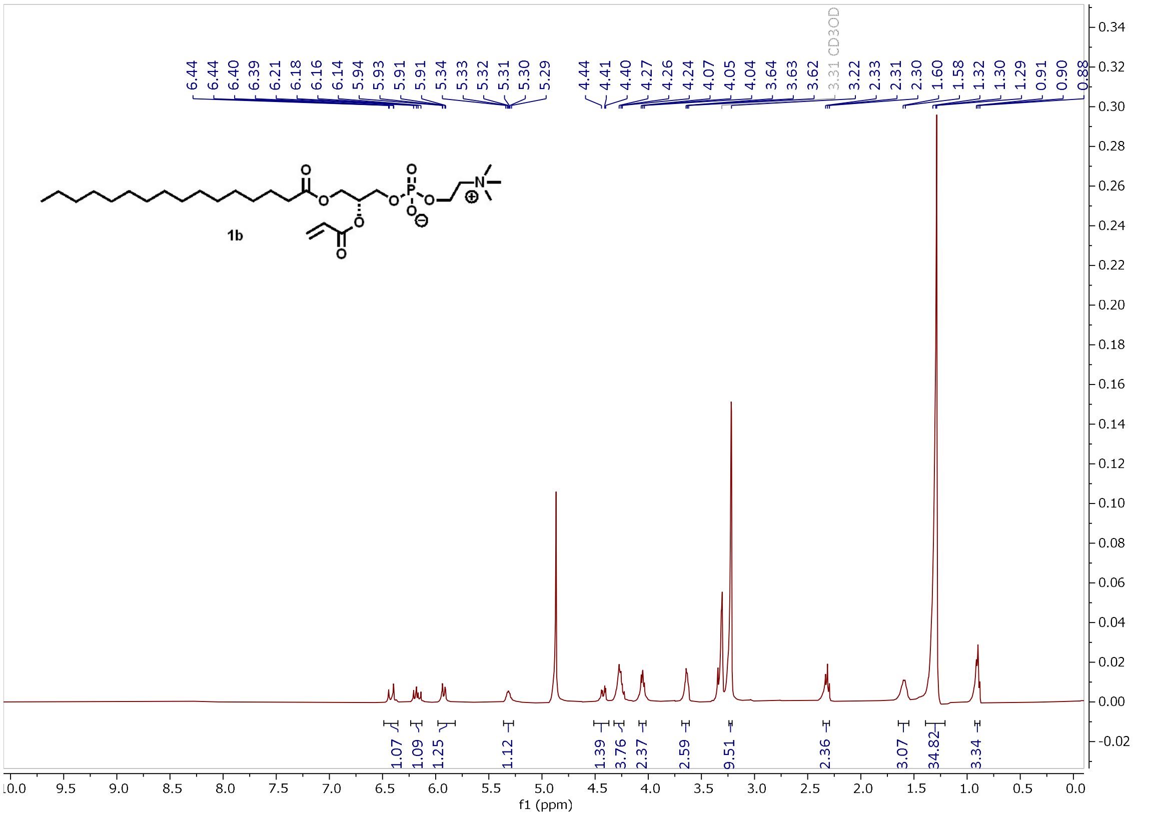

Supplementary Fig. 33. ^{1}H NMR (400 MHz, CD_3OD) of compound **1b**.

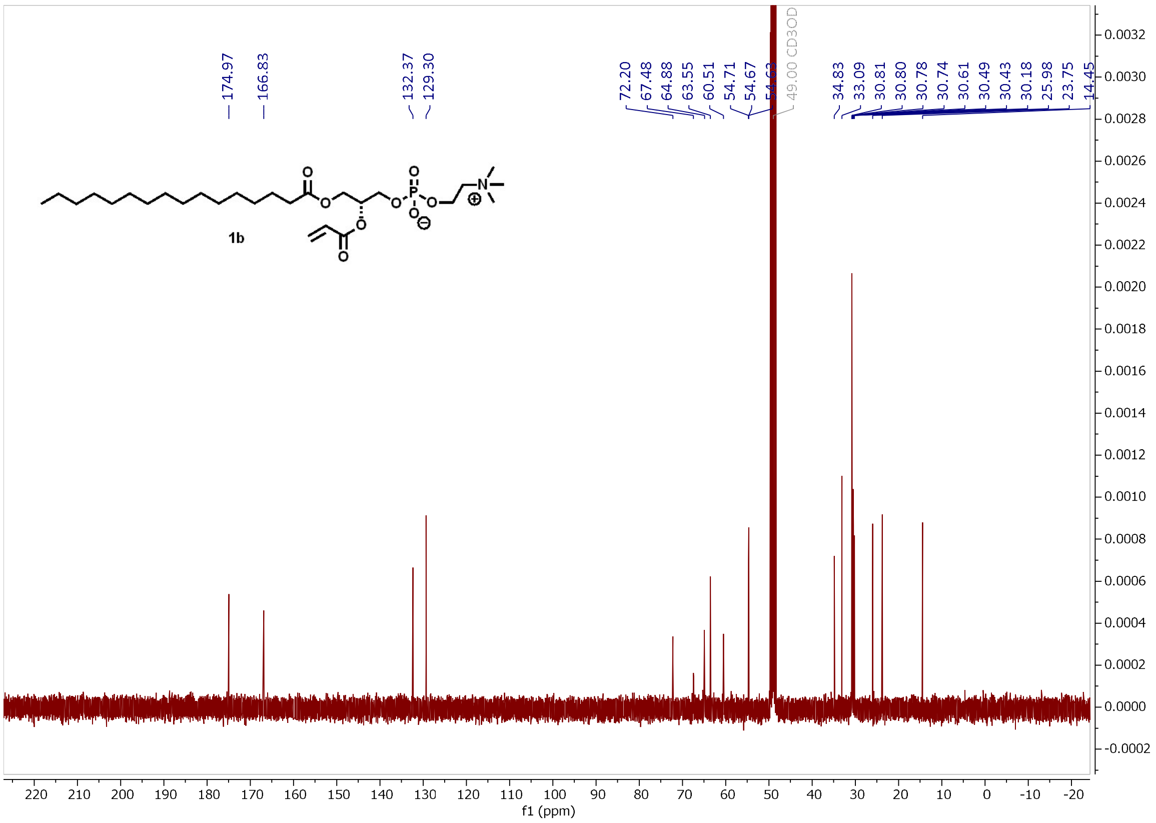

Supplementary Fig. 34. ^{13}C NMR (100 MHz, CD_3OD) of compound **1b**.

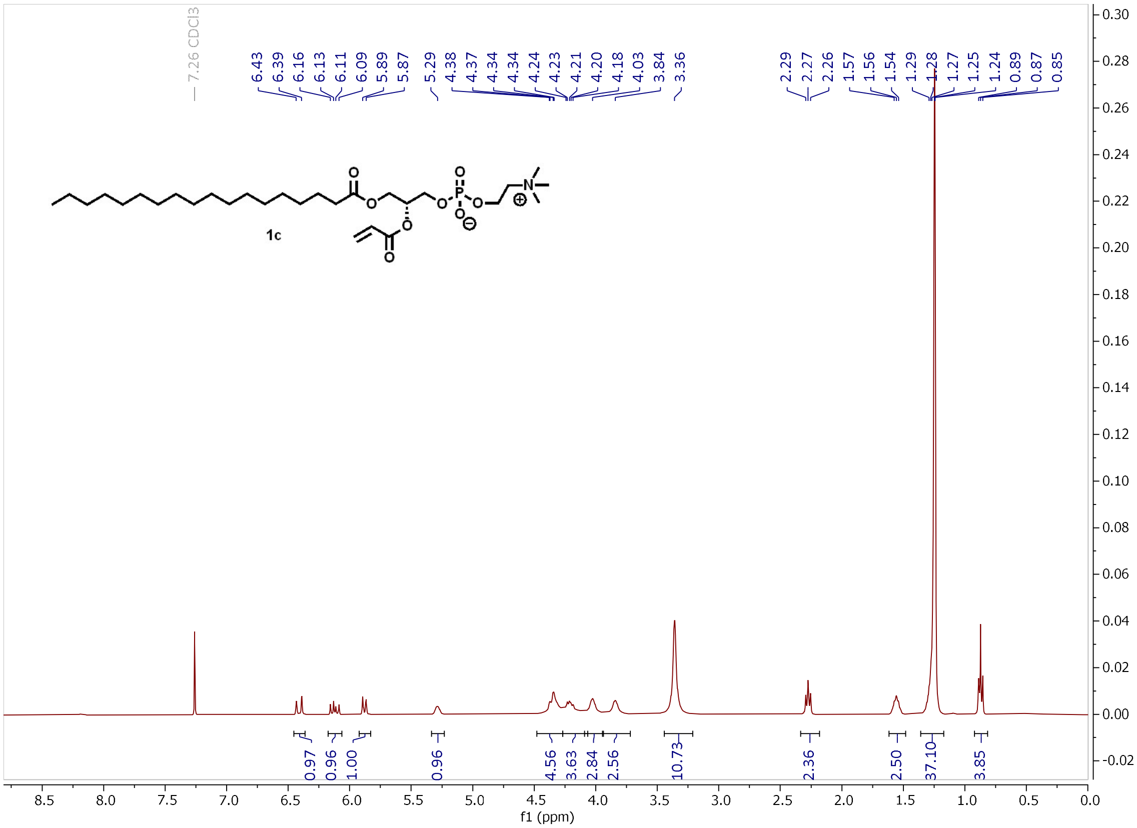

Supplementary Fig. 35. ^{1}H NMR (400 MHz, $CDCl_3$) of compound **1c**.

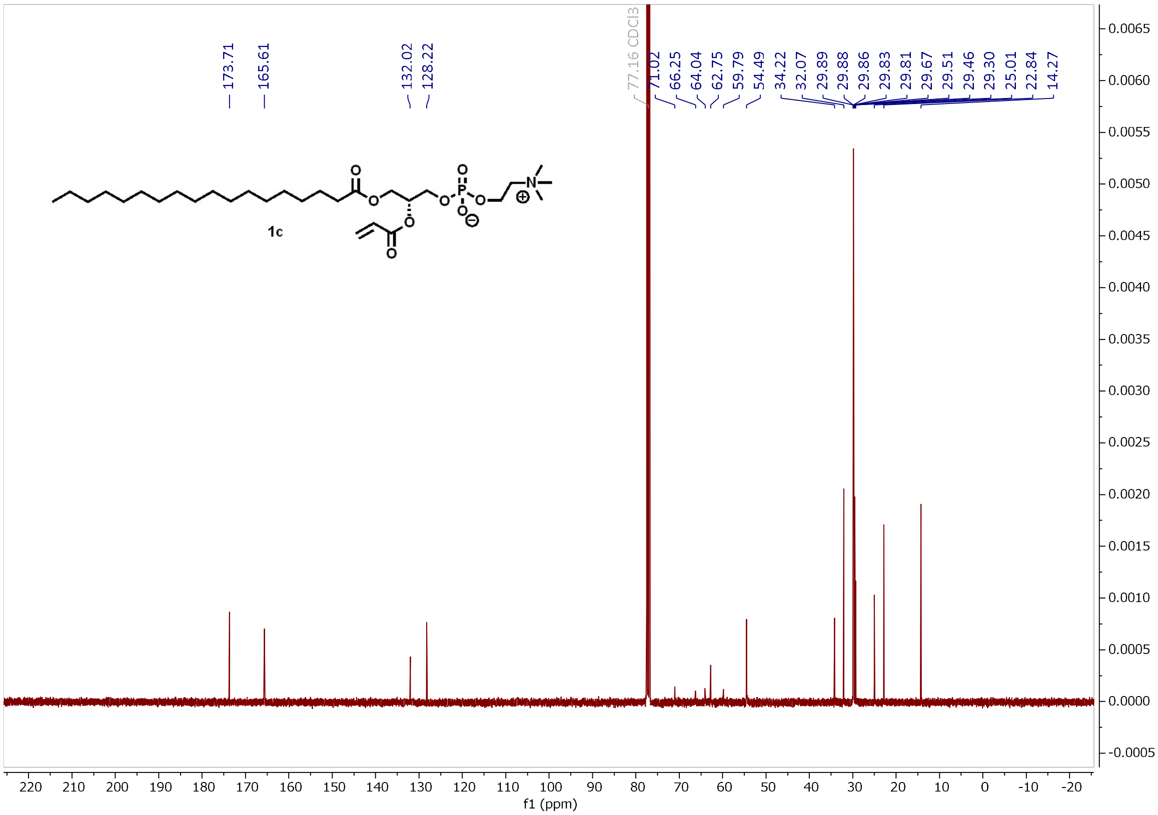

Supplementary Fig. 36. ^{13}C NMR (100 MHz, $CDCl_3$) of compound **1b**.

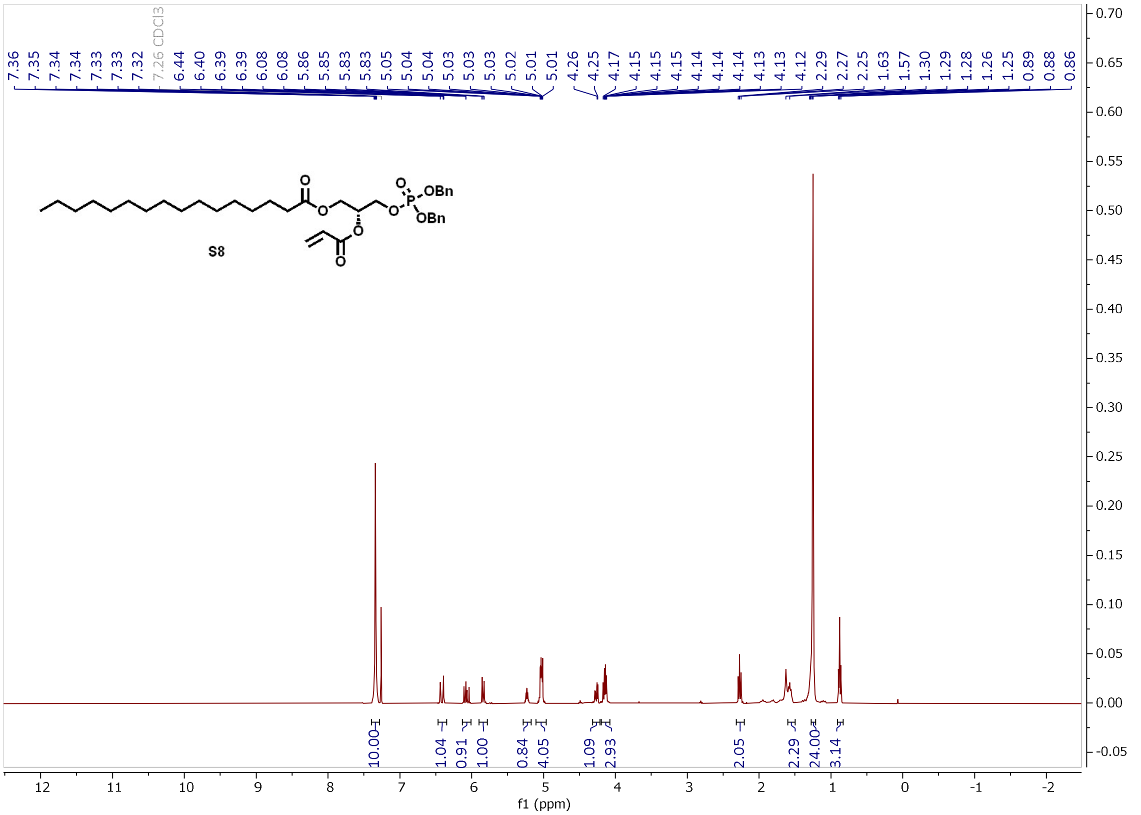

Supplementary Fig. 37. ^{1}H NMR (400 MHz, $CDCl_3$) of compound **S8**.

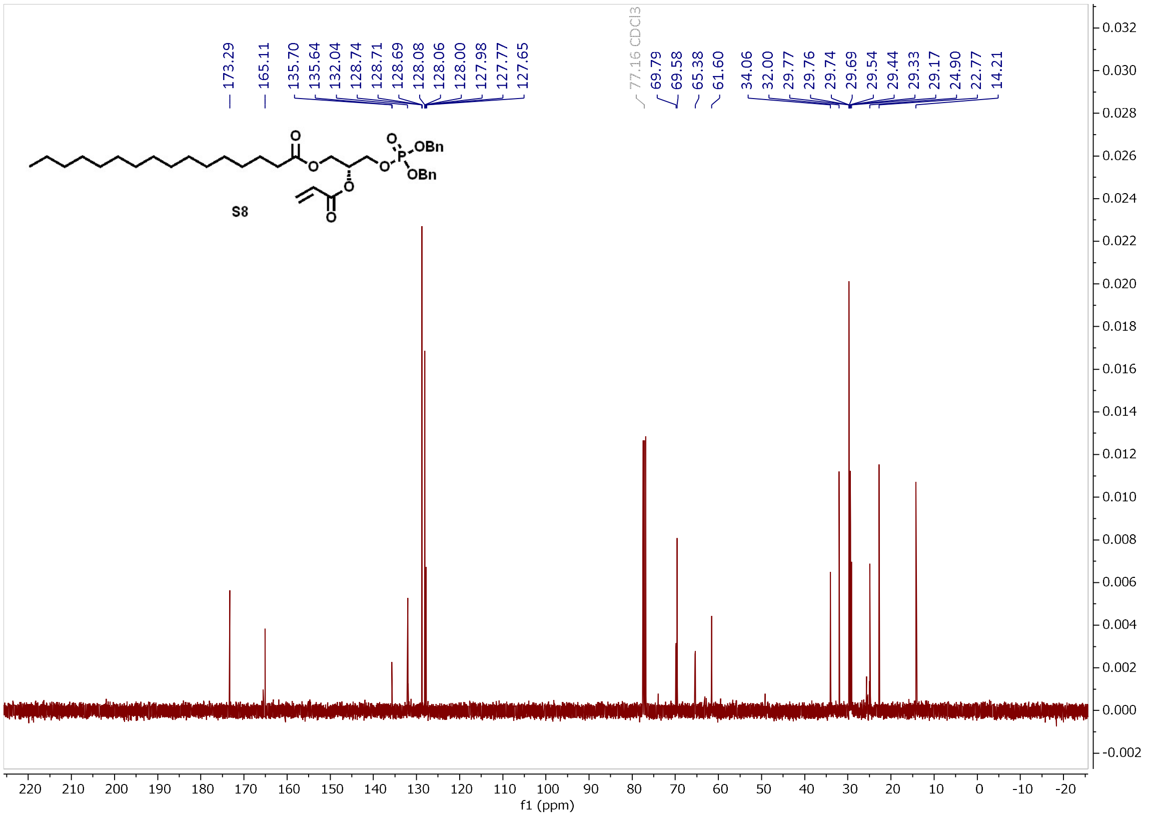

Supplementary Fig. 38. ^{13}C NMR (100 MHz, $CDCl_3$) of compound **S8**.

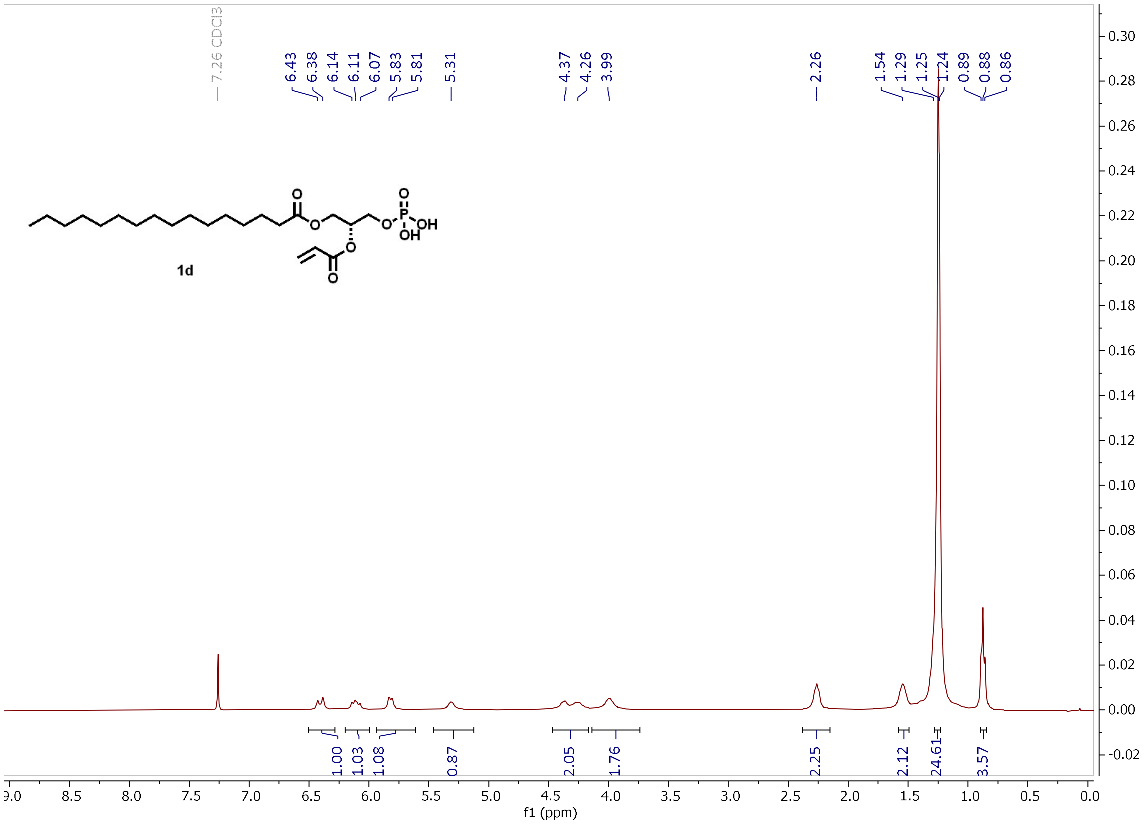

Supplementary Fig. 39. ^{1}H NMR (400 MHz, $CDCl_3$) of compound **1d**.

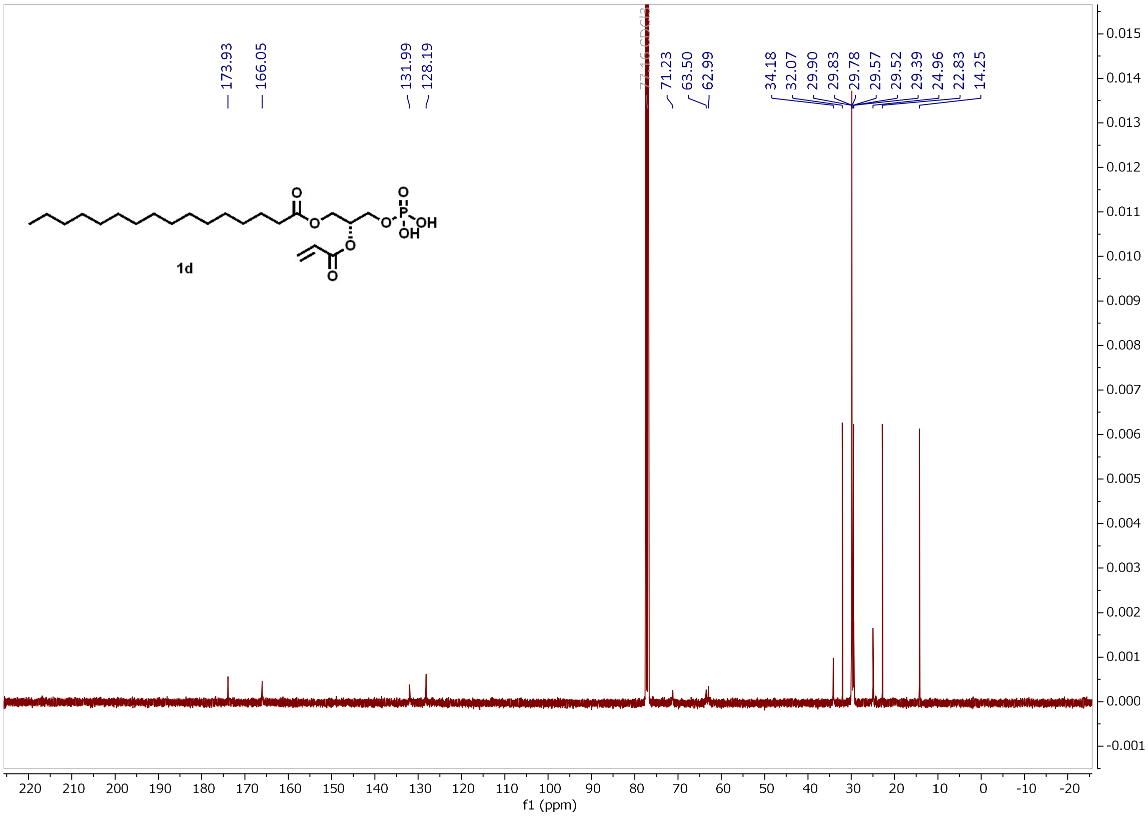

Supplementary Fig. 40. ^{13}C NMR (100 MHz, $CDCl_3$) of compound **1d**.

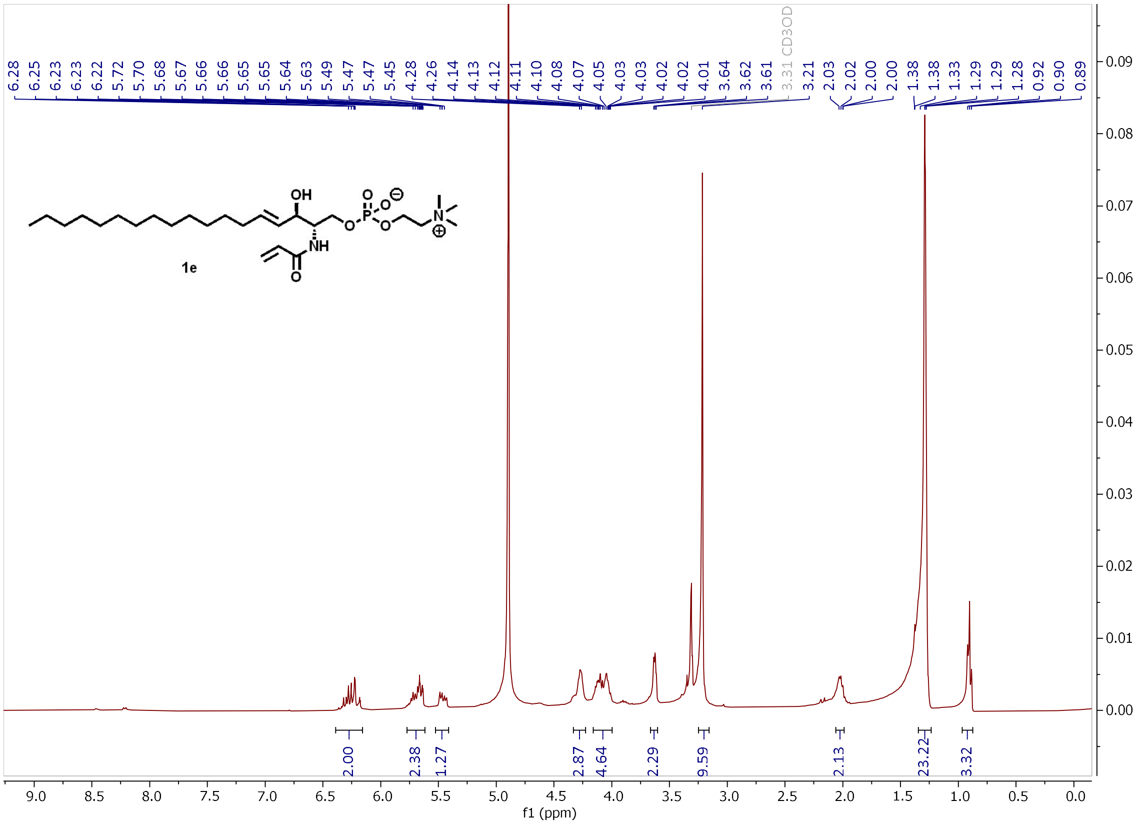

Supplementary Fig. 41. ^{1}H NMR (400 MHz, CD_3OD) of compound **1e**.

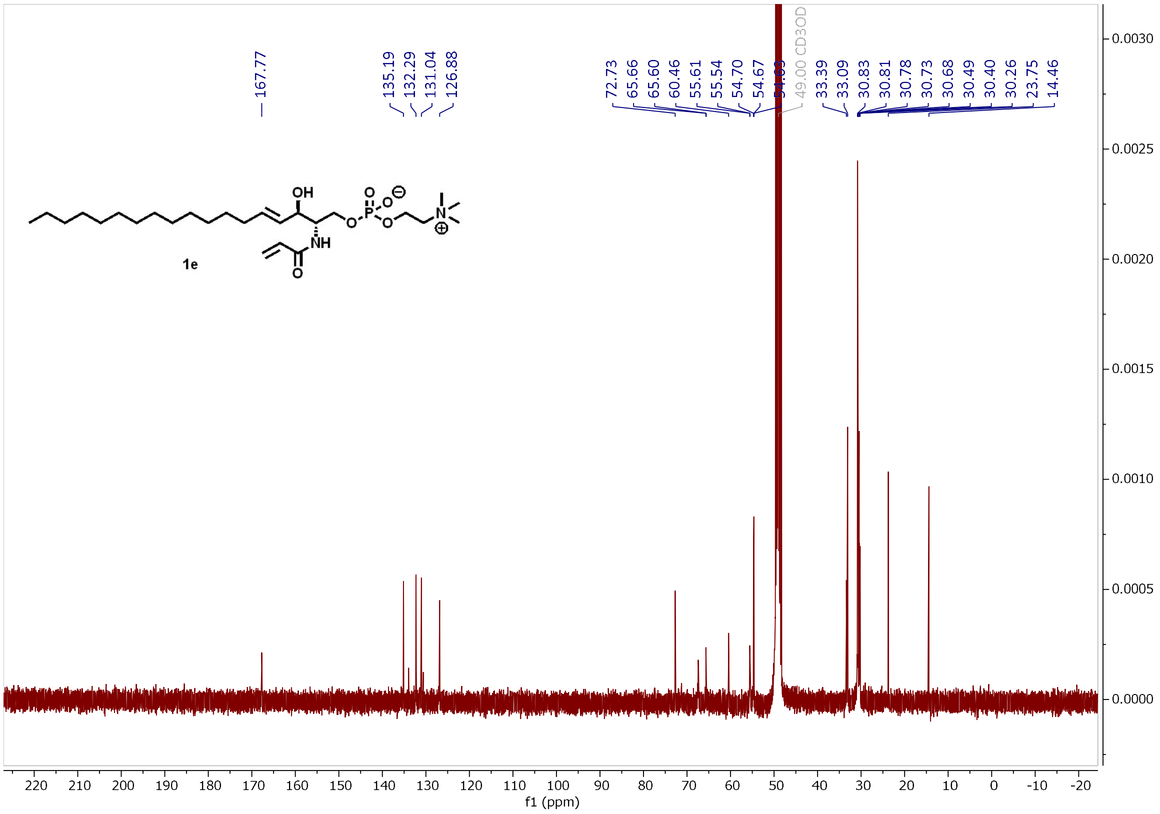

Supplementary Fig. 42. ^{13}C NMR (100 MHz, CD_3OD) of compound **1e**.

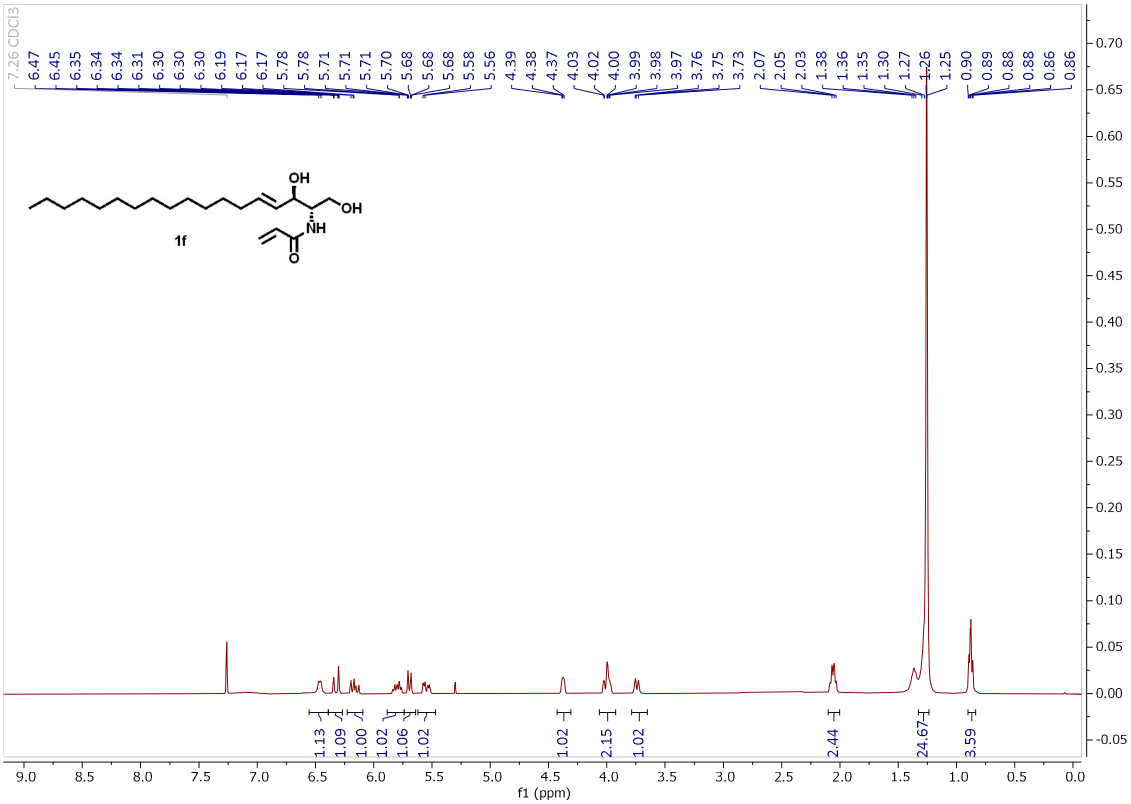

Supplementary Fig. 43. ^{1}H NMR (400 MHz, $CDCl_3$) of compound **1f**.

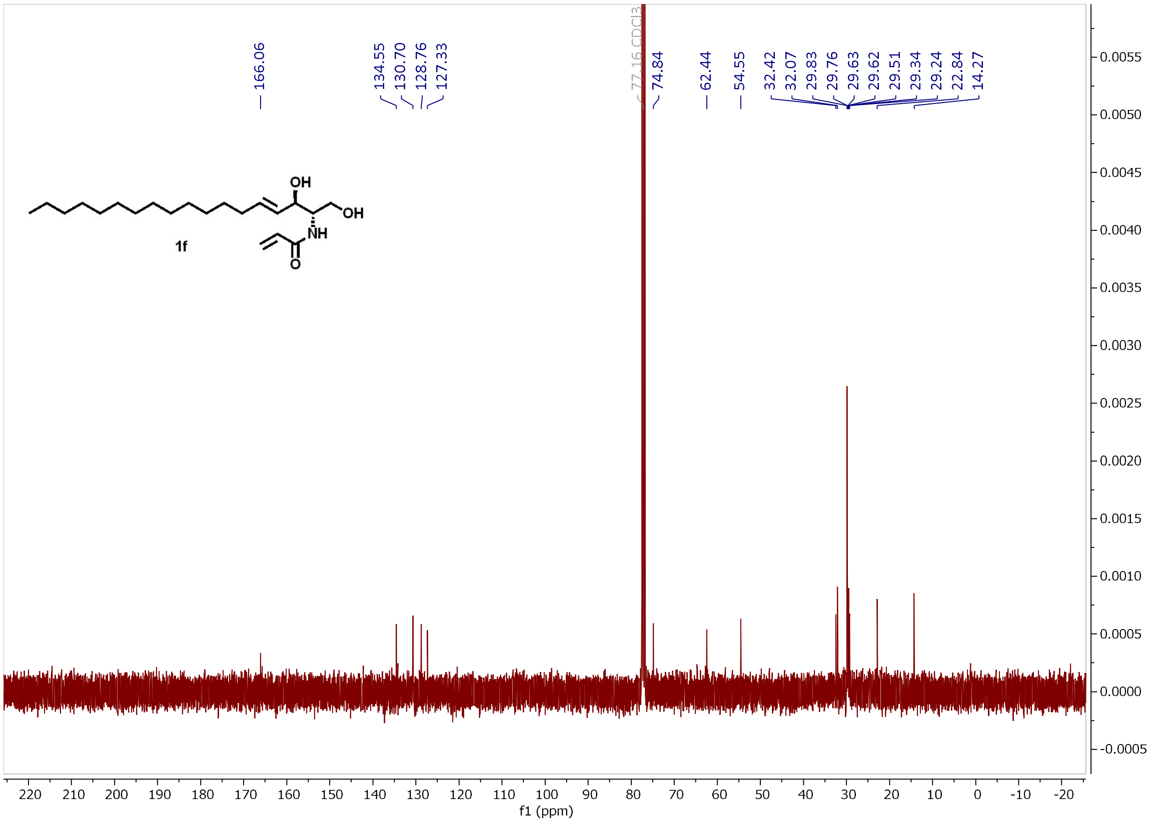

Supplementary Fig. 44. ^{13}C NMR (100 MHz, $CDCl_3$) of compound **1f**.

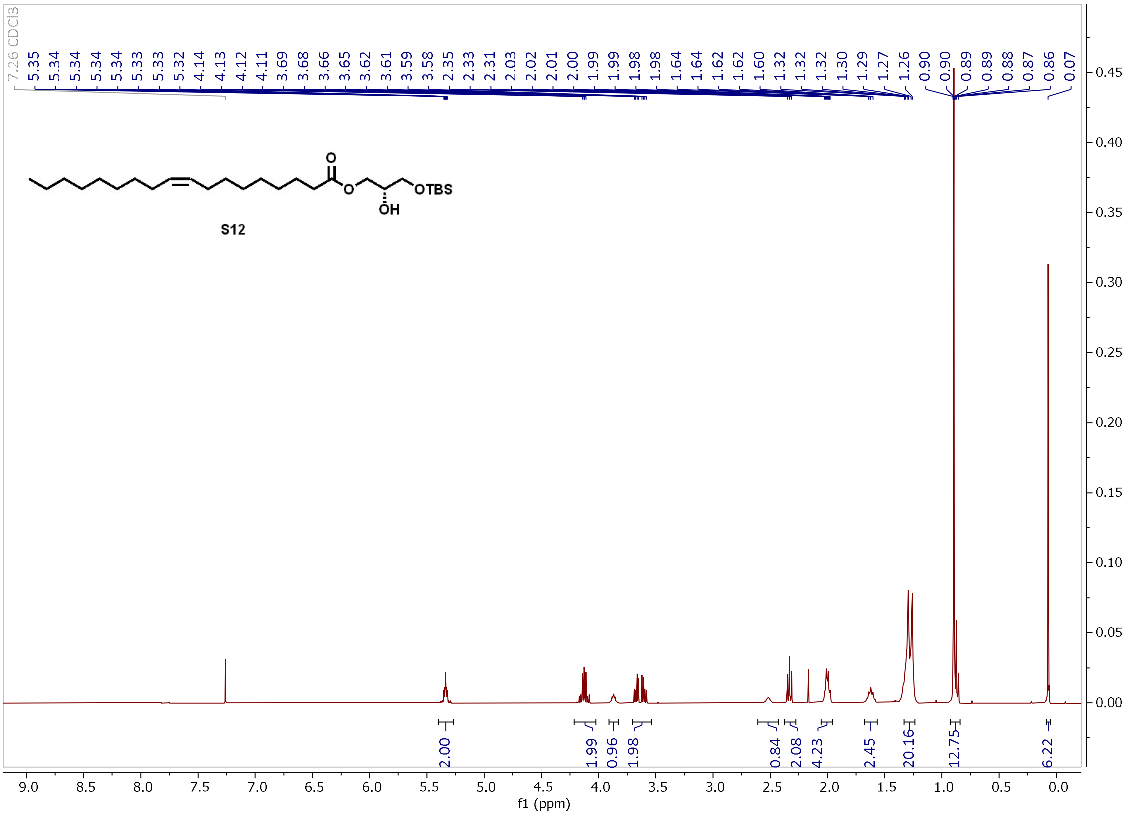

Supplementary Fig. 45. ^{1}H NMR (400 MHz, $CDCl_3$) of compound **S12**

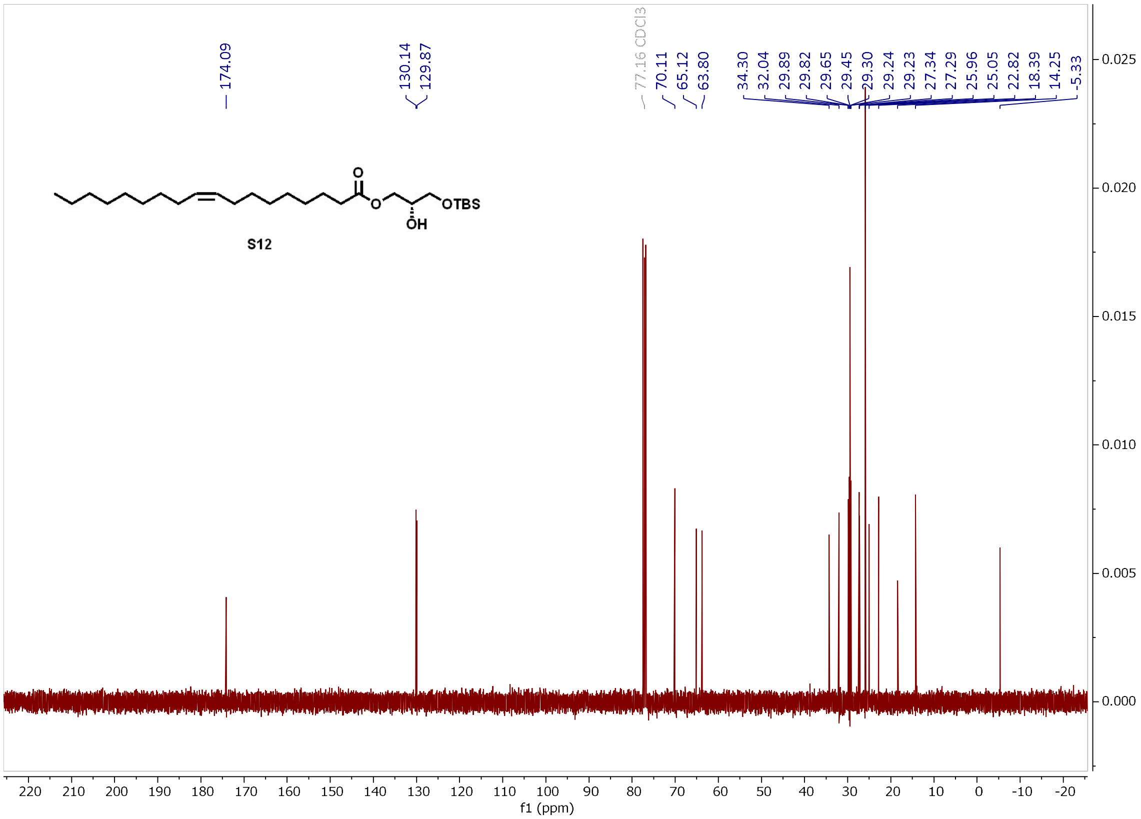

Supplementary Fig. 46. ^{13}C NMR (100 MHz, $CDCl_3$) of compound **S12**.

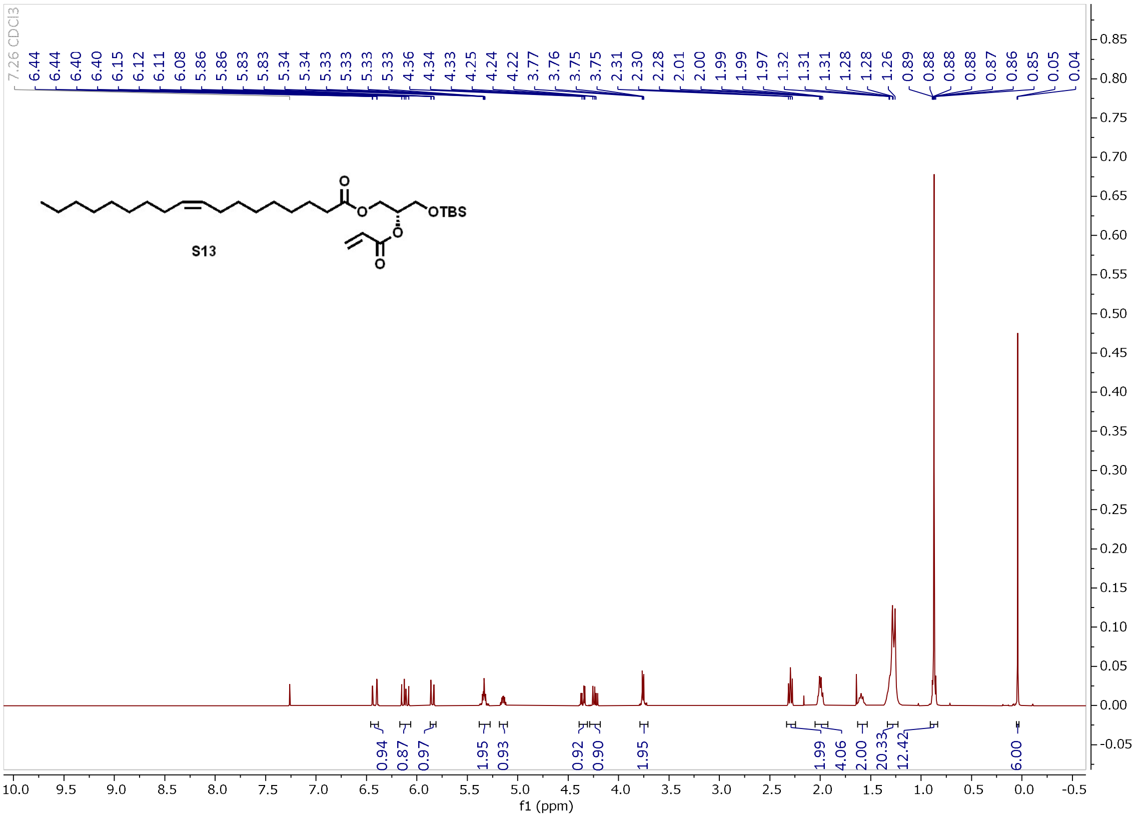

Supplementary Fig. 47. ^{1}H NMR (400 MHz, $CDCl_3$) of compound **S13.**

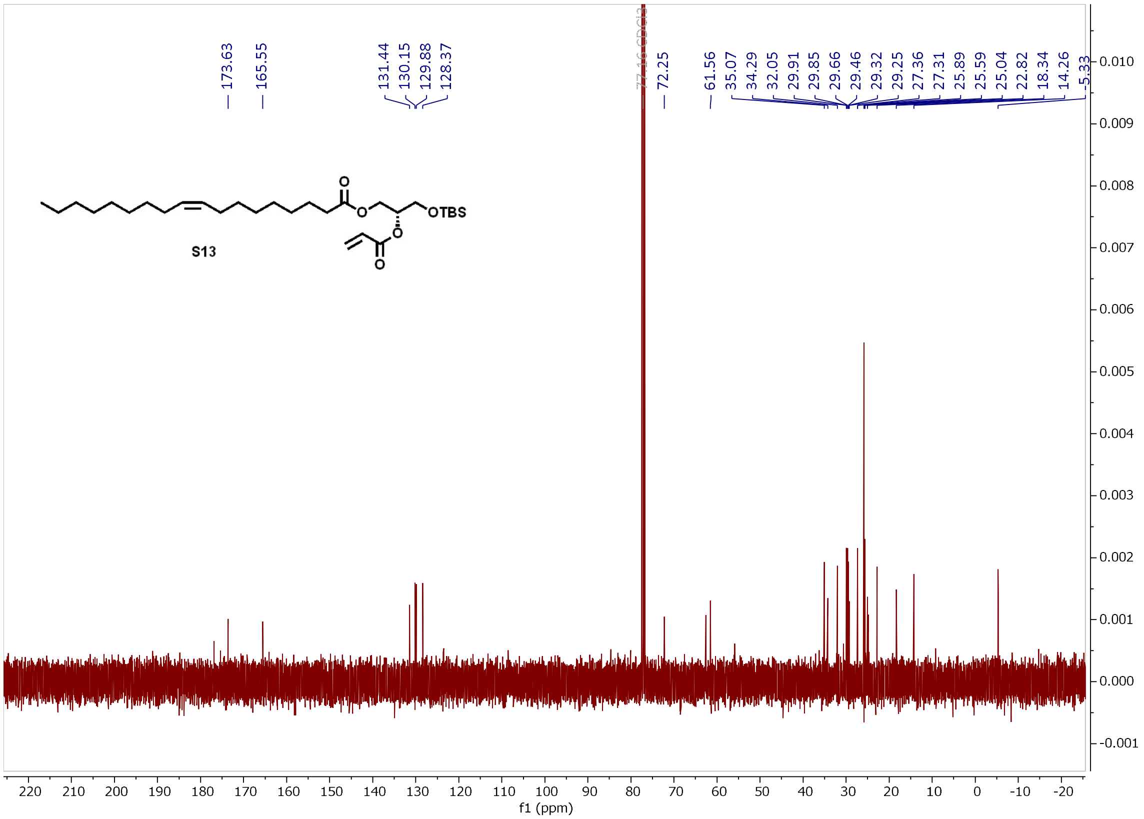

Supplementary Fig. 48. ^{13}C NMR (100 MHz, $CDCl_3$) of compound **S13**.

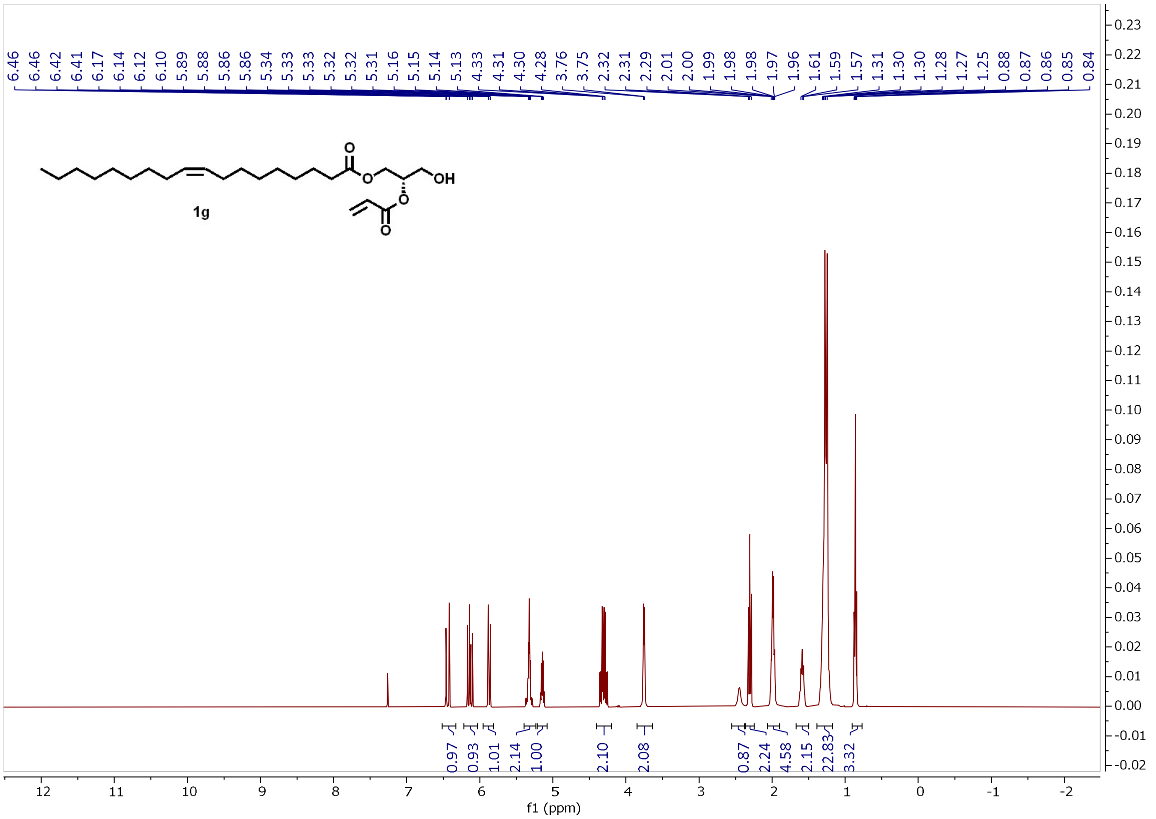

Supplementary Fig. 49. ^{1}H NMR (400 MHz, $CDCl_3$) of compound **1g.**

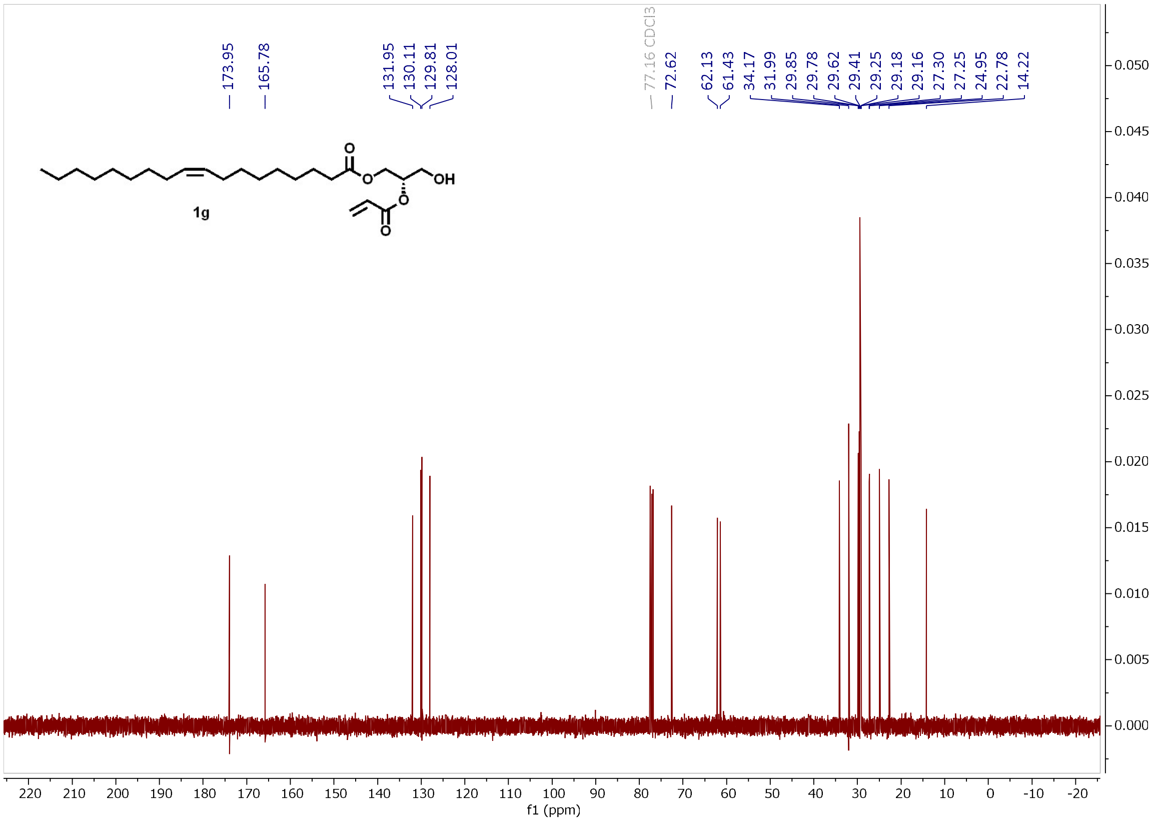

Supplementary Fig. 50. ^{13}C NMR (100 MHz, $CDCl_3$) of compound **1g**.

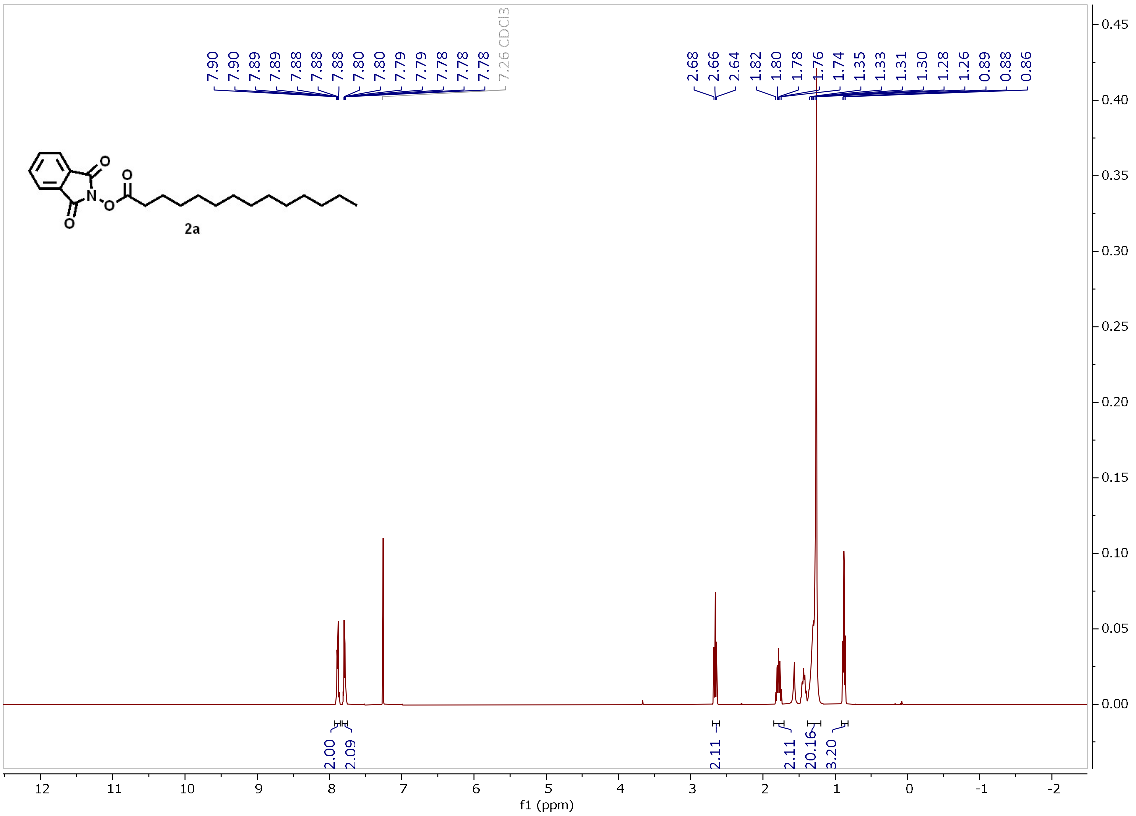

Supplementary Fig. 51. ^{1}H NMR (400 MHz, $CDCl_3$) of compound **2a.**

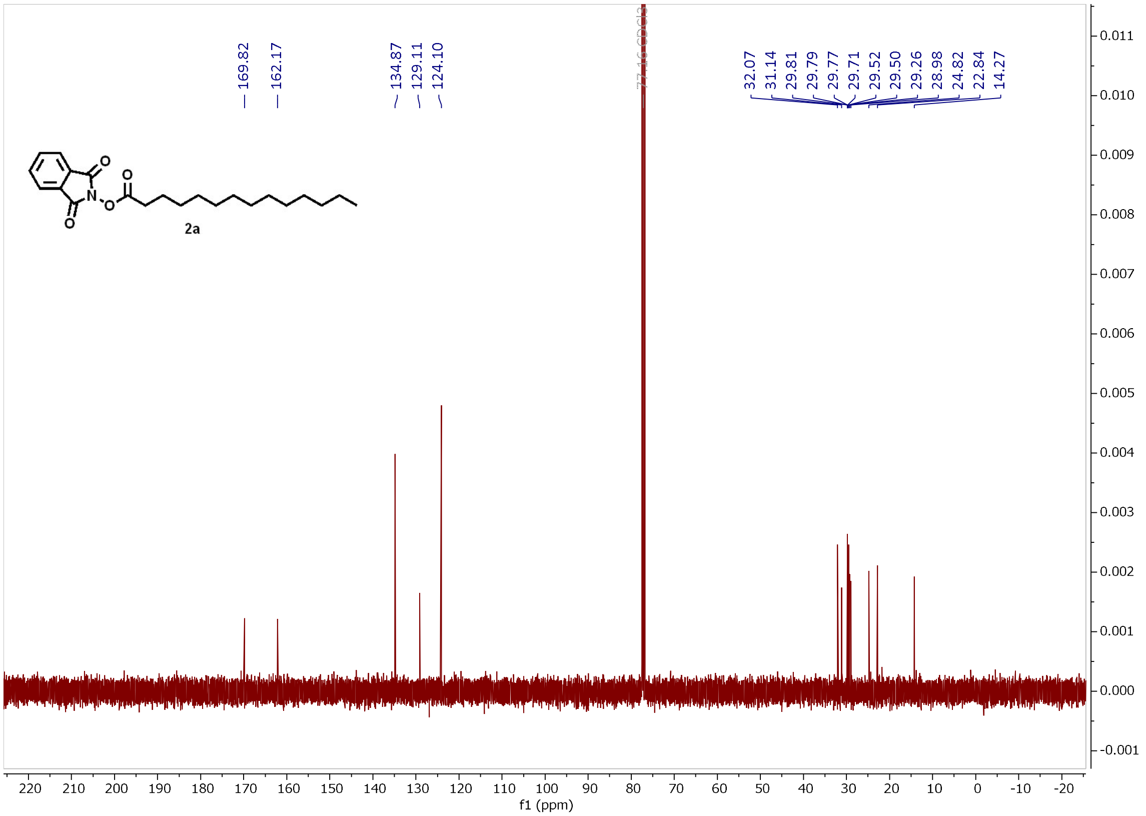

Supplementary Fig. 52. ^{13}C NMR (100 MHz, $CDCl_3$) of compound **2a**.

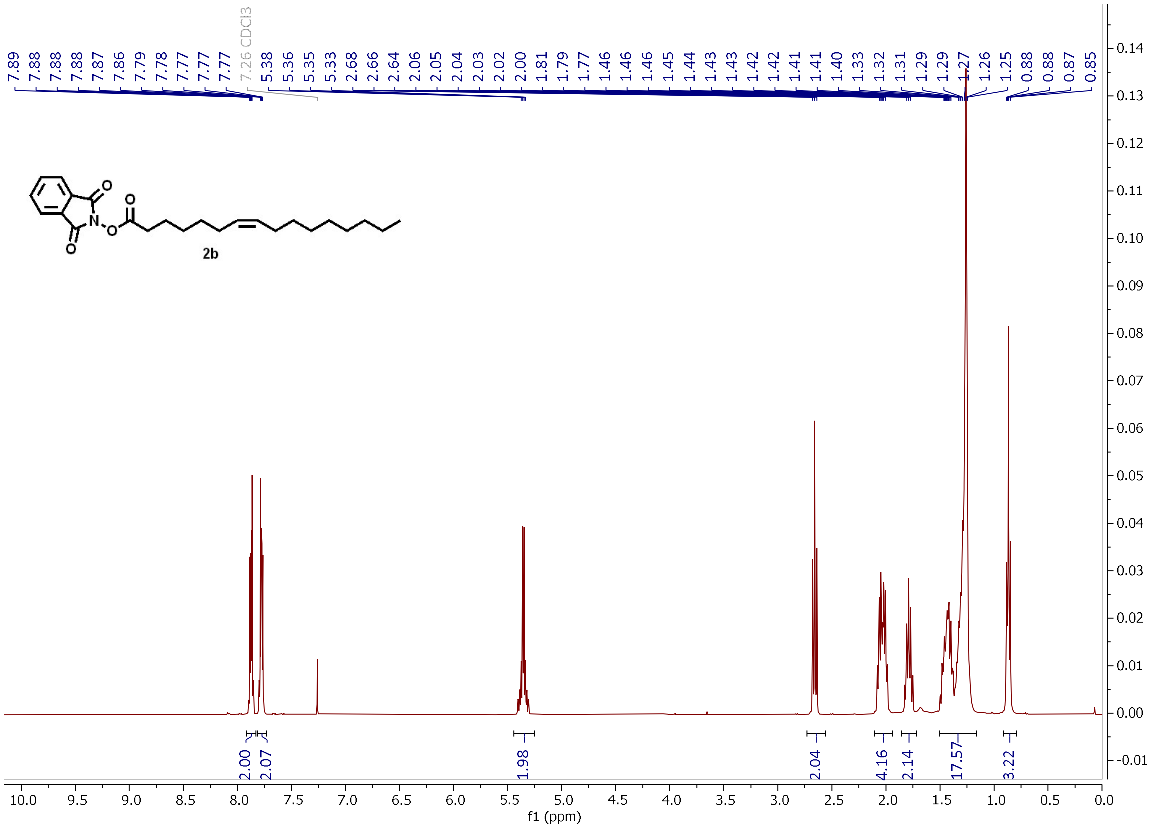

Supplementary Fig. 53. ^{1}H NMR (400 MHz, $CDCl_3$) of compound **2b.**

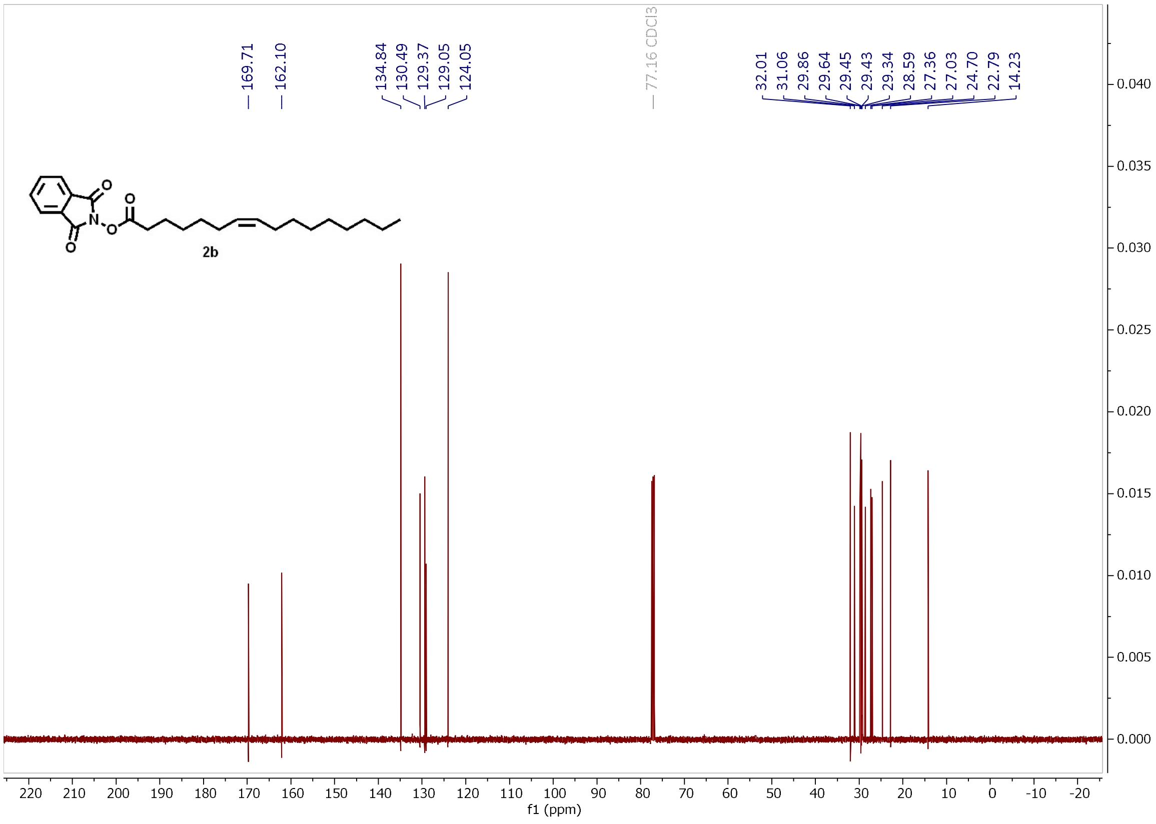

Supplementary Fig. 54. ^{13}C NMR (100 MHz, $CDCl_3$) of compound **2b**.

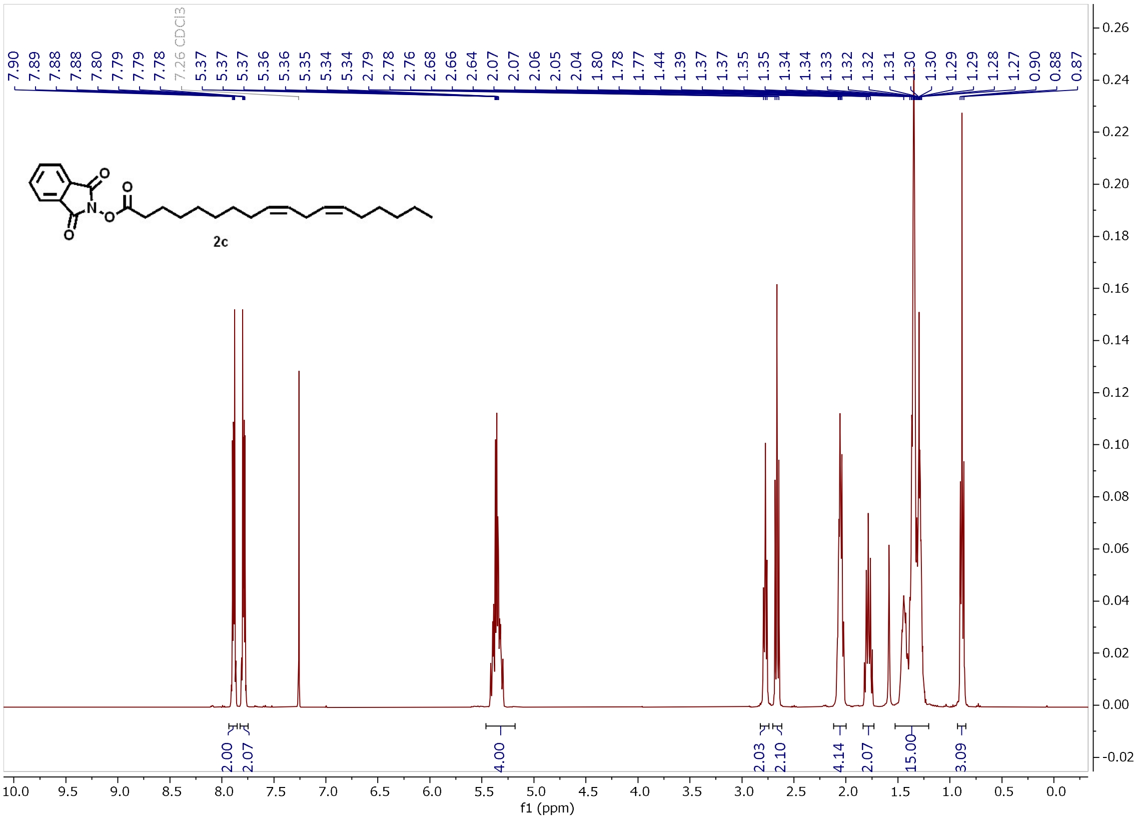

Supplementary Fig. 55. ^{1}H NMR (400 MHz, $CDCl_3$) of compound **2c.**

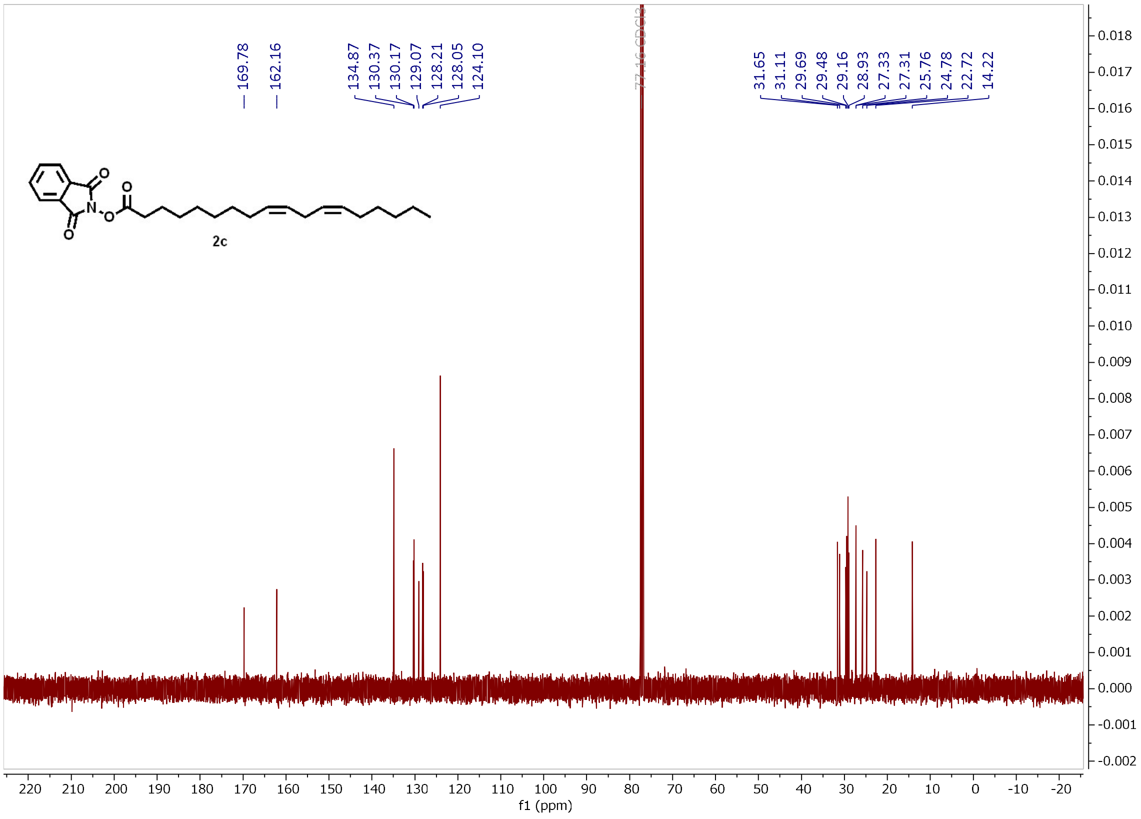

Supplementary Fig. 56. ^{13}C NMR (100 MHz, $CDCl_3$) of compound **2c**.

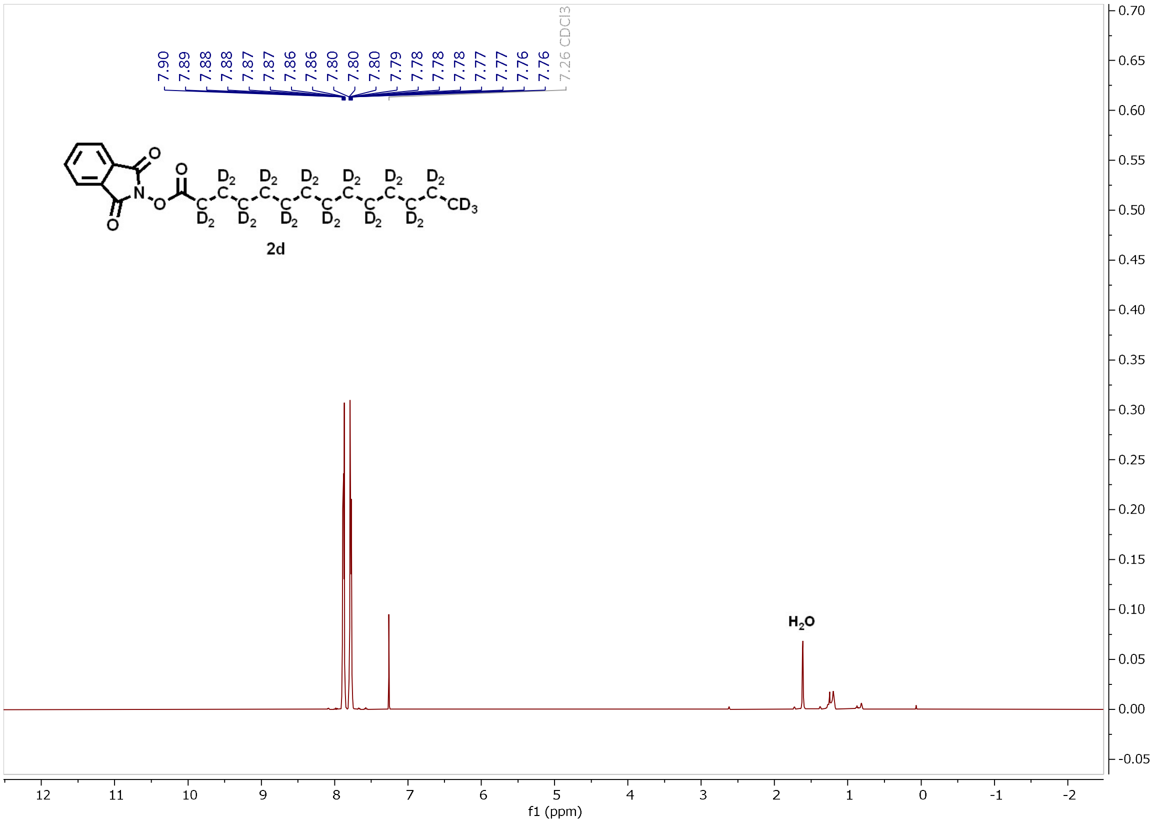

Supplementary Fig. 57. ^{1}H NMR (400 MHz, $CDCl_3$) of compound **2d.**

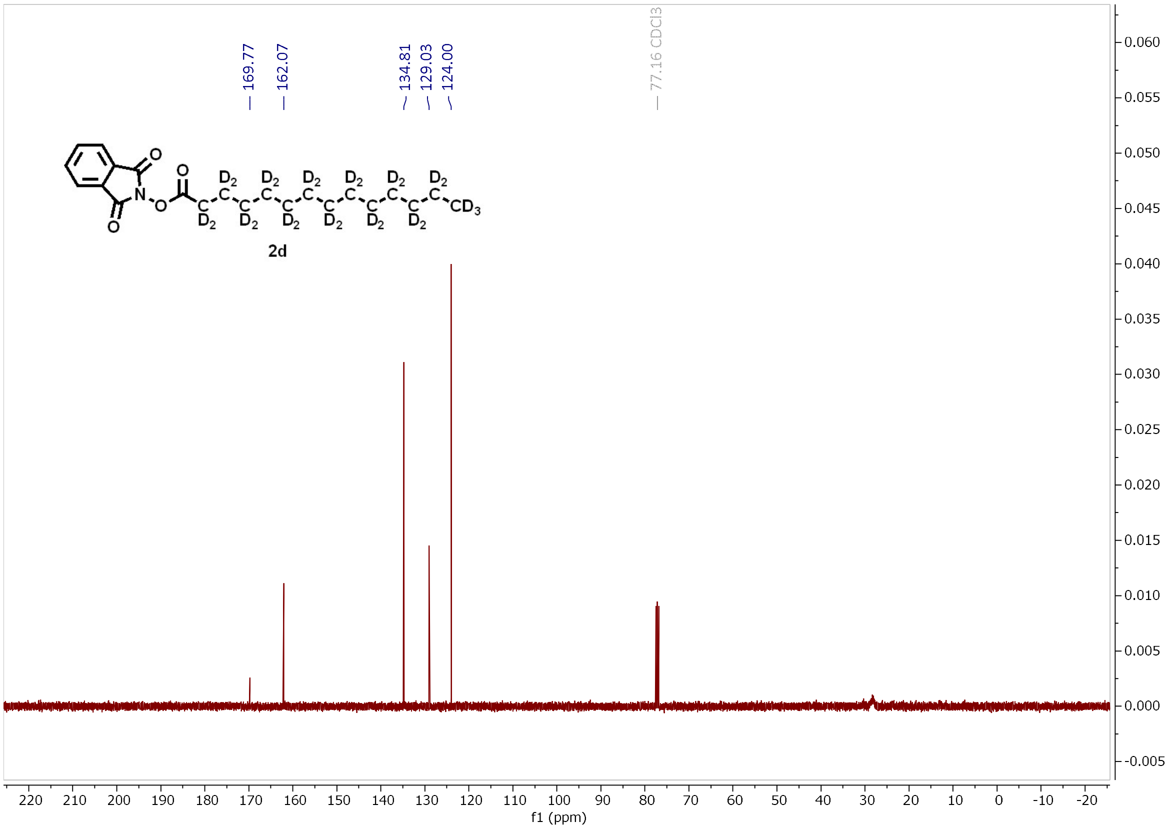

Supplementary Fig. 58. ^{13}C NMR (100 MHz, $CDCl_3$) of compound **2d**.

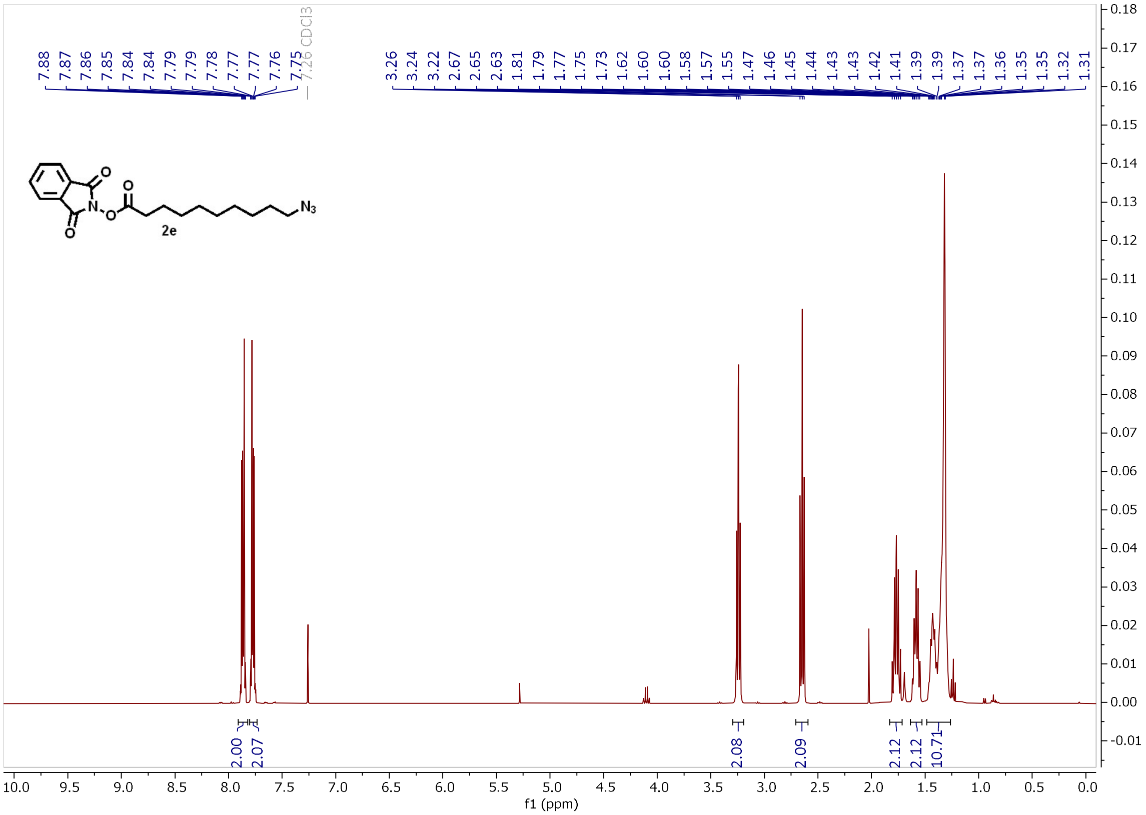

Supplementary Fig. 59. ^{1}H NMR (400 MHz, $CDCl_3$) of compound **2e.**

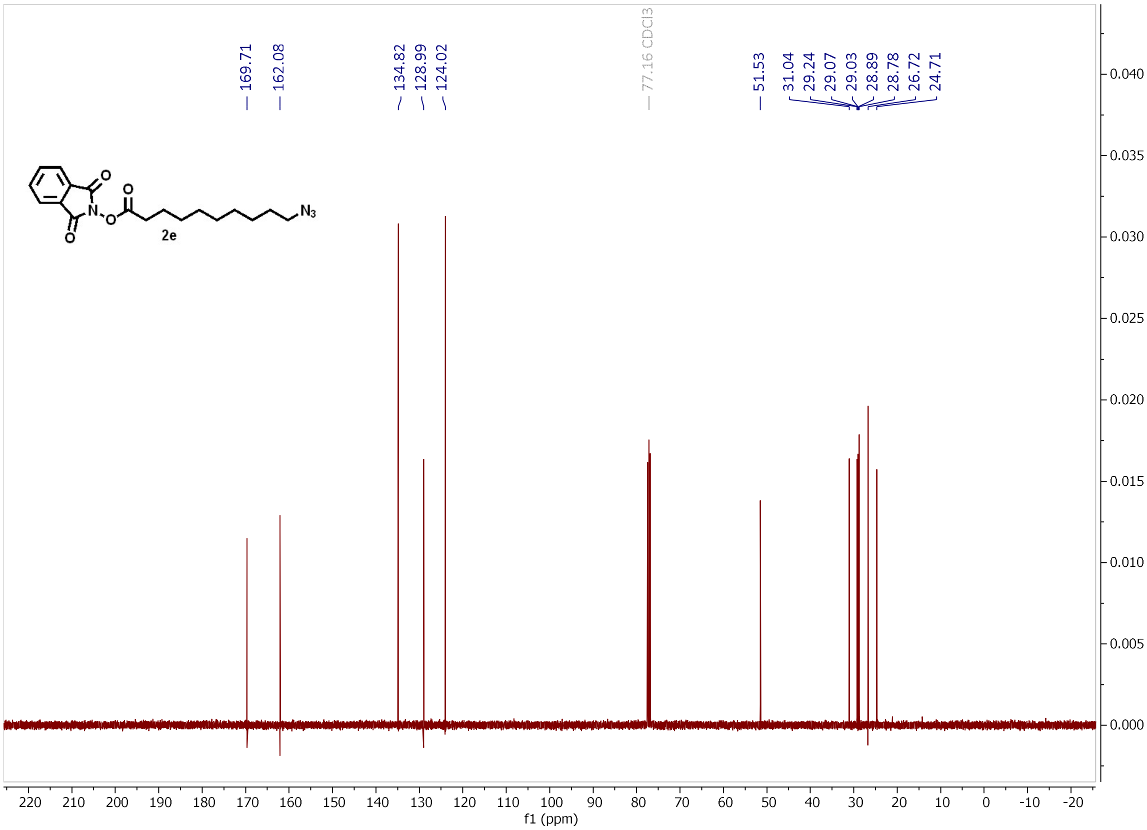

Supplementary Fig. 60. ^{13}C NMR (100 MHz, $CDCl_3$) of compound **2e**.

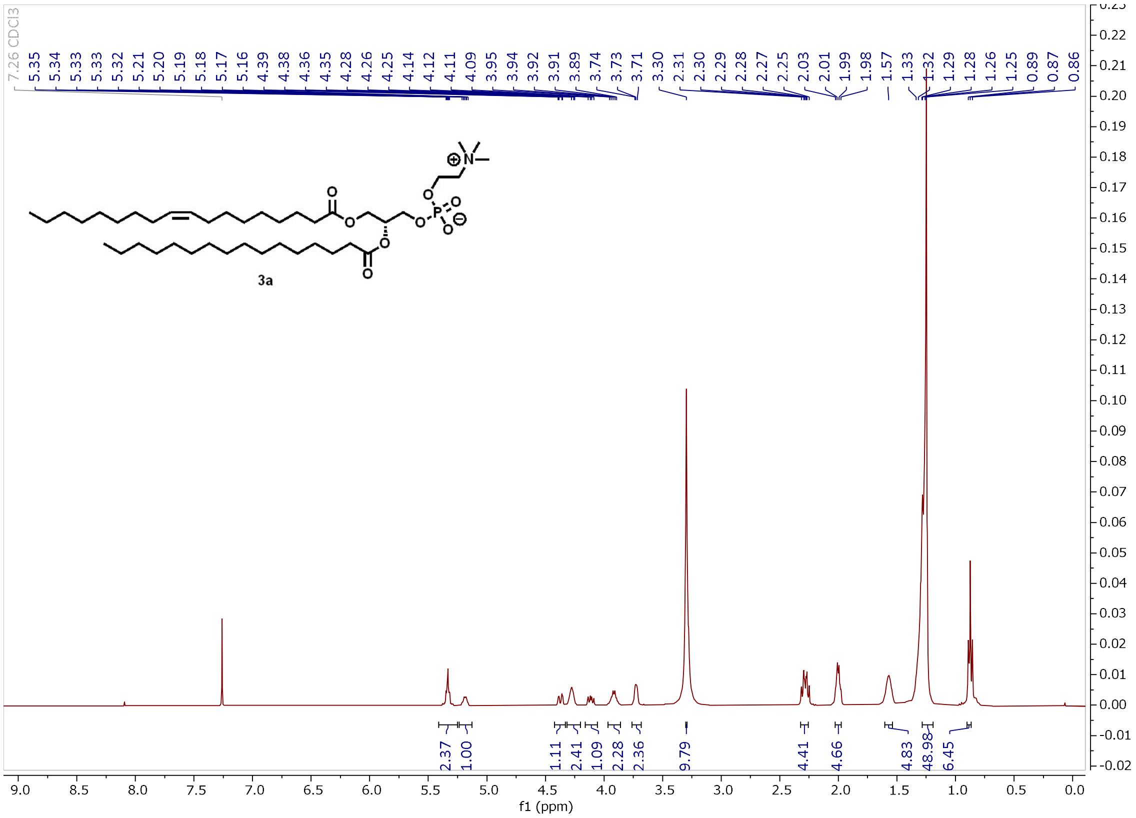

Supplementary Fig. 61. ^{1}H NMR (400 MHz, $CDCl_3$) of compound **3a.**

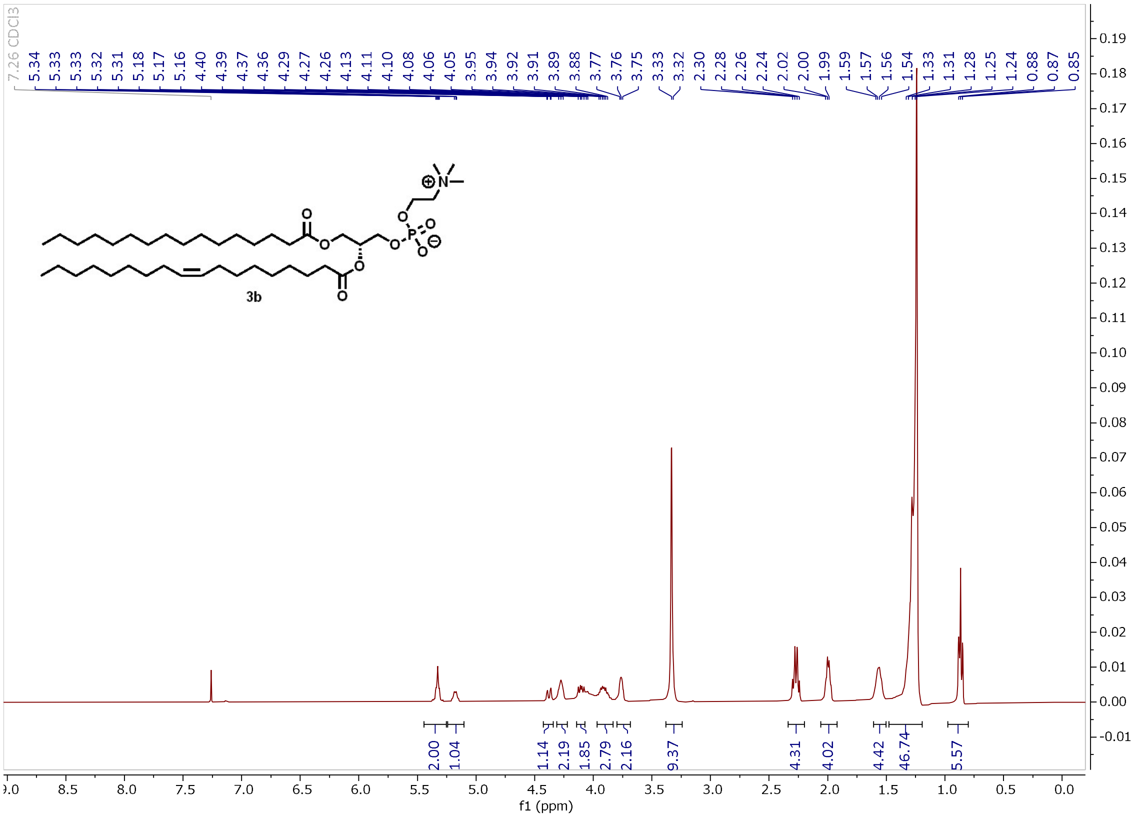

Supplementary Fig. 62. ^{1}H NMR (400 MHz, $CDCl_3$) of compound **3b.**

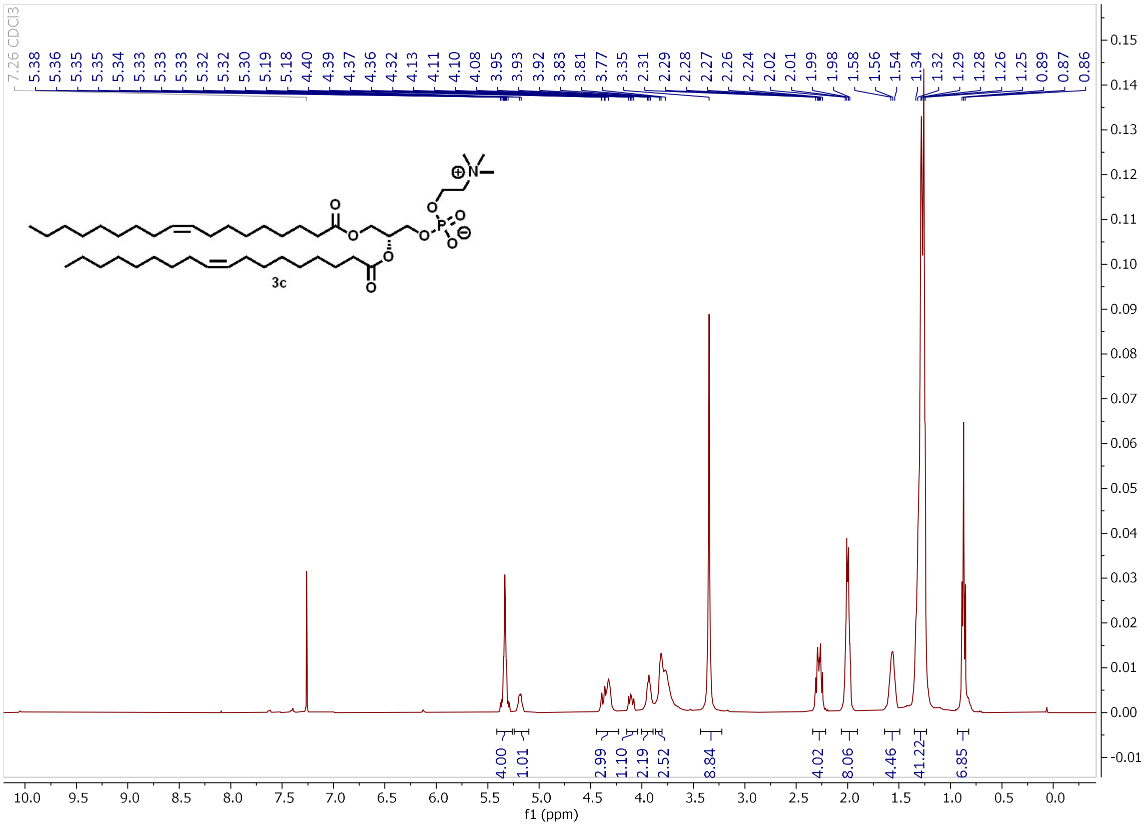

Supplementary Fig. 63. ^{1}H NMR (400 MHz, $CDCl_3$) of compound **3c.**

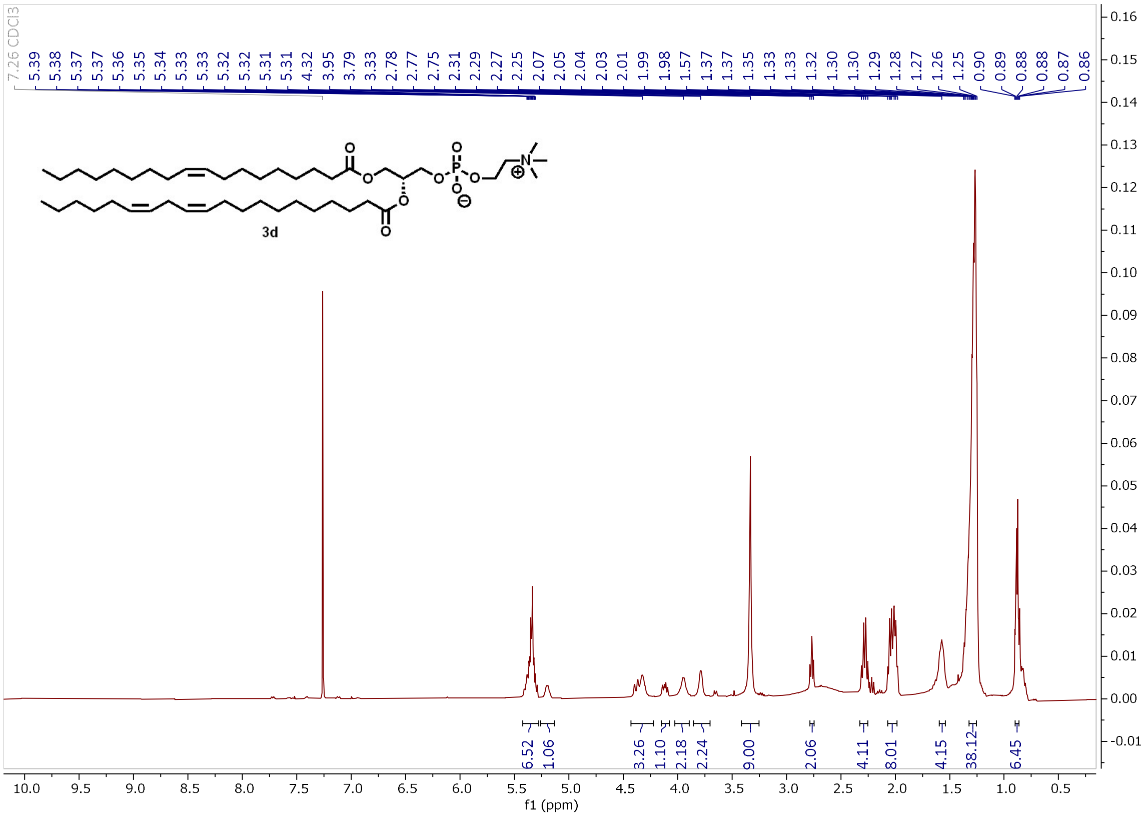

Supplementary Fig. 64. ^{1}H NMR (400 MHz, $CDCl_3$) of compound **3d.**

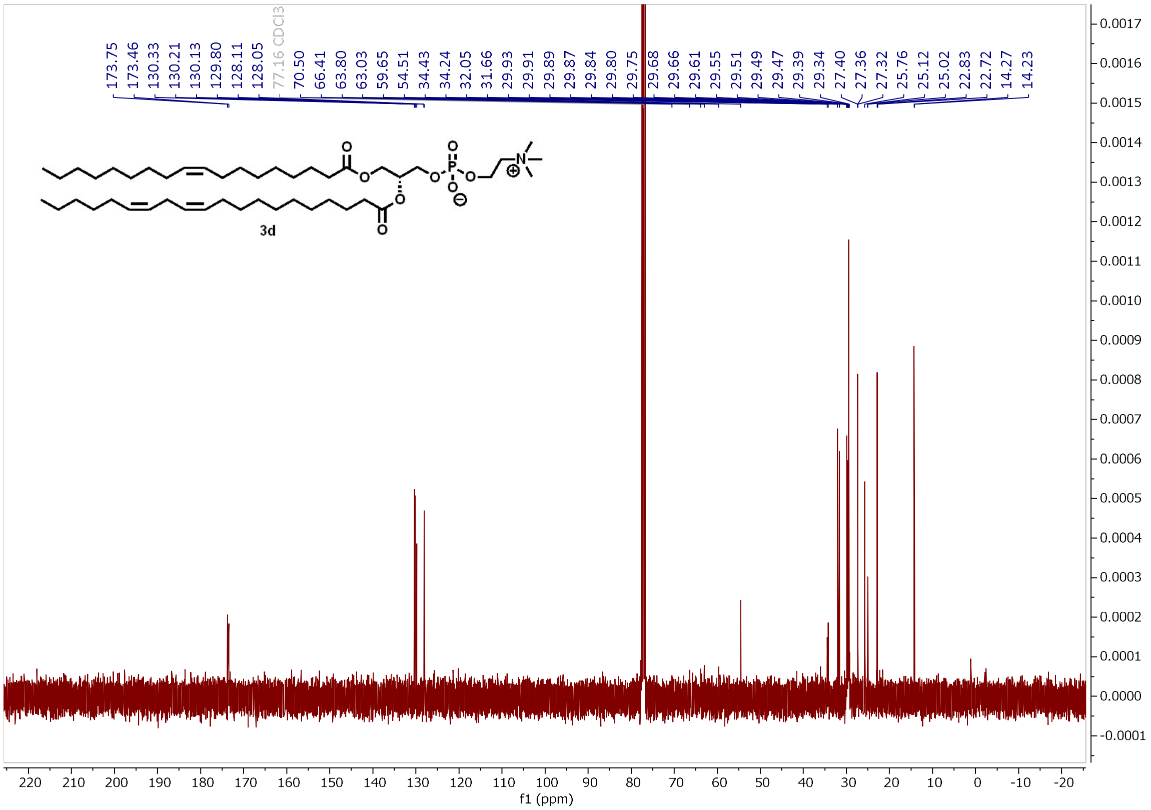

Supplementary Fig. 65. ^{13}C NMR (100 MHz, $CDCl_3$) of compound **3d**.

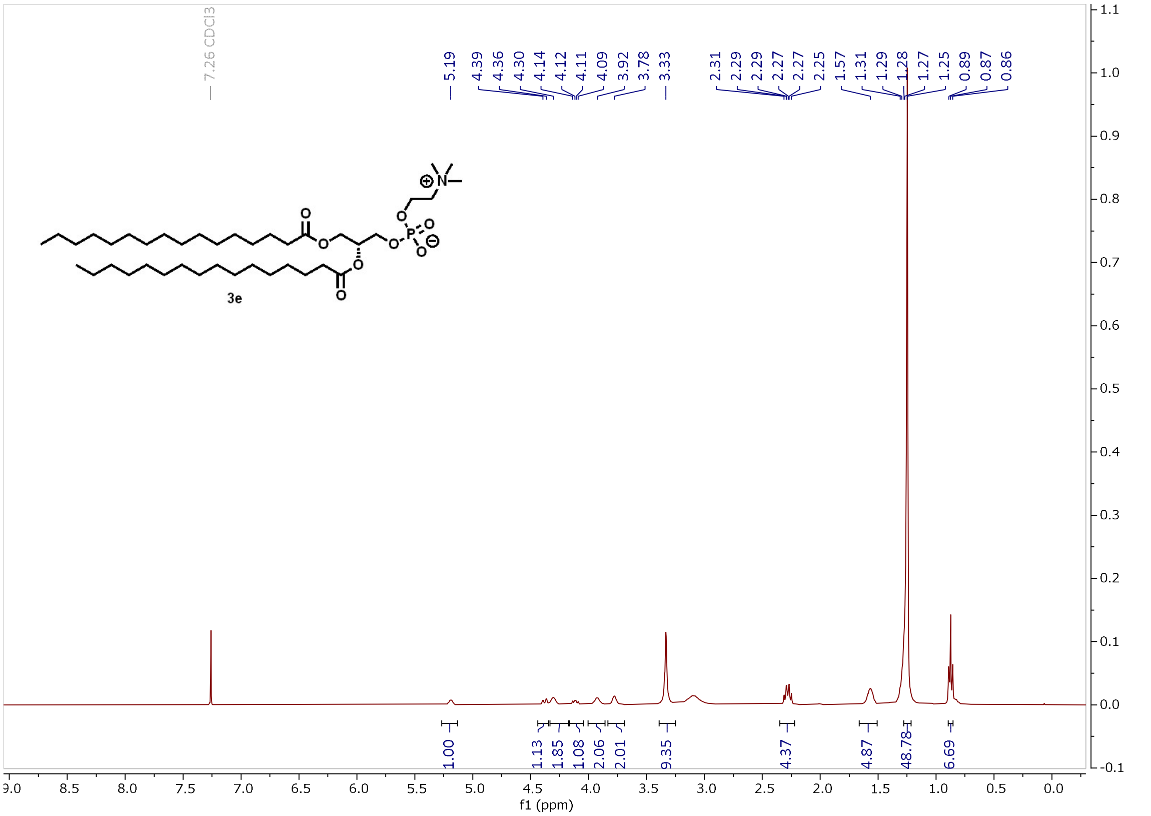

Supplementary Fig. 66. ^{1}H NMR (400 MHz, $CDCl_3$) of compound **3e.**

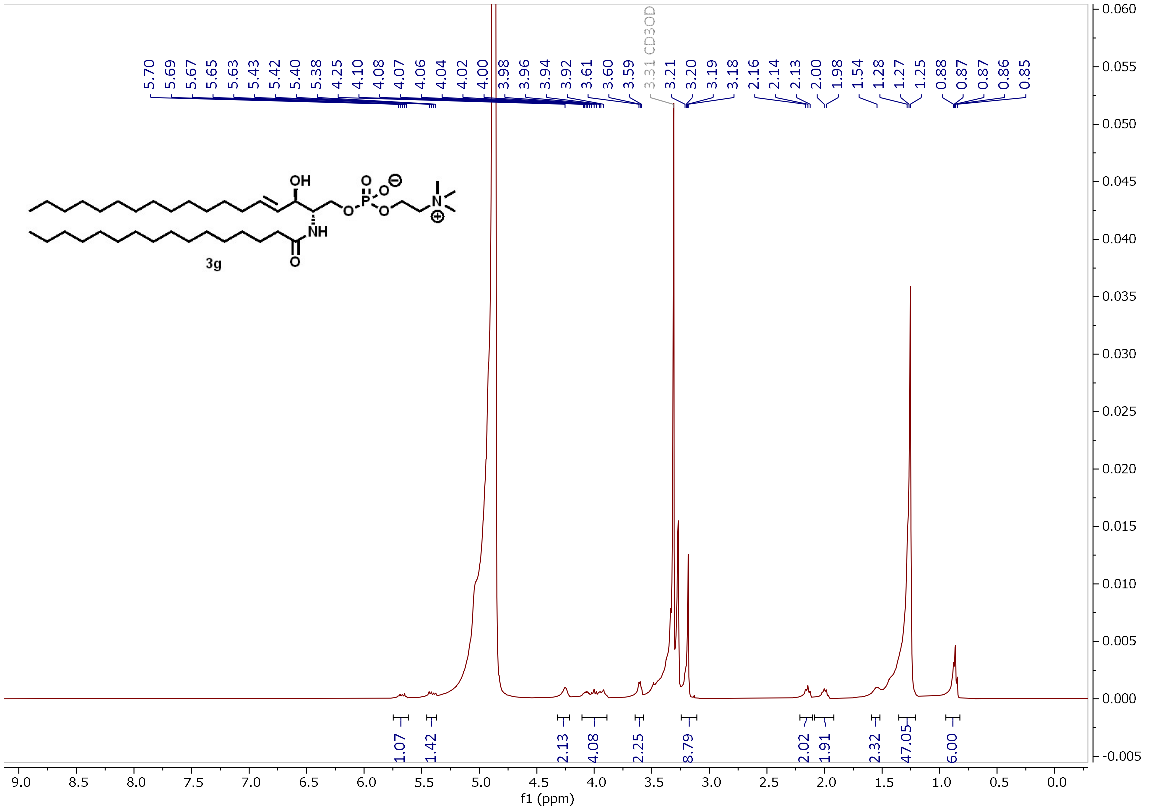

Supplementary Fig. 67. ^{1}H NMR (400 MHz, CD_3OD) of compound **3g.**

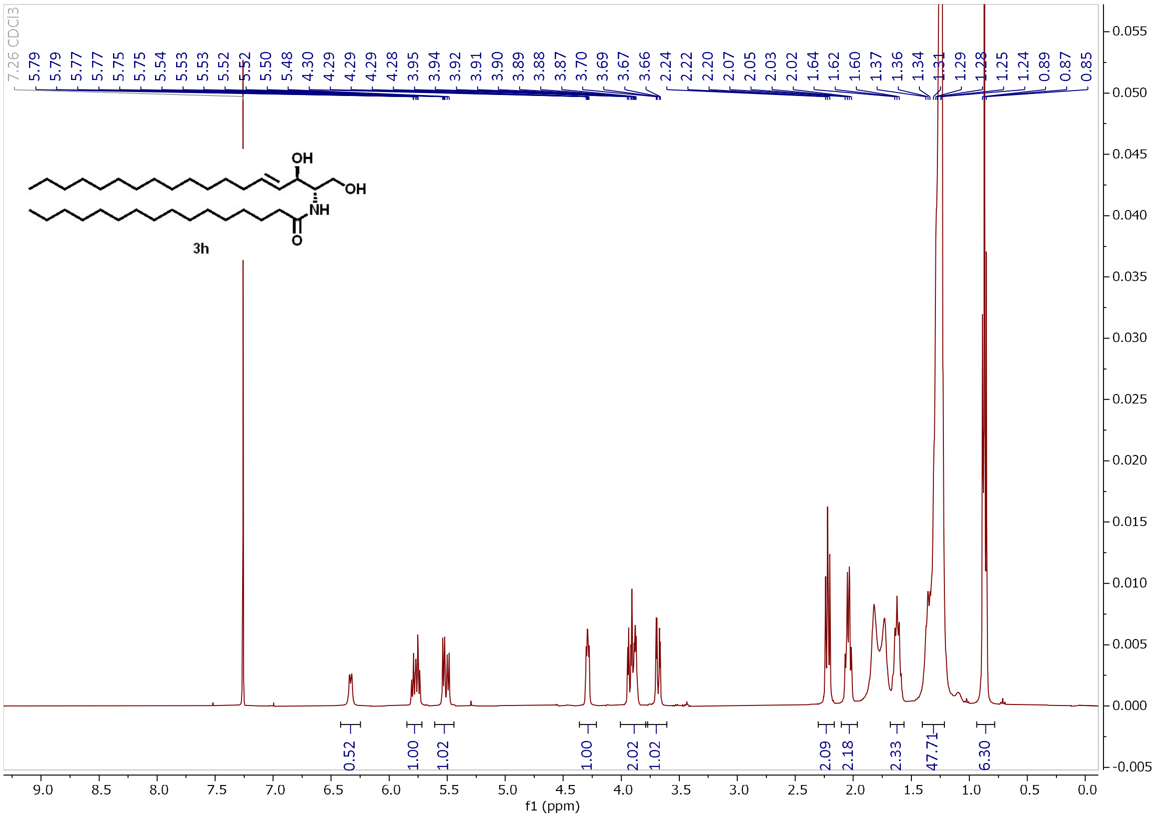

Supplementary Fig. 68. ^{1}H NMR (400 MHz, $CDCl_3$) of compound **3h.**

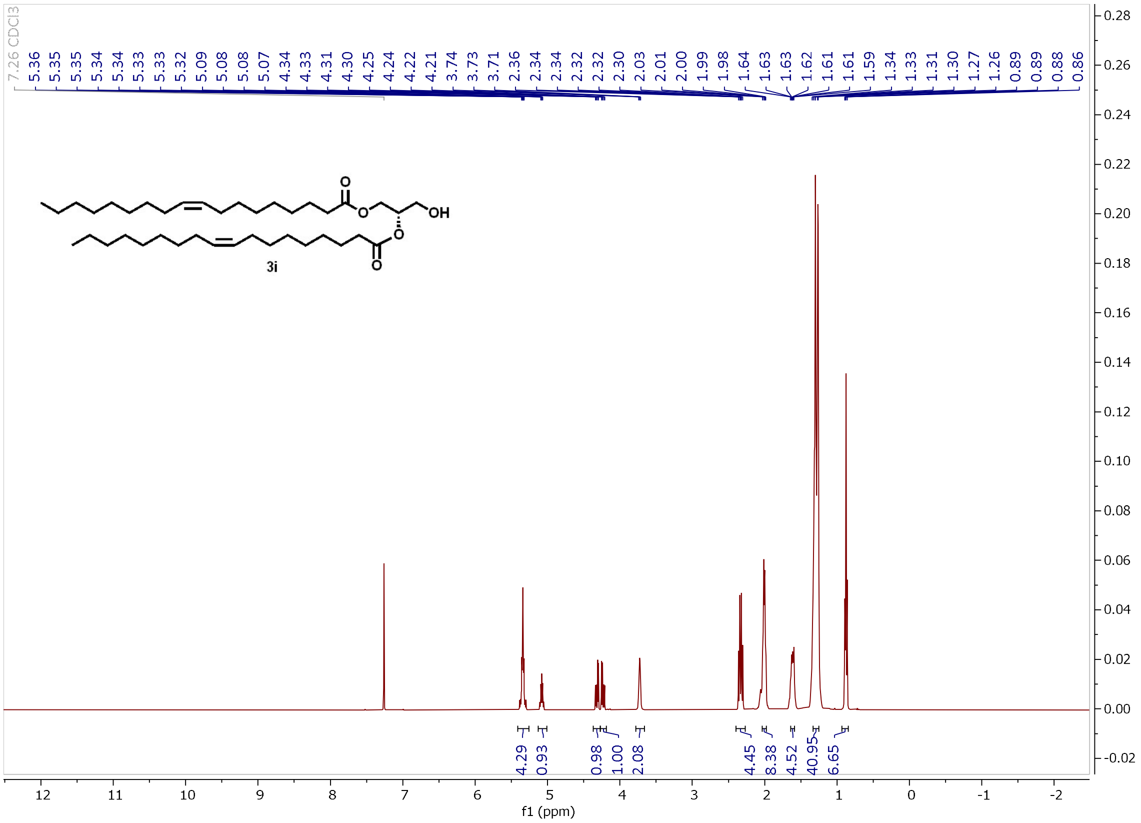

Supplementary Fig. 69. ^{1}H NMR (400 MHz, $CDCl_3$) of compound **3i.**

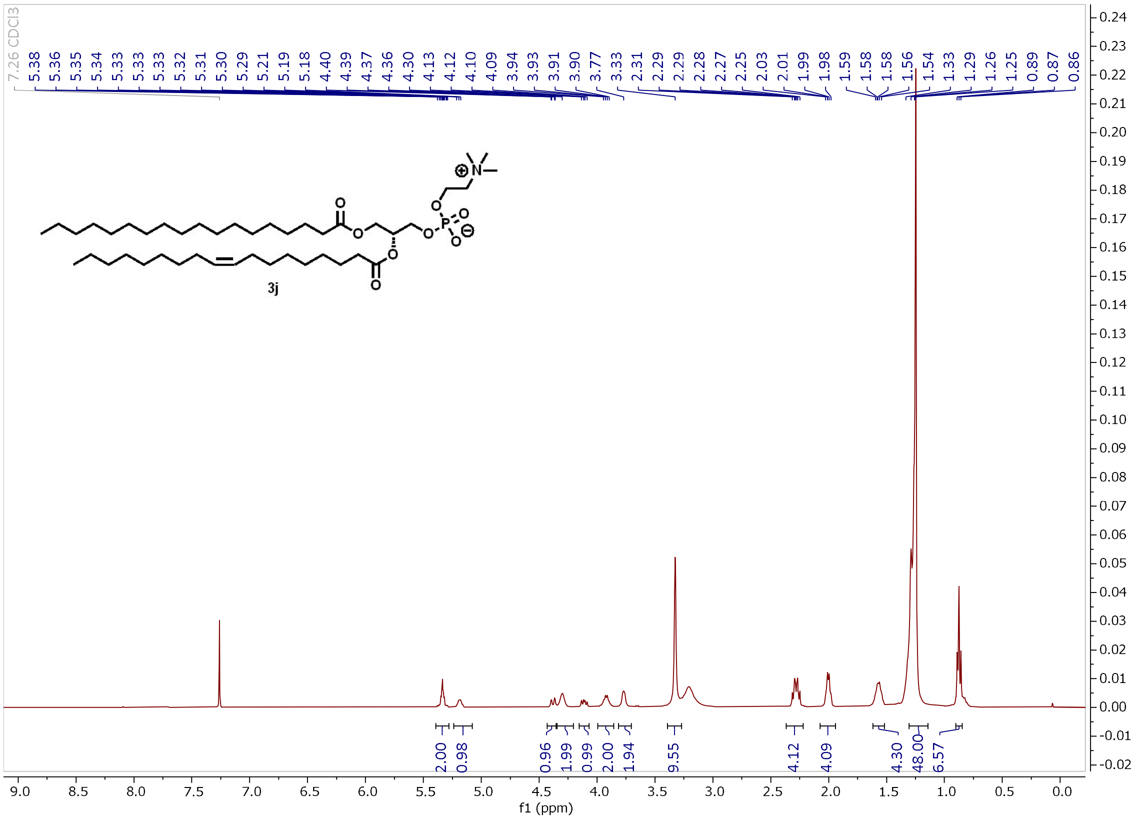

Supplementary Fig. 70. ^{1}H NMR (400 MHz, $CDCl_3$) of compound **3j.**

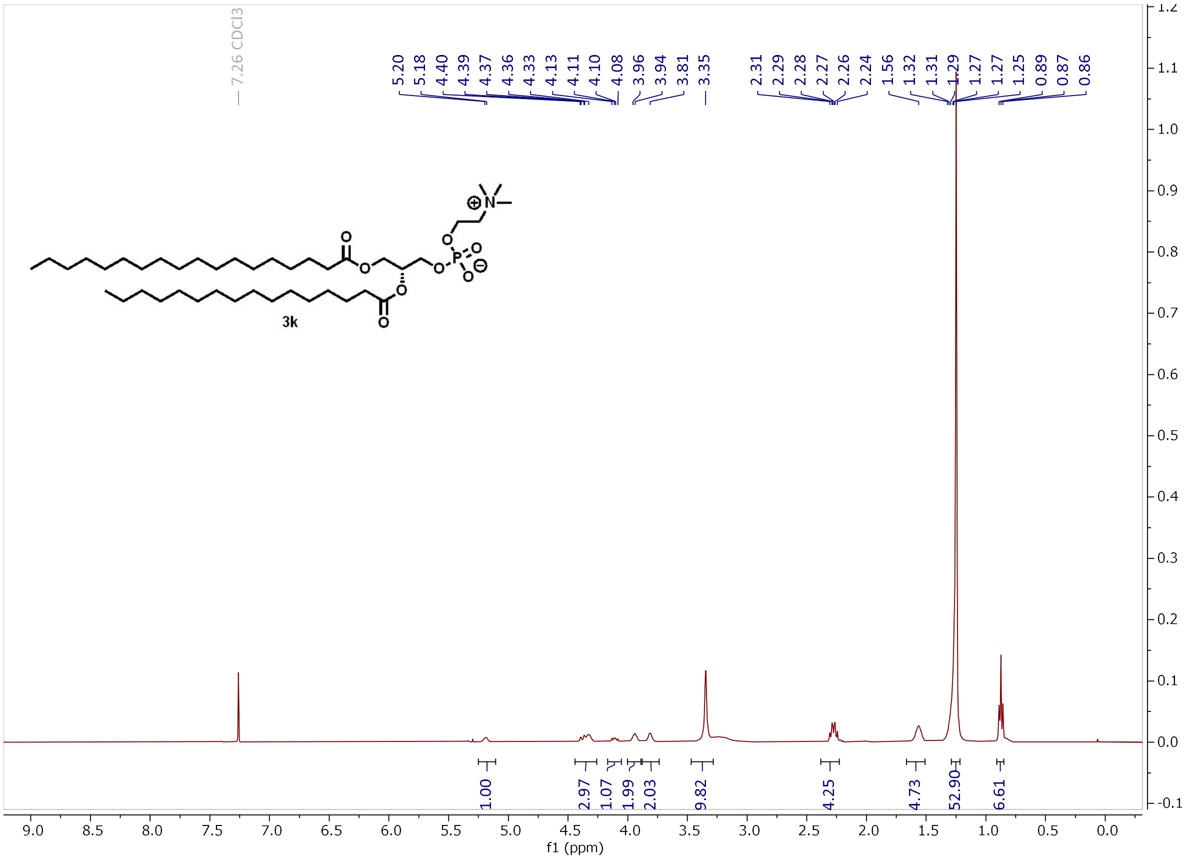

Supplementary Fig. 71. ^{1}H NMR (400 MHz, $CDCl_3$) of compound **3k.**

21. Supplementary References

1. Lampkin, P. P., Thompson, B. J., Gellman, S. H. Versatile Open-Source photoreactor architecture for photocatalysis across the visible spectrum. *Org. Lett.* **23**, 5277-5281 (2021).

2. Andringa, R. L. H., Jonker, M., Minnaard, A. J. Synthesis of phosphatidic acids via cobalt(salen) catalyzed epoxide ring-opening with dibenzyl phosphate. *Org. Biomol. Chem.* **20**, 2200-2204 (2022).

3. Stamatov, S. D., Stawinski, J. Regioselective and stereospecific acylation across oxirane- and silyloxy systems as a novel strategy to the synthesis of enantiomerically pure mono-, di- and triglycerides. *Org. Biomol. Chem.* **5**, 3787-3800 (2007).

4. Liu L. *et al.*, Enzyme-free synthesis of natural phospholipids in water. *Nat. Chem.* **12**, 1029-1034 (2020).

5. Yamamoto, T., Hasegawa, H., Hakogi, T., Katsumura, S. Versatile Synthetic Method for sphingolipids and functionalized sphingosine derivatives via olefin cross metathesis. *Org. Lett.* **8**, 5569-5572 (2006).

6. Gorantla, J. N., Santhi, M., Hua, Y., Ketudat Cairns, J. R. Total synthesis of ceramides and β-O-glucosylceramides via intramolecular fatty acyl group migration. *New J. Chem.* **46**, 3270-3276 (2022).

7. Mallik, S., Prasad, R., Bhattacharya, A., Sen, P. Synthesis of phosphatidylserine and its stereoisomers: their role in activation of blood coagulation. *ACS Med. Chem. Lett.* **9**, 434-439 (2018).

8. Chowdhury, R. et al. Decarboxylative alkyl coupling promoted by NADH and blue light. *J. Am. Chem. Soc.* **142**, 20143-20151 (2020).

9. Okada, K., Okamoto, K., Morita, N., Okubo, K. & Oda, M. Photosensitized decarboxylative Michael addition through N-(acyloxy)phthalimides via an electron-transfer mechanism. *J. Am. Chem. Soc.* **113**, 9401-9402 (1991).

10. Suloway, C., Pulokas, J., Felllmann, D., Cheng, A., Guerra, F., Quispe, J., Stagg, S., Potter, C. S., Carragher, B. Automated molecular microscopy: the new Leginon system. *J. Struct. Biol.* **151**, 41-60 (2005).

11. Punjani, A., Rubinstein, J. L., Fleet, D. J., Brubaker, M. A. cryoSPARC: algorithms for rapid unsupervised cryo-EM structure determination. *Nat. Methods* **14**, 290-296 (2017).

12. Autour, A., Jeng, S. C. Y., Cawte, A. D., Abdolahzadeh, A., Galli, A., Panchapakesan, S. S. S., Rueda, D., Ryckelynck, M., Unrau, P. J. Fluorogenic RNA Mango aptamers for imaging small non-coding RNAs in mammalian cells. *Nat. Commun.* **9**, 656 (2018).

13. Bligh, E. G., Dyer, W. J. A rapid method of total lipid extraction and purification. *Can. J. Biochem. Physiol.* **37**, 911-917 (1959).
