# Supplementary material for: Photochemical synthesis of natural lipids in artificial and living cells
Source: Nat Commun. 2025 May 31;16:5068. doi: 10.1038/s41467-025-60358-4 (PMC12126527; doi:10.1038/s41467-025-60358-4)
Supplement: Supplementary file 2 — Description of Additional Supplementary Files [file 41467_2025_60358_MOESM2_ESM.pdf]

Description of Additional Supplementary Movies

Supplementary Movie 1: POPC vesicle formation under green light over 30 min

Supplementary Movie 2: in situ formed POPC vesicles showing growth under green light over 15 min

Supplementary Movie 3: in situ formed POPC vesicles showing growth and undergoing morphological changes under green light over 15 min

Supplementary Movie 4: a mixture of NHPI ester **2b** and lysolipid **1b** kept in the dark over 30 min as a control experiment

Supplementary Movie 5: a mixture of NHPI ester **2b** and lysolipid **1b** in absence of eosin Y under green light over 30 min as a control experiment

Supplementary Movie 6: in situ formed POPC vesicles in absence of green light over 20 min as a control experiment
