# Supplementary material for: Photochemical synthesis of natural lipids in artificial and living cells
Source: Nat Commun. 2025 May 31;16:5068. doi: 10.1038/s41467-025-60358-4 (PMC12126527; doi:10.1038/s41467-025-60358-4)
Supplement: Supplementary file 9 — Reporting Summary [file 41467_2025_60358_MOESM9_ESM.pdf]

nature portfolio

Corresponding author(s): Neal K. Devaraj

Last updated by author(s): May 1, 2025

Reporting Summary

Nature Portfolio wishes to improve the reproducibility of the work that we publish. This form provides structure for consistency and transparency in reporting. For further information on Nature Portfolio policies, see our Editorial Policies and the Editorial Policy Checklist.

Statistics

For all statistical analyses, confirm that the following items are present in the figure legend, table legend, main text, or Methods section.

n/a	Confirmed	
[ ]	[x]	The exact sample size (n) for each experimental group/condition, given as a discrete number and unit of measurement
[ ]	[x]	A statement on whether measurements were taken from distinct samples or whether the same sample was measured repeatedly
[ ]	[x]	The statistical test(s) used AND whether they are one- or two-sided<br>*Only common tests should be described solely by name; describe more complex techniques in the Methods section.*
[x]	[ ]	A description of all covariates tested
[x]	[ ]	A description of any assumptions or corrections, such as tests of normality and adjustment for multiple comparisons
[ ]	[x]	A full description of the statistical parameters including central tendency (e.g. means) or other basic estimates (e.g. regression coefficient) AND variation (e.g. standard deviation) or associated estimates of uncertainty (e.g. confidence intervals)
[ ]	[x]	For null hypothesis testing, the test statistic (e.g. F, t, r) with confidence intervals, effect sizes, degrees of freedom and P value noted<br>*Give P values as exact values whenever suitable.*
[x]	[ ]	For Bayesian analysis, information on the choice of priors and Markov chain Monte Carlo settings
[x]	[ ]	For hierarchical and complex designs, identification of the appropriate level for tests and full reporting of outcomes
[x]	[ ]	Estimates of effect sizes (e.g. Cohen's d, Pearson's r), indicating how they were calculated

Our web collection on statistics for biologists contains articles on many of the points above.

Software and code

Policy information about availability of computer code

Data collection	Tecan infinite F200 plate reader was used to collect the data in the absorbance measurement, ZEN Blue 2.3 imaging software (Carl Zeiss Microscopy GmbH, Germany) was used to obtain images, microscopy images were processed using ImageJ (Fiji, version:2.0.0-rc-66/1.56b), and MestReNova 12.0.0 suite was used to process NMR spectra.
Data analysis	Statistical analysis was performed using GraphPad Prism 10 or Microsoft Excel (Microsoft 365 for Apps for enterprise).

For manuscripts utilizing custom algorithms or software that are central to the research but not yet described in published literature, software must be made available to editors and reviewers. We strongly encourage code deposition in a community repository (e.g. GitHub). See the Nature Portfolio guidelines for submitting code & software for further information.

Data

Policy information about availability of data

All manuscripts must include a data availability statement. This statement should provide the following information, where applicable:

- Accession codes, unique identifiers, or web links for publicly available datasets
- A description of any restrictions on data availability
- For clinical datasets or third party data, please ensure that the statement adheres to our policy

All data are available in the main text, supplementary materials and from the corresponding author(s) upon request. Source Data are provided with this paper.

Human research participants

Policy information about studies involving human research participants and Sex and Gender in Research.

Reporting on sex and gender	Not applicable.
Population characteristics	Not applicable.
Recruitment	Not applicable.
Ethics oversight	Not applicable.

Note that full information on the approval of the study protocol must also be provided in the manuscript.

Field-specific reporting

Please select the one below that is the best fit for your research. If you are not sure, read the appropriate sections before making your selection.

☒ Life sciences ☐ Behavioural & social sciences ☐ Ecological, evolutionary & environmental sciences

For a reference copy of the document with all sections, see nature.com/documents/nr-reporting-summary-flat.pdf

Life sciences study design

All studies must disclose on these points even when the disclosure is negative.

Sample size	Sample size was determined based on previous experience and experimental feasibility.
Data exclusions	No data were excluded from the analyses.
Replication	Relevant experiments were replicated successfully multiple times. For experiments that involve quantification, statistical analysis was used to confirm significance of the experiment. Relevant p-values are provided in the respective figure.<br>Relevant experiments were replicated successfully multiple times. For experiments that involve quantification, statistical analysis was used to confirm significance of the experiment. Relevant p-values are provided in the respective figure.
Randomization	The samples were allocated into experimental groups randomly.
Blinding	Not applicable.

Reporting for specific materials, systems and methods

We require information from authors about some types of materials, experimental systems and methods used in many studies. Here, indicate whether each material, system or method listed is relevant to your study. If you are not sure if a list item applies to your research, read the appropriate section before selecting a response.

Materials & experimental systems

n/a	Involved in the study	
☐	☒	Antibodies
☐	☒	Eukaryotic cell lines
☒	☐	Palaeontology and archaeology
☒	☐	Animals and other organisms
☒	☐	Clinical data
☒	☐	Dual use research of concern

Methods

n/a	Involved in the study	
☒	☐	ChIP-seq
☒	☐	Flow cytometry
☒	☐	MRI-based neuroimaging

Antibodies

Antibodies used	Anti-Actin (8H10D10) antibody (1:1000) (#3700, Cell Signaling Technology), Anti-Phospho-(Ser) PKC substrate antibody (1:1000) (#2261, Cell Signaling Technology).
Validation	All antibodies used in this study have been previously validated by the vendor. Vendor information and relevant literature citations

are available from the respective vendor's website. Vendor product codes for each antibody are listed in the methods section.

Eukaryotic cell lines

Policy information about cell lines and Sex and Gender in Research

Cell line source(s)	Human HeLa cells (ATCC, CCL-2.2)
Authentication	ATCC has profiled cell lines using polymorphic short tandem repeat (STR) locic (TH01, TPOX, vWA, CSF1PO, D16S539, D7S820, D13S317 and D5S818) plus Amelogenin for gender identification.
Mycoplasma contamination	All cell lines in this study are tested negative for mycoplasma contamination
Commonly misidentified lines (See ICLAC register)	Cell lines in this study is not known to be cross-contaminated or misidentified in the ICLAC database
